# Neurotransmitter signaling regulates distinct phases of multimodal human interneuron migration

**DOI:** 10.15252/embj.2021108714

**Published:** 2021-10-18

**Authors:** Sunanjay Bajaj, Joshua A Bagley, Christoph Sommer, Abel Vertesy, Sakurako Nagumo Wong, Veronica Krenn, Julie Lévi‐Strauss, Juergen A Knoblich

**Affiliations:** ^1^ Institute of Molecular Biotechnology of the Austrian Academy of Sciences (IMBA) Vienna Austria; ^2^ University of Heidelberg Heidelberg Germany; ^3^ Institute of Science and Technology Austria (IST Austria) Klosterneuburg Austria; ^4^ Medical University of Vienna Vienna Austria; ^5^ Present address: a:head bio AG Vienna Austria

**Keywords:** cerebral organoid fusions, cortical interneuron migration, human brain development, live cell imaging, neurotransmitter signaling pathways, Neuroscience

## Abstract

Inhibitory GABAergic interneurons migrate over long distances from their extracortical origin into the developing cortex. In humans, this process is uniquely slow and prolonged, and it is unclear whether guidance cues unique to humans govern the various phases of this complex developmental process. Here, we use fused cerebral organoids to identify key roles of neurotransmitter signaling pathways in guiding the migratory behavior of human cortical interneurons. We use scRNAseq to reveal expression of GABA, glutamate, glycine, and serotonin receptors along distinct maturation trajectories across interneuron migration. We develop an image analysis software package, TrackPal, to simultaneously assess 48 parameters for entire migration tracks of individual cells. By chemical screening, we show that different modes of interneuron migration depend on distinct neurotransmitter signaling pathways, linking transcriptional maturation of interneurons with their migratory behavior. Altogether, our study provides a comprehensive quantitative analysis of human interneuron migration and its functional modulation by neurotransmitter signaling.

## Graphical abstract

Live‐imaging in cerebral organoids combined with single‐cell transcriptomics and small‐molecule screening reveals distinct modes of human interneuron migration and their modulation by different neurotransmitters.

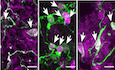

## Introduction

Formation of functional cortical circuits critically depends on balanced excitation and inhibition arising from excitatory glutamatergic projection neurons and inhibitory GABAergic interneurons (Tatti *et al*, 2017). Although glutamatergic neurons are generated and remain in the developing cortex, cortical interneurons migrate over a long distance into their target cortical destinations after being generated in the ganglionic eminences of the ventral telencephalon (Peyre *et al*, 2015). This long‐distance migration is dependent on extracellular molecular cues that guide and control the dynamics of migrating cells (Marín & Rubenstein, 2001). Neurotransmitters are well known for their function in neuronal communication through synaptic transmission between developed neurons, but they can also serve as extrinsic cues for migrating interneurons. Previous studies have reported diverse effects of various neurotransmitters on interneuron migration and cortical layer allocation in rodents (Berghuis *et al*, 2005; Avila *et al*, 2013; Murthy *et al*, 2014; Luhmann *et al*, 2015). However, long‐range interneuron migration in the developing human brain is prolonged when compared to rodent interneurons and may extend into postnatal stages of brain development (Arshad *et al*, 2016; Paredes *et al*, 2016), which may increase the susceptibility of migrating human cortical interneurons to extrinsic perturbations such as neurotransmitter imbalances during the course of migration (Lewis *et al*, 2005; Thompson *et al*, 2009; Galanopoulou, 2010). Furthermore, recent discoveries highlight human‐specific features of interneuron development, including unique progenitor (Zecevic *et al*, 2010) and interneuron subtypes (Boldog *et al*, 2018). Therefore, since there are no data using human systems, our aim was to conduct a comprehensive study of how neurotransmitter signaling modulates human interneuron migration.

Interneuron migration is a complex process encompassing periods of dynamic re‐arrangements in cellular morphology and multiphasic nuclear movements (Bellion *et al*, 2005). Previous analyses of interneuron migration have been limited to global migration pattern assessments which indirectly defined migration defects based on aberrant endpoint layer allocation (Stumm *et al*, 2003). Furthermore, migrating interneurons exhibit a diverse range of behaviors along their ventral–dorsal migration (Hatanaka *et al*, 2016). Thus, the characterization of intrinsic migration dynamics has been largely incomplete since defects in migration behavior were inferred from indirect analyses of postmortem mouse and human cortices (Batista‐Brito *et al*, 2008; Chen *et al*, 2017; Mayer *et al*, 2018; Hodge *et al*, 2019). The recent development of 3D *in vitro* human models of brain development enables the direct investigation of migration dynamics using time‐lapse imaging of human neurons. In particular, ventral–dorsal forebrain organoid fusions recapitulate the entire long‐distance migration route of cortical interneurons from the ventral progenitor regions into the dorsal cortex and therefore represent an ideal model system for the study of human interneuron migration (Bagley *et al*, 2017; Birey *et al*, 2017; Xiang *et al*, 2017).

Here, we used a cell type‐specific reporter to trace GABAergic interneurons within ventral–dorsal organoid fusions and isolate interneurons across their entire migratory route from ventral into dorsal organoid regions. We perform single‐cell transcriptomic analysis to identify maturation trajectories across ventral–dorsal migration and uncover characteristic expression patterns of neurotransmitter receptors genes. The function of expressed neurotransmitter signaling pathways was screened using large‐scale time‐lapse analysis of migrating interneurons and a novel track analysis software package, TrackPal. Various neurotransmitter signaling pathways exhibited pathway‐specific effects on the motility and guidance of migrating neurons. Moreover, interneuron migration modes were distinctly regulated by the various neurotransmitter signaling pathways. Overall, our study provides a comprehensive quantitative analysis of neurotransmitter regulation of multimodal human GABAergic interneuron migration.

## Results

### A DLXi56‐GFP reporter identifies migrating human interneurons

**Figure 1:**
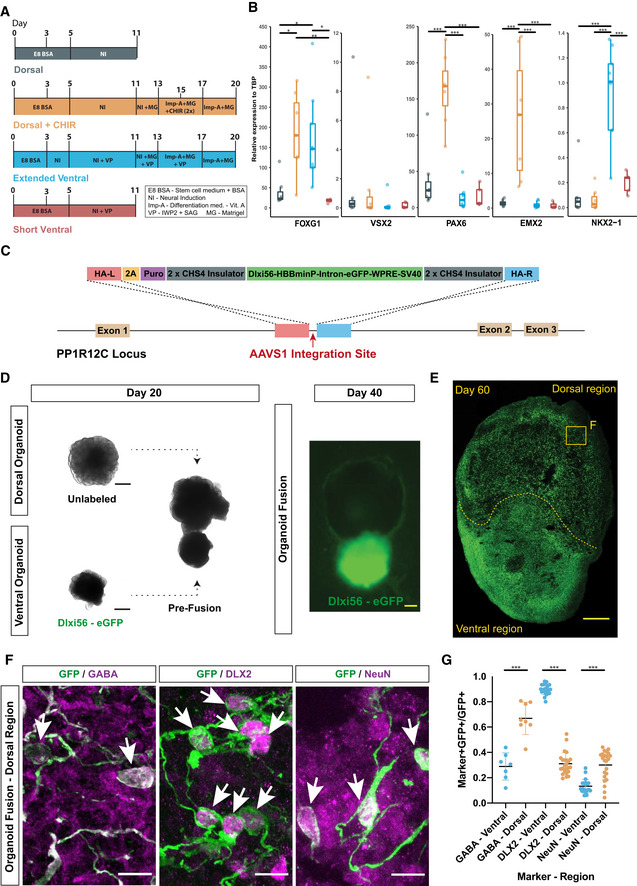
Labeling migrating human interneurons in cerebral organoid fusions Schematic overview of four different protocols for dorsal or ventral forebrain organoid generation. NI, neural induction medium; Imp‐A, improved differentiation medium without vitamin A; MG, Matrigel; VP, ventral patterning (2.5 μM IWP2 and 100 nM SAG).RT‐PCR analysis for forebrain (FOXG1), optic cup (VSX2), dorsal forebrain (PAX6 and EMX2), and ventral forebrain (NKX2‐1) identity in organoids. All expression values (2^−Δ^ *^C^* ^t^) calculated relative to reference gene TBP. Analysis was performed on 5–6 organoids from multiple independent differentiations—control (7), dorsal (7), extended ventral (9), and short ventral (5). Significance values, *< 0.05, **< 0.01, ***< 0.001. Statistical analysis was performed using one‐way ANOVA and post hoc Tukey’s comparison of means. The central band depicts the median, the boxes depict values between lower and upper quartile, and the whiskers display the minimum and maximum values.Schematic overview of TALEN‐based insertion of an interneuron‐specific DLXi56‐eGFP reporter into the AAVS1 locus.Overview of organoid fusion co‐culture method at Day 20 and 40. All images taken on a widefield cell‐culture microscope. Scale bars, 500 μm.Whole organoid scan of a Dlxi56‐eGFP ventral: dorsal organoid fusion cryosection, day 60, immunostained for GFP marking DLXi56 reporter. Scale bar, 250 μm.Magnified (60×) dorsal regions from organoids fusions at day 60; co‐immunostaining of GFP with markers of interneuronal (GABA), GABAergic (DLX2) and mature neuronal (NeuN) identity. Scale bars, 10 μm. White arrows indicate GFP^+^ cells with positive labeling.Quantification of co‐expression of GFP with markers mentioned in 1F in dorsal and ventral regions of organoid fusions at day 60. Quantification was performed on a total of 3 organoid fusions (technical replicates) from two independent differentiations each (biological replicates), with 2 regions of interest (ROI; 10× field of view) in the dorsal and ventral regions of each fusion analyzed. Each dot represents the quantification of co‐expression for both ROI analyzed per organoid fusion. Significance values, *< 0.05, **< 0.01, ***< 0.001. Statistical analysis was performed using an unpaired two‐tailed Student’s *t*‐test. The central band displays the mean and error bars depict the SD.

**Figure EV1:**
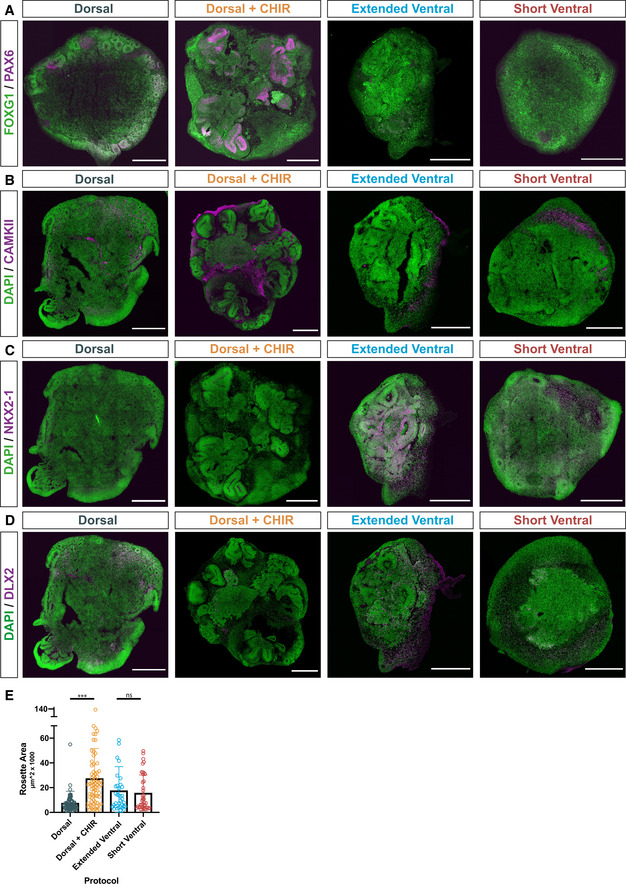
Modified protocols generate region‐specific cerebral organoids AWhole organoid scans of single organoids generated using the different protocols listed in Fig 1A. Cryosection of organoids at day 40 immunostained for markers of forebrain (FOXG1; green) and dorsal forebrain (PAX6; magenta) identity. Scale bars, 500 μm.B–DWhole organoid scans of single organoids generated using the different protocols listed in 1A. Cryosection of organoids at day 40 immunostained for DAPI (green) and an excitatory neuron marker (B; CAMKII; magenta), an MGE marker (C; NKX2‐1; magenta), or an interneuronal marker (D; DLX2; magenta). Scale bars, 500 μm.EQuantification of area (μm^2^ × 1,000) of individual SOX1^+^ rosettes within the single organoids generated using the different protocols at day 40. Quantification was performed on a total of 3–4 organoids per protocol. Each dot represents the size of one rosette within the analyzed organoids. Significance values, ***< 0.001. Statistical analysis was performed using one‐way ANOVA and post hoc Tukey’s comparison of means.

Ventral–dorsal forebrain organoid fusions (Bagley *et al*, 2017; Birey *et al*, 2017; Xiang *et al*, 2017) are a unique model system to study human interneuron migration and its regulation by neurotransmitters. We utilized a modified ventral–dorsal organoid fusion method where enhanced regional patterning was achieved by (i) a dorsal patterning treatment using a GSK3 inhibitor (CHIR) to enhance WNT signaling (Lancaster *et al*, 2017) and (ii) an extended ventral patterning treatment. We determined the effect of these modifications by comparing organoids generated using the enhanced protocols (Dorsal + CHIR (D^+^) or Extended Ventral (EV)) with tissue generated using a Dorsal (D) or Short Ventral (SV) protocol (Fig 1A, Dataset EV1). All protocols (Fig 1A) produced organoids expressing the forebrain marker FOXG1 with low expression of an optic cup marker VSX2, indicating proper telencephalic differentiation (Fig 1B). The D^+^‐protocol induced higher expression of the dorsal forebrain markers PAX6 and EMX2 compared to the D‐protocol (Fig 1B). Expression of the ventral forebrain marker NKX2‐1 was higher in the EV‐protocol compared to the SV‐protocol (Fig 1B). Most importantly, CAMKII‐expressing glutamatergic excitatory neurons were present in PAX6^+^ D^+^‐organoids (Fig EV1A and B) and DLX2‐expressing GABAergic interneurons in NKX2‐1^+^ EV organoids (Fig EV1C and D), indicating that the respective forebrain tissues correctly produce excitatory or inhibitory neurons as occurs during fetal brain development. Moreover, the rosette areas of organoids generated with the D^+^‐protocol were significantly larger (Fig EV1E) indicating the expansion of progenitor pools caused by CHIR treatment (Lancaster *et al*, 2017). These results indicate that CHIR treatment correctly promotes dorsal forebrain identity and extending the duration of ventral patterning results in stronger induction of ventral forebrain identity. Therefore, the dorsal and extended ventral protocols were used to produce dorsal–ventral cerebral organoid fusions for studying interneuron migration.

Our previous organoid fusion protocol incorporated a ubiquitously expressed fluorescent reporter to label ventral‐derived cells and track their migration (Bagley *et al*, 2017). Since this reporter may label other migratory cell populations generated in ventral regions such as OPCs (Crawford *et al*, 2016), we generated a GABAergic interneuron‐specific transgenic reporter (DLXi56‐GFP; Dimidschstein *et al*, 2016; Fig 1C). Ventral forebrain DLXi56‐GFP^+^ (GFP^+^) organoids were fused with unlabeled dorsal forebrain organoids (Fig 1D). After 40 days of differentiation, expression of GFP was observed only in ventral regions (Fig 1D). After 60 days, GFP^+^ cells were also observed in dorsal regions (Fig 1E), confirming that GFP^+^ cells migrated into dorsal cortical regions, similar to migrating interneurons during fetal brain development. Immunostaining revealed that GFP^+^ cells expressed GABAergic (GABA, DLX2) and neuronal (NeuN) markers (Fig 1F), confirming their GABAergic identity. Interestingly, only 30% of ventrally located (ventral), but 70% of dorsally located (dorsal) GFP^+^ cells expressed GABA (Fig 1G) which correlates with the increased expression of GABA in mature interneurons *in vivo* (Le Magueresse & Monyer, 2013). Conversely, the immature interneuron marker DLX2 was expressed by nearly all (˜90%) ventral GFP^+^ cells, but only by 30% of dorsal GFP^+^ cells (Fig 1G). Moreover, expression of the mature neuronal marker NeuN was significantly increased in dorsal compared to ventral GFP^+^ cells (Fig 1G), confirming that dorsal GFP^+^ cells represented mature interneurons.

**Figure EV2:**
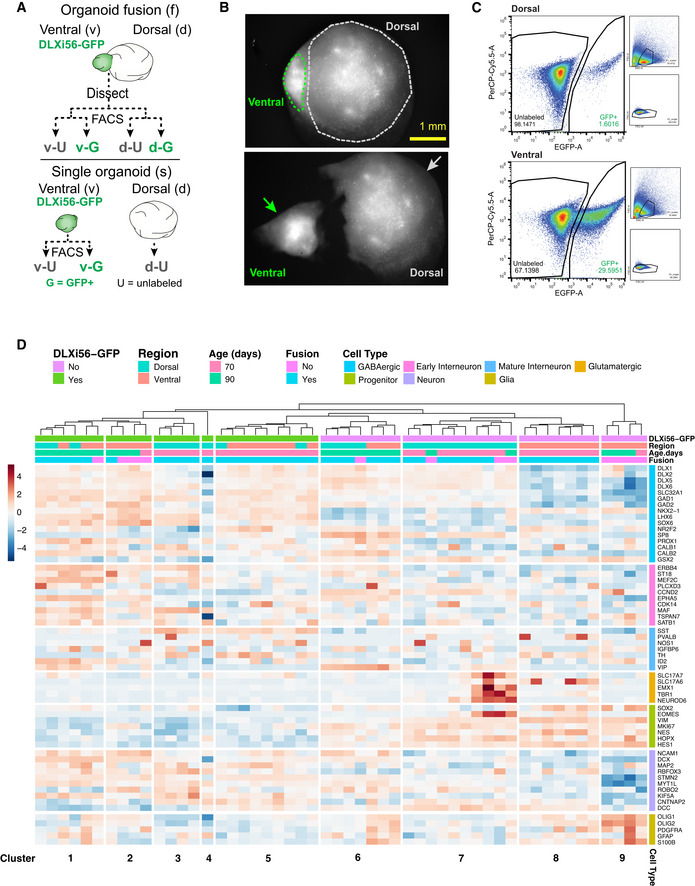
RNA sequencing reveals cellular transcriptomes in organoids Schematic representation of the experimental strategy for the RNA sequencing of cells from single and fused organoids. GFP^+^(G) and GFP‐/unlabeled(U) cells were obtained from both ventral and dorsal regions of dissected organoid fusions. G and U cells were obtained from single ventral organoids, and U cells were obtained from single dorsal organoids. In total, 7 different populations of cells were then analyzed using RNA sequencing.Representative visualization of fusion using brightfield imaging for identification of ventral and dorsal regions during manual dissection. Images show fusion before and after dissection.Flow cytometry plots for dissociated cells from either dorsal or ventral regions of dissected fusions. Cells were sorted based on their EGFP intensity (x‐axis). GFP^+^ proportion within each region is indicated within figure (dorsal—1.6%; ventral—29.59%). Across multiple dissections, proportions for GFP^+^ cells remained consistently at 1–7% for dorsal regions and 30–50% for ventral regions.Heatmap depicting the weighted gene expression (z‐score, bar indicating values from blue to red) of the different cell populations analyzed using RNA sequencing. Populations were stratified according to their GFP positivity, region they were isolated from, organoid age and whether they were isolated from a region of a fusion or a single organoid. Genes are sorted into groups relating to the cell type they relate to. Hierarchical clustering separates the individual samples into clusters which are labeled at the bottom. Source data are available online for this figure.

To further confirm generation of the correct forebrain tissue and the specificity of the DLXi56‐eGFP reporter, we performed bulk RNA sequencing on whole single D^+^ organoids, sorted GFP^+^ and GFP‐ cells from single EV organoids, and sorted GFP^+^ and GFP‐ cells from ventral and dorsal regions of dissected fusions (Fig EV2A). Dissections were performed using fluorescent illumination (Fig EV2B) and GFP^+^ and GFP‐ cells were obtained via fluorescence‐activated cell sorting (FACS; Fig EV2C). We observed that GFP^+^ cells (clusters 1–5, Fig EV2D) showed a GABAergic and neuronal transcriptomic signature, indicating the enrichment of GABAergic populations using the DLXi56‐eGFP reporter and confirming the immunohistochemistry analysis (Fig 1F–G). GFP‐ cells in ventral regions (clusters 8–9, Fig EV2D) showed progenitor and glial gene enrichment, indicating the generation of non‐GABAergic populations such as oligodendrocytes in ventral regions as well and further confirming the GABAergic specificity of the reporter. GFP‐ cells in dorsal regions (clusters 6–7, Fig EV2D) showed expression of genes associated with glutamatergic neurons and progenitors, indicating the generation of pyramidal neurons within dorsal organoids. We also observe a GABAergic signature (cluster 7, Fig EV2D) within these clusters, indicative of GABAergic cells produced in dorsal regions due to the incomplete induction of pure dorsal fate within these organoids, which is consistent with previous studies (Giandomenico *et al*, 2019).

Overall, the immunohistochemistry and RNA sequencing confirm that the D^+^ and EV protocols generate dorsal and ventral forebrain tissue, respectively. Interneurons generated in ventral regions can be labeled with the DLXi56‐eGFP reporter, and we observed that they mature according to their ventral to dorsal spatial location in dorsal–ventral forebrain organoid fusions as observed in vivo (Marín & Rubenstein, 2001). Therefore, we can use this system to determine gene expression trajectories of migrating human interneurons.

### Single‐cell profiling defines spatial axis of maturation of human cortical interneurons

**Figure 2:**
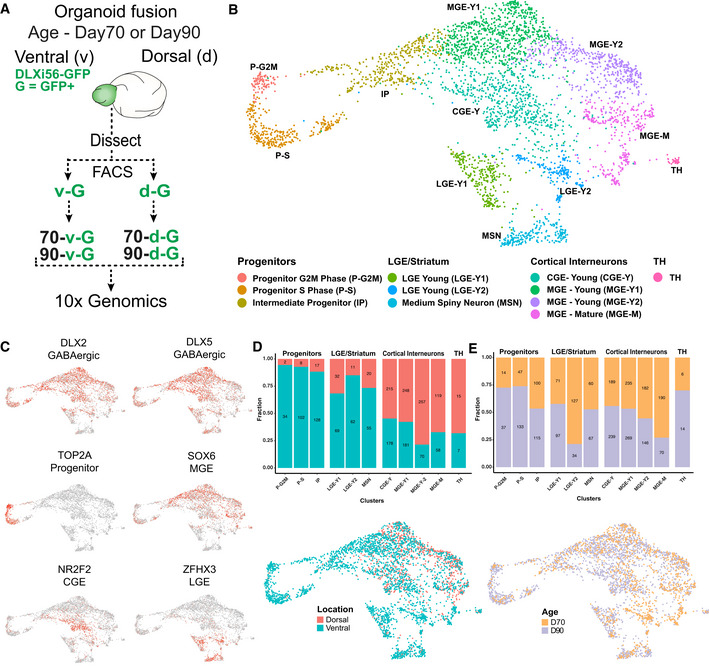
scRNAseq identifies spatial axis of maturation of human cortical interneurons Schematic overview of experimental setup for single‐cell RNA sequencing of human cortical interneurons from organoids. GFP^+^ cells from the dorsal and ventral regions of 70‐ or 90‐day‐old organoid fusions were obtained by flow cytometry and sequenced individually.Visualization of single‐cell RNA‐sequencing data from GFP^+^ cells from the different groups mentioned in 2A using UMAP after removal of stressed cells (Fig EV3A–D). Clusters are color‐coded according to the cell type.UMAPs depicting the expression of GABAergic markers *DLX2* and *DLX5*, progenitor marker *TOP2A,* MGE marker *SOX6*, CGE marker *NR2F2*, and striatal marker *ZFHX3*.Proportions (y‐axis) and numbers (within bars) of cells arising from the different regions of a fusion are listed for each cluster. Numbers are down‐sampled to 941 cells in the smallest region group for proper representation. UMAP depicting the fusion region from which the cells were isolated.Proportions (y‐axis) and numbers (within bars) of cells arising from the different fusion ages are listed for each cluster. Numbers are down‐sampled to 1,221 cells in the smallest age group for proper representation. UMAP depicting the fusion age at which the cells were isolated.

**Figure EV3:**
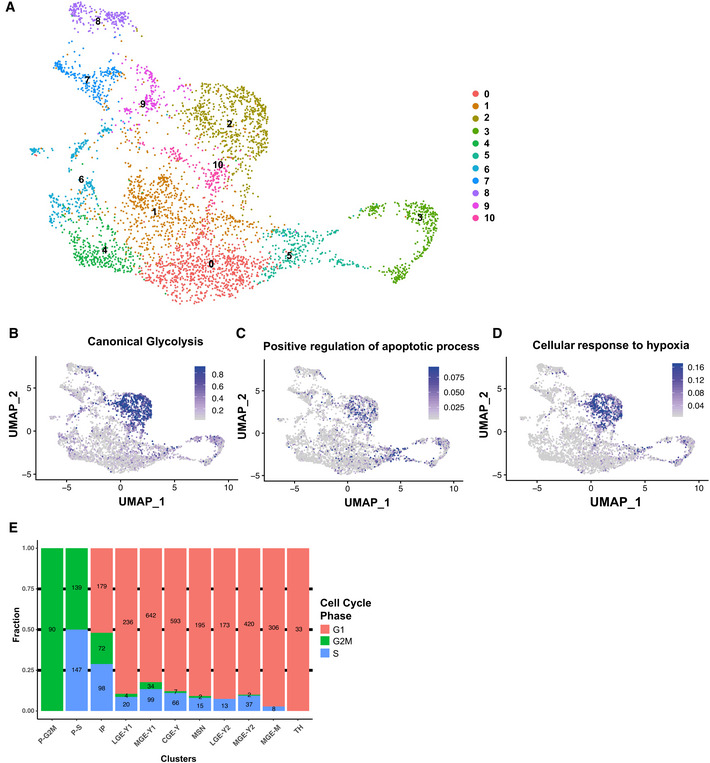
scRNAseq characterizes GABAergic cells in cerebral organoid fusions Visualization of single‐cell RNA‐sequencing data from GFP^+^ cells from the different groups mentioned in Fig 2A using UMAP and color‐coded based on the clustering.UMAP depicting the relative expression of genes linked to the GO‐term “Canonical glycolysis” (GO‐0061621).UMAP depicting the relative expression of genes linked to the GO‐term “Positive regulation of apoptotic process” (GO‐0043065).UMAP depicting the relative expression of genes linked to the GO‐term “Cellular response to hypoxia” (GO‐0071456).Proportions (y‐axis) and numbers (within bars) of cells in either G1, G2 M, or S Phase of the cell cycle are listed for each cluster after filtering and removal of stressed populations.

To specifically characterize migrating human GABAergic interneurons and analyze their neurotransmitter receptor expression, we performed single‐cell RNA sequencing (scRNAseq). We assessed the maturation status of interneurons across the spatial axis of their ventral–dorsal migration by analyzing cells from both ventral and dorsal regions of fusions. To account for a temporal maturation axis, we analyzed fusions from two different ages (day 70 and 90) (Fig 2A). 4458 GFP^+^ cells were sequenced and subsequent unsupervised clustering identified 11 cell clusters (Fig EV3A). After removal of an abnormal, stressed glycolytic population observed previously in brain organoids (Bhaduri *et al*, 2020; Fig EV3B–D), the remaining 3,635 cells were re‐clustered into 11 clusters (Fig 2B, Dataset EV2). As expected, most DLXi56‐GFP^+^ cells expressed *DLX2* and *DLX5* (Fig 2C), confirming the enrichment of GABAergic populations. We identified progenitors expressing *TOP2A*, *CCNB1*, and *NUSAP1* (Figs 2B and C, and EV3E, and Appendix Fig S1A) and intermediate progenitors expressing *VIM*, *HES6*, and *NES* (IP, Figs 2B and EV3E, and Appendix Fig S1B). Cortical interneurons are derived from two ventral forebrain progenitor subdivisions, namely the medial ganglionic eminence (MGE) and caudal ganglionic eminence (CGE), which give rise to different interneuron subtypes. The GFP^+^ population also consisted of MGE‐derived interneurons expressing *LHX6*, *SOX6* and *MEF2C*, and CGE‐derived interneurons expressing *NR2F2* and *NR2F1* (Fig 2B and C, Appendix Fig S1C and D and S2A). These results indicated that the GFP^+^ population contains all the major subtypes of cortical interneurons.

In addition to cortical interneurons, ventral forebrain progenitors also produce extracortical GABAergic populations consisting of MGE‐derived striatal interneurons and lateral ganglionic eminence (LGE)‐derived striatal projection neurons called medium‐spiny neurons (MSNs; Gokce *et al*, 2016; Miura *et al*, 2020). The GFP^+^ population contained both LGE‐derived neurons expressing *SIX3*, *TLE4,* and *ISL1* (Appendix Fig S1E and S2A) as well as *EBF1*, *TAC1*, and *FOXP1* expressing MSNs (Gokce *et al*, 2016) (MSN, Fig 2B, Appendix Figs S1F and S2A). Two groups of MGE‐derived striatal interneurons including cholinergic interneurons expressing *LHX8* (Ahmed *et al*, 2019) (Appendix Figs S1G and S2A) and TH‐positive interneurons expressing *CRABP1* and *NFIA* (TH, Fig 2B, Appendix Figs S1H and S2A; Muñoz‐Manchado *et al*, 2018) were also identified. In conclusion, the DLXi56‐GFP reporter is specific to the GABAergic lineage and labels all major human forebrain GABAergic populations, including both cortical and striatal interneurons as well as GABAergic projection neurons.

Since we observe that interneurons mature during the ventral–dorsal migration within organoid fusions, we focused on evaluating their maturation status in relation to their regional location within organoid fusions. Progenitors were predominantly located in ventral regions (˜95%; Fig 2D), corresponding to their characteristic localization in the developing ventral forebrain. Moreover, progenitors were present at both day 70 and day 90 of organoid differentiation (Fig 2E) indicating that interneurons are generated continuously within fusions. This is consistent with previous observations of proliferative cells in human ganglionic eminence regions until ˜28 weeks of gestation (˜200 days) (Bigio, 2011) and late generation and maturation of particularly CGE interneurons (Nicholas *et al*, 2013). Cortical interneurons (Fig 2B) were present in dorsal regions (Fig 2D), indicating their migration into and subsequent maturation in dorsal regions. Moreover, cortical interneurons were present in both 70‐ and 90‐day‐old fusions (Fig 2E), which confirmed that their maturation was independent of organoid age. Striatal neurons do not migrate into the cortex, but instead remain in the ventral forebrain (Marín & Rubenstein, 2001). Correspondingly, striatal neurons (Fig 2B) were located only in ventral regions of fusions (Fig 2D). We observed that the majority of LGE‐Y2 cells were observed in 70‐day‐old organoids (Fig 2D). Since striatal neurons do not migrate into the cortex and remain in ventral regions, this may indicate a temporal bias for the generation of this subpopulation which is a distinction from what we observed for cortical interneurons which exhibit a spatial but not temporal bias.

Overall, GFP^+^ cells in ventral regions were mainly interneuron progenitors and striatal neurons, while dorsal regions contained mainly migrating and mature cortical interneurons. Altogether, these results indicate that the observed dorsal–ventral distribution of cell types resembles the correct arrangement present in the fetal brain. Most importantly, the maturation status of cortical interneurons is uncorrelated with organoid fusion age and instead depends on their migration from ventral into dorsal regions. Our interest was to understand cortical interneuron migration. Therefore, we performed a focused transcriptomic reconstruction of cortical interneuron lineages to identify which neurotransmitter genes were expressed in migrating interneurons.

### Migrating human cortical interneurons express neurotransmitter receptors

**Figure 3:**
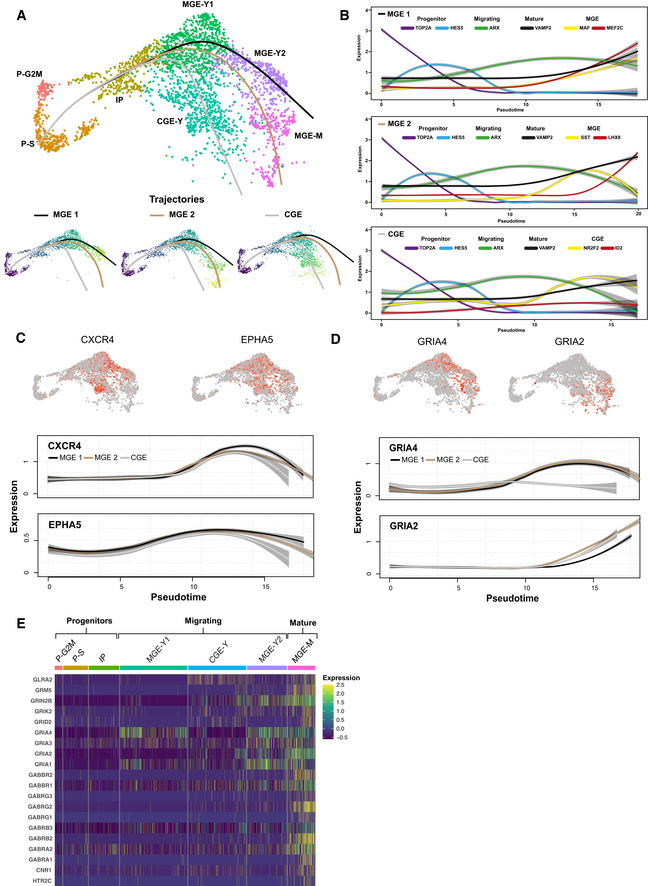
Migrating interneurons express neurotransmitter receptors UMAP depicting three pseudotime trajectories—two MGE trajectories and a CGE trajectory. Additionally, visualization of each trajectory on isolated UMAPs. After removal of the clusters relating to LGE/striatum and the TH cells, a total of 2,968 cells were used for pseudotime analysis.Visualization of the relative gene expression for characteristic genes along the pseudotime of the three trajectories. The genes visualized are color‐coded to represent their respective pseudotemporal expression.UMAPs depicting the relative expression of migration genes *CXCR4* and *EPHA5*. Visualization of the gene expression of migration genes *CXCR4* and *EPHA5* along the pseudotime axis.UMAPs depicting their relative expression of neurotransmitter genes *GRIA4* and *GRIA2* along with visualization of their gene expression along the pseudotime axis.Heatmap depicting the expression of various neurotransmitter genes for all cells across the 7 clusters analyzed. Clusters are color‐coded and labeled according to depiction in 2B. Clusters are labeled to reflect clusters with progenitors, migrating interneurons, and mature interneurons.

In order to isolate the migrating cortical interneuron populations, we performed a pseudotemporal analysis of our transcriptional dataset. We used cells from both ventral and dorsal regions to be able to track the transcriptomic maturation of cortical interneurons from their progenitor/young states, through the period of migration and eventual maturation within dorsal regions. To enable a clear identification of the trajectories of maturing cortical interneurons, we focused our analysis on GFP^+^ cells from the MGE and CGE and excluded non‐cortical striatal lineages (LGE/striatum and TH, Fig 2B). In total, 2,968 cells were used for pseudotemporal analysis. We identified three trajectories comprising two MGE (MGE‐1, MGE‐2, Fig 3A) and one CGE (CGE, Fig 3A) trajectories. Along these trajectories, we observed sequential expression of developmental genes giving rise to intermediate and mature cell types (Fig 3B). Progenitor markers such as *TOP2A* and *HES5* were expressed early followed by a marker of young, migrating interneurons (*ARX)*, and finally late expression of the synaptic marker *VAMP2* (Fig 3A and B, and Appendix Fig S1A, B, I and J). Lineage‐specific markers revealed mature and subtype‐specific cell types generated in each trajectory. We detected markers of parvalbumin (PV^+^) interneurons (*MAF* and *MEF2C)* (Mayer *et al*, 2018) in the MGE‐1 (Fig 3B and Appendix Fig S1C), and markers of both SST^+^ interneurons (*SST)* and striatal cholinergic interneurons (*LHX8)* in the MGE‐2 trajectory, indicating distinct subtype‐specific lineages of mature MGE‐derived interneurons (Fig 3B and Appendix Fig S1C and G). Increasing expression of *NR2F2* in the CGE trajectory indicated the generation of CGE‐specific interneurons (Figs 3B and 2C). Across all lineages, the same maturational progression from progenitor, to immature and then mature neurons was observed. Therefore, we reasoned that the immature interneurons could represent the migrating interneuronal population. This was confirmed by expression of genes known to be essential for interneuron migration (*CXCR4* and *EPHA5*, Fig 3C) during this intermediate portion of the pseudotime axis. This expression pattern correlated with high expression in the young (MGE‐Y1, MGE‐Y2, and CGE‐Y) populations (Fig 3C and Appendix Fig S2B), and low expression in progenitors and mature subtypes (Fig 3C and Appendix Fig S2B). Next, we analyzed the expression of neurotransmitter receptor genes across the pseudotime axis. As expected, neurotransmitter receptor expression was high in mature cortical interneurons, which mirrors synaptic marker expression (Fig 3D and E). Interestingly, migrating human cortical interneurons also expressed a diverse repertoire of neurotransmitter receptor genes (Fig 3D and E). Among them were GABA (A and B subtypes), glutamate (NMDA, AMPA, and Kainate subtypes), glycine, serotonin (HTR2C), and cannabinoid receptors (Fig 3E). Some receptor genes such as *GRIA2* were expressed in migrating interneurons but had highest expression in mature interneurons. In contrast, specific genes such as *GRIA4* had expression patterns (Fig 3D) similar to those of known regulators of migration such as *CXCR4* and *EPHA5* (Fig 3C), indicating specific upregulation in migrating interneurons. We further looked at the expression of the receptor genes in the previous (Appendix Fig S2) bulk RNA‐sequencing data and confirmed their expression in GFP^+^ cells from both ventral and dorsal GFP^+^ cells (Appendix Fig S3A). We also performed immunohistochemistry for the identified genes and observed the co‐expression of GFP with GABRA1, GRIA2/3, GRIK2, NMDAR1, HTR2C, and GLYR in dorsal fusion regions, further confirming the expression of these receptors by GFP^+^ interneurons (Appendix Fig S3B–G).

Taken together, these data suggest that a wide range of neurotransmitters may influence and regulate human interneuron migration. The transcriptomic and immunohistochemical analysis of cortical interneurons during their migration from ventral into dorsal regions of organoid fusions revealed the expression of various neurotransmitter receptor genes. This expression pattern prompted us to test whether neurotransmitter signaling, apart from its canonical role in synaptic communication in mature neurons, could also affect the migration dynamics of human interneurons.

### TrackPal enables quantification of intrinsic migration dynamics of migrating human interneurons

**Figure 4:**
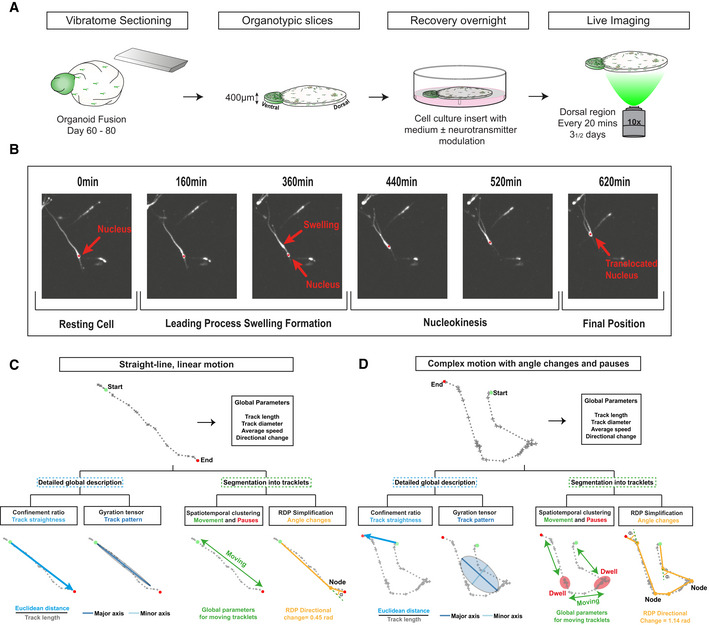
TrackPal quantifies human cortical interneuron migration Cortical interneuron migrationSchematic overview of setup for live‐imaging analysis of organoid fusions. Organoids at day 60–80 were sectioned with a vibratome to obtain 400‐µm‐thick sections. Dorsal regions of sections were then imaged every 20 min for three and a half days.Illustration of saltatory tangential migration performed by an interneuron. Nucleus is marked with a red asterisk. All images taken on a spinning disk confocal microscope at 10× magnification.Overview of different parameters calculated by TrackPal, which are used for track analysis of a track with straight‐line motion. Global parameters provide initial assessment, after which the track is analyzed by either detailed global analysis parameters or segmented into tracklets. For global analysis, the confinement ratio provides information on the track straightness and the gyration tensor is used to analyze the area covered by a cell. For track segmentation, either spatiotemporal density‐based clustering (DBSCAN) or the Ramer–Douglas–Peucker (RDP) simplification algorithm is used. Subsequently, detailed analysis is performed on the tracklets. Green and red dots mark the beginning and end of track. Yellow dots are nodes between different segments of the track in the RDP algorithm. Movement period of the cell is marked as “Moving”.Overview of different parameters calculated by TrackPal, which are used for track analysis of a track with multiple directional changes and pausing periods. Parameters are analyzed as mentioned in 4C. Green and red dots signal the beginning and end of track. Yellow dots are nodes between different segments of the track in the RDP algorithm. Note, that the movement period (green) is separated by multiple phases of dwelling (red), that are marked as “Dwell” and “Moving” respectively.

To understand the functional role of the identified neurotransmitter signaling systems (Fig 3E) in the regulation of human interneuron migration, we analyzed the effect of pharmacological perturbation of these pathways on the migratory behavior of GFP^+^ interneurons in dorsal–ventral organoid fusions. In order to screen multiple signaling pathways, we first developed an analytical pipeline for time‐lapse analysis using organoid fusion slice cultures. We recorded GFP^+^ interneurons during their migration within dorsal regions of organoid fusions over three days (Fig 4A). Qualitative analysis revealed that migrating interneurons exhibited classic saltatory migration behavior with complex leading process dynamics, as observed in previous rodent and human studies (Marín *et al*, 2010; Bagley *et al*, 2017; Birey *et al*, 2017; Xiang *et al*, 2017): During this behavior, each cell dynamically formed a proximal swelling of the leading edge (process). Subsequent nucleokinesis propelled the lagging nucleus toward these swellings, resulting in a net‐forward locomotion of the interneuron (Fig 4B and Movies [Link], [Link] and [Link], [Link]). These swellings are difficult for cell segmentation programs to distinguish from cell bodies, which is the first step in creating migration tracks for analysis. Therefore, we first employed interactive machine learning (Sommer & Straehle, 2011) to specifically segment GFP^+^ interneuron cell bodies (Appendix Fig S4A) and combined this with a semi‐automated tracking algorithm(Hand *et al*, 2009) to reconstruct the migration trajectories of individual cells. This procedure precisely captured complex trajectories including intertwining paths of multiple migrating interneurons (Movie EV3). This analytical pipeline was able to track interneurons within entire dorsal regions of organoid fusion slice cultures (Appendix Fig S4B and C, and Movies [Link], [Link], [Link], [Link]) yielding a collection of migratory tracks for individual human interneurons.

The multiphasic, saltatory dynamics of migrating interneurons (Bellion *et al*, 2005) makes an in‐depth quantitative analysis using conventional track descriptors challenging. To overcome this issue and comprehensively quantify interneuron migration, we developed a migration analysis software, Tracking Python analyzer (TrackPal) (Software EV1). It combines available track descriptor algorithms (Beltman *et al*, 2009; Mokhtari *et al*, 2013) with specifically developed track features for saltatory migration patterns into a single software package. In total, TrackPal implements 48 different track parameters (Dataset EV3) which are either based on (i) the analysis of complete tracks, or are derived (ii) after subdividing interneuron tracks into smaller, coherent parts (tracklets).

Similar to analyses used in previous studies, track parameters such as track length, speed, direction, confinement ratio (straightness), and gyration tensor characterize overall track statistics and patterns (Fig 4C and D). For track parameters targeting saltatory migration, we applied two different track partitioning schemes: spatiotemporal clustering (Ester *et al*, 1996) discriminated periods of movement (moving tracklets) from pausing (dwell states), and the Ramer–Douglas–Peucker (RDP) algorithm (Ramer, 1972) identified major directional changes within a track (Fig 4C and D). Thus, the partitioning of tracks enabled detailed descriptions of phases of motility (movement/pausing) or guidance (major direction).

Altogether, TrackPal provides a unified framework for track pre‐processing (track smoothing), track feature computation, track visualization, and MSD and VAC analysis, allowing comprehensive characterization of the saltatory interneuron migration and screening of the effect of chemical modulators on cell migration.

### Neurotransmitters differentially regulate cortical interneuron migration behavior

**Figure 5:**
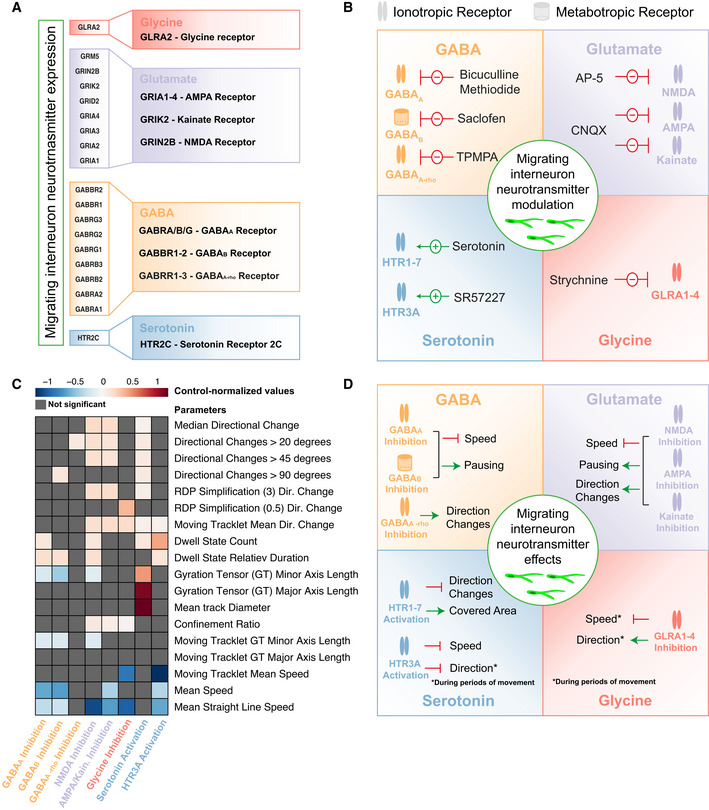
Neurotransmitters differentially regulate cortical interneuron migration Various neurotransmitter genes as identified in 3E. Genes are marked (color code) to represent the neurotransmitter receptor they encode for: Glycine (red), glutamate (purple), GABA (yellow), and serotonin (blue).Simplified schematic representation of the modulation of neurotransmitters in migrating cortical interneurons. GABA, glutamate, glycine, and serotonin transmission was modulated. Receptors and their corresponding inhibitors (red arrows) or stimulators (green arrows) are listed for each neurotransmitter system.Heatmap depicting the control‐normalized values (log_2_‐scaled) for selected parameters (from Appendix Fig S9A) for the 8 receptors analyzed in 5B. Gray values indicate non‐significant values. In total 3,979 moving cell tracks with the following number of tracks per group: control—696, bicuculline‐methiodide—1,189, Saclofen—527, TPMPA—138, AP‐5—429, CNQX—500, strychnine—98, serotonin—180, and SR57227—222, were analyzed.Simplified schematic representation of the effect of the analyzed neurotransmitters (Fig 5B) on migrating cortical interneurons. The effect of each receptor modulation is listed for each neurotransmitter system. The green arrows indicate a stimulatory while red arrows indicate inhibitory effects. Source data are available online for this figure.

Using TrackPal, we analyzed the migration dynamics of human interneurons during pharmacological perturbation of the GABAergic, glutamatergic, glycinergic, or serotonergic neurotransmitter systems (Fig 5A and B and Appendix Table S1). Since other receptors, namely the GABA A‐rho and the HTR3A receptor, have previously been implicated in regulating interneuron migration, we also included these pathways in the analysis despite low receptor gene expression. In total, ˜4,000 interneurons were individually tracked and analyzed using TrackPal. A combination of parameters (Appendix Fig S5A and B) was used to identify and exclude track artifacts (Appendix Fig S5C) caused by tissue drift during multi‐day time‐lapse recordings. The effect of each neurotransmitter pathway was assessed individually after addition of a chemical modulator for a particular receptor and compared to unperturbed control tracks.

#### Distinct GABA receptors differentially alter interneuron migration

**Figure EV4:**
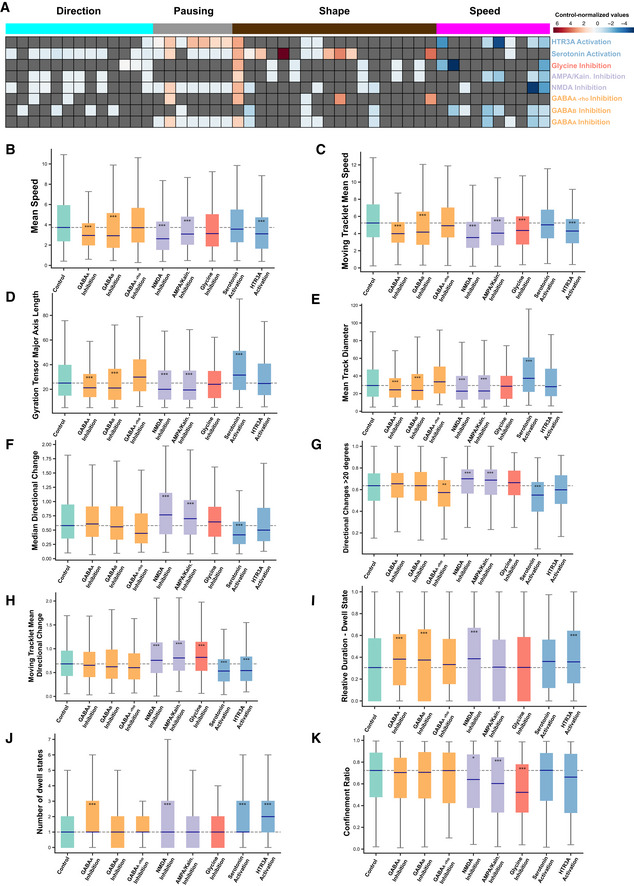
Neurotransmitters differentially regulate cortical interneuron migration AHeatmap depicting the control‐normalized values (log_2_‐scaled) for all 48 parameters calculated by TrackPal for the 8 receptors analyzed in Fig 5B. Gray values indicate non‐significant values. For visualization purposes, parameters are grouped according to their function – direction, pausing, shape, and speed.BBox plot visualizing the comparison of the mean speed for control and 8 treatment groups. The bars are colored as in Fig 5B to indicate their involvement in activation or inhibition of a neurotransmitter system: yellow bars—GABA, purple bars—glutamate, red bar—strychnine, blue bars—serotonin. All *P*‐values are calculated with a Mann–Whitney *U*‐test with Bonferroni correction. Blue central band on bars indicates the median for each group, the boxes depict values between lower and upper quartiles, the whiskers display the minimum and maximum values, and the dashed line visualizes the median for the control group for comparison. Significance values, *< 0.05, **< 0.01, ***< 0.001.C–KBox plot visualizing: (C) Mean moving tracklet speed for control and treatment groups mentioned in Fig 5B. Analysis performed as described above. (D) Major axis length of the gyration tensor for control and treatment groups mentioned in Fig 5B. Analysis performed as described above. (E) Mean track diameter for control and treatment groups mentioned in Fig 5B. Analysis performed as described above. (F) Median of directional changes for control and treatment groups mentioned in Fig 5B. Analysis performed as described above. (G) Proportion of directional changes over 20 degrees for control and treatment groups mentioned in Fig 5B. Analysis performed as described above. (H) Mean moving tracklet directional change for control and treatment groups mentioned in Fig 5B. Analysis performed as described above. (I) Relative dwell state duration for control and treatment groups mentioned in Fig 5B. Analysis performed as described above. (J) Number of dwell states for control and treatment groups mentioned in Fig 5B. Analysis performed as described above. (K) Confinement ratio for control and treatment groups mentioned in Fig 5B. Analysis performed as described above.

The major inhibitory neurotransmitter GABA acts via three different receptors (GABAA, GABAB, and GABAA‐rho), of which the GABAA and GABAB subtypes were expressed by migrating human interneurons in cerebral organoid fusions (Fig 5A). GABA is produced by GABAergic interneurons themselves and acts as an autocrine modulator (Bekkers, 2011). We observed that inhibition of the GABAA receptor by bicuculline‐methiodide (Fig 5B) reduced the motility of migrating human interneurons resulting in reduced global speed, reduced speed during periods of movement, and an increase in number and duration of pauses (Figs 5C and EV4A–C and I–J). However, this inhibition did not alter the directionality of migrating interneurons, as their average directional change (Figs 5C and EV4F) and the directional changes during periods of movement were similar to control (Figs 5C and EV4H). These results were consistent with observations in rodents, where inhibition of the ionotropic GABAA receptor reduced the motility of migrating rodent interneurons without affecting their directionality (Manent *et al*, 2005; Manent & Represa, 2007; Bortone & Polleux, 2009). On the other hand, little is known about the role of the metabotropic GABAB receptor in modulation of interneuron migration (Lopez‐Bendito, 2003). Inhibition of the GABA_B_ receptor by Saclofen (Fig 5B) mirrored the effects of GABAA inhibition, as migrating interneurons had reduced speed and prolonged pausing durations (Figs 5C and EV4A–C and I–J), while their directionality was not altered (Figs 5C and EV4F and H).

The ionotropic GABAA‐rho receptor influences layer positioning of migrating cells entering the cortical plate (CP); however, its modulatory effects on cortical interneuron migration remain unclear (Luhmann *et al*, 2015). In contrast to perturbation of GABAA and GABAB signaling, inhibition of the GABAA‐rho receptor by TPMPA (Fig 5B) did not alter the motility of interneurons (Figs 5C and EV4A–C and I–J). Instead, inhibition of GABAA‐rho resulted in a reduction in the number of directional changes (Figs 5C and EV4G).

These results revealed that GABA signaling influences both motility and direction of migrating human interneurons and that specific receptors might modulate distinct features of migration dynamics. Both the ionotropic GABAA and the metabotropic GABAB receptors appeared to regulate motility, while migration direction was controlled by a separate ionotropic GABAA‐rho receptor (Fig 5D). Thus, our data suggest that a single neurotransmitter, GABA, may differentially regulate interneuron migration by signaling through distinct receptors.

#### Glutamate regulates both motility and directionality of migrating interneurons

Migrating interneurons further express NMDA and non‐NMDA (AMPA or Kainate) receptors (Fig 5A), which mediate the signaling of the major excitatory neurotransmitter glutamate. In particular, previous studies have shown the AMPA sensitivity of MGE‐born interneurons in the developing human cortex(Mayer *et al*, 2019). In our organoid fusions, glutamate is secreted by the pyramidal neurons in dorsal tissue (Bekkers, 2011). Inhibition of NMDA receptors with AP‐5 or non‐NMDA receptors with CNQX (Fig 5B) resulted in significantly confined motion evidenced by the significantly lower speed and increased pausing (Figs 5C and EV4A–C and I–K). Inhibition of any of the glutamate receptors led to an increase in directional changes (Figs 5C and EV4E–H). These data indicate that glutamate signaling promotes the motility of migrating interneurons via the NMDA and AMPA/Kainate receptors, which confirms similar roles in rodent interneurons (Manent *et al*, 2006; Bortone & Polleux, 2009; Luhmann *et al*, 2015). However, in contrast to rodent interneurons, the results suggest that glutamate also regulates directionality of the movement of human interneurons. Overall glutamate signaling through different receptors appears to regulate both motility and guidance of migrating interneurons (Fig 5D).

#### Glycine alters interneuron migration during periods of movement

The receptor for glycine, the secondary inhibitory neurotransmitter in the brain, is also highly expressed in migrating human interneurons (Fig 5A) and glycine is abundantly present in the organoid culture medium (Lancaster *et al*, 2017). While glycinergic signaling stimulates motility of rodent interneurons(Avila *et al*, 2013), we observed that inhibition of the glycine receptor using strychnine (Fig 5B) did not affect the overall speed or direction of migrating human interneurons, indicating species‐specific differences in how glycine affects interneuron migration. Interestingly, strychnine specifically decreased speed and increased directional changes only during periods of movement (Figs 5C and EV4C and H), indicating that glycine promotes motility and guides migrating human interneurons specifically during their movement (Fig 5D).

#### Serotonin guides interneuron migration

Migrating human interneurons in organoid fusions expressed the serotonin receptor HTR2C (Fig 5A). Serotonin acts through seven major subtypes of receptors, which have divergent roles. In the developing brain, serotonin is produced in the raphe nucleus of the hindbrain, which broadly releases serotonin throughout the cortex using widespread, long‐distance projections (Charnay & Léger, 2010). Since human dorsal forebrain organoids do not contain the raphe nucleus or its projections, they do not contain endogenous serotonin signaling networks. Therefore, in contrast to the inhibitory approach used for other endogenous neurotransmitter systems, we enhanced serotonin signaling by the addition of serotonin and a specific HTR3A agonist (SR57227) (Fig 5B). The results revealed that although ectopic serotonin did not alter the motility of migrating interneurons (Figs 5C and EV4A–C), it caused fewer directional changes and larger track area (Figs 5C and EV4A, D–H). Interneurons displayed fewer directional changes during periods of movement (Fig EV4H) after HTR3A activation, while their motility was reduced which was not observed when stimulating with serotonin (Figs 5C and EV4A–C). These results indicated that serotonin may serve a specific role as a guidance cue for migrating human interneurons, since the analyzed interneurons reduced the number of directional changes while covering larger distances (Fig 5D). Furthermore, the results emphasize the possibility of species‐specific differences for serotonin‐mediated regulation of interneuron migration, because HTR3A activation did not increase interneuron motility as reported in rodents (Murthy *et al*, 2014). However, these results are consistent with recent studies highlighting human‐specific patterns of serotonin receptor expression in interneurons (Hodge *et al*, 2019).

### Cluster analysis differentiates modes of human interneuron migration

**Figure 6:**
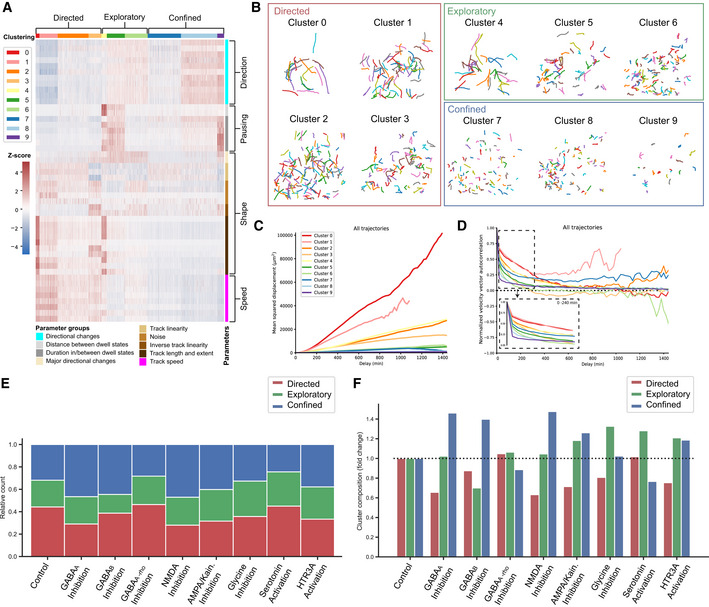
Multimodal cortical interneuron migration is modulated by neurotransmitter signaling Heatmap depicting normalized values for 48 migration parameters for all cells tracked. Parameters are grouped in 4 categories – speed, shape, pausing and direction – and 9 subgroups, which are color‐coded and described. Cell tracks are clustered into 9 different clusters based on their expression values for the different parameters. The clusters are color‐coded and numbered. Clusters are further grouped into 3 major categories – directed, exploratory and confined. The z‐score scale describes the normalized expression values. In total 3,979 moving cell tracks with the following number of tracks per group: control—696, bicuculline‐methiodide—1,189, Saclofen—527, TPMPA—138, AP‐5—429, CNQX—500, strychnine—98, serotonin—180, and SR57227—222 were analyzed.Representative visualization of cell tracks in the various clusters identified in Fig 6A. For visualization purposes, only cells from the control group are used.Mean square displacement (MSD) in μm^2^ over the delay in minutes (min) for all cell tracks in a cluster. The curves are color‐coded to denote the corresponding cluster. For visualization purposes, the MSD is shown for a time delay corresponding to 24 h of imaging.Velocity autocorrelation (VAC) over the delay in minutes (min) for all cell tracks in a cluster. The curves are color‐coded to denote the corresponding cluster. For visualization purposes, the VAC is shown for a time delay corresponding to 24 h of imaging. The dashed box depicts the VAC for each cluster for 4 h of imaging to visualize the initial decay.Stacked bar plot depicting the relative distribution of cell tracks among the three cluster groups—directed, exploratory, or confined—for control and treatment groups. Bars are color‐coded to denote the corresponding cluster group.Bar plot depicting the fold change in composition of cluster groups—directed, exploratory, or confined—of treatment groups relative to the control distribution. Bars are color‐coded to denote the corresponding cluster group. The dotted line represents the control baseline for visualization of the fold changes between the groups. Source data are available online for this figure.

**Figure EV5:**
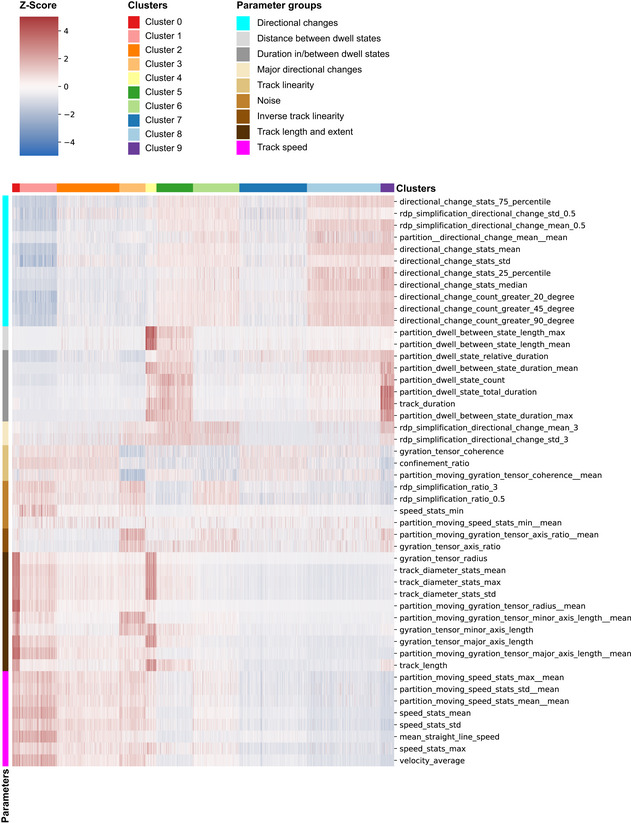
Migrating cortical interneurons display migration modes Detailed view of heatmap in Fig 6A, depicting normalized values for 48 migration parameters for all cells tracked. Parameters are grouped into 9 different subgroups, which are color‐coded and described. Cell tracks are clustered into 10 different clusters based on their expression values for the different parameters. The clusters are color‐coded and numbered. The z‐score scale describes the expression values.

Interneuron migration is a prolonged process during which cells exhibit different behaviors at different times (Hatanaka *et al*, 2016). Neurotransmitters may selectively regulate unique phases of migration. Since the interneuron population in cerebral organoid fusions consisted of GFP^+^ cells at different stages of maturation, it was plausible that they too would display a range of migratory behaviors or modes. We hypothesized that different modes of migration could be identified and characterized based on the intrinsic migratory dynamics of these individual cells. Therefore, to classify different migratory behaviors and analyze how these behaviors are regulated by various neurotransmitters, we performed a cluster analysis on the 48 parameters extracted for each individual track using TrackPal (Dataset EV3). We first performed clustering on all control cell tracks and then assigned a cluster to each cell track from the treated groups to visualize the all tracked interneurons (Fig 6A). To exclude the possibility that treatment may lead to entirely new clusters not found in control interneurons, we also clustered all cells regardless of treatment group and saw no difference between the two approaches (Appendix Fig S6). The analysis stratified the migration tracks from individual interneurons into 10 different clusters (Figs 6A and EV5, Dataset EV4) based on different categories of parameters: speed, shape, pausing, and direction (Fig 6A). Within these 10 clusters, we manually identified three different groups of clusters with similar properties and further characterized them (Fig EV5).

The first group of interneurons could be characterized by fast, straight‐line trajectories with few changes in direction (Figs 6A and B and EV5). They moved with the highest speed and fewest directional changes, indicating the straightness of their tracks (Figs 6A and EV5). The duration and frequency of pauses were lowest in these cells (clusters 0–3), confirming the high efficiency of this type of movement. Therefore, we defined this mode of migration as Directed Motion (DM). Cells which visually exhibited motion similar to DM but displayed more directional changes and/or more frequent and longer periods of pausing (Fig 6A and B) were characteristic for the second group. These cells (clusters 4–6) were characterized by high speed and large track extent but had either more frequent and increased pausing (cluster 4), both more pausing and directional changes (cluster 5) or more directional changes (cluster 6) (Fig EV5). Overall, since these cells move efficiently but also deviate off their course and pause more frequently, displaying Exploratory Motion (EM). The last group of interneurons (clusters 7–9) moved visibly more restrictedly and evinced the slowest individual speeds (Fig 6A and B). Their migratory behavior was defined by increased number and amplitudes of directional changes, longer pausing periods, and reduced speed and track extent (Fig EV5). This indicated that these cells did not cover large distances and were exhibiting Confined Motion (CM). The mean‐squared displacement (MSD; Michalet, 2010) and the velocity autocorrelation curve (VAC)(Dieterich *et al*, 2008) (Fig 6C and D, Appendix Fig S7A and B, and Dataset EV3), describe the overall movement of populations. Interneurons exhibiting DM had the largest MSD and slowest decrease in VAC, while interneurons displaying EM and CM had gradually smaller MSD and faster decrease in VAC. These results suggest that the cluster analysis captures a range of migration motion types, which corresponds to currently known modes of interneuron migration (Hatanaka *et al*, 2016).

### Neurotransmitter signaling differentially regulates individual modes of human interneuron migration

To examine how the modulation of neurotransmitter function influenced these modes of migration exhibited by interneurons, we compared the proportions of cells displaying either DM, EM, or CM across the various conditions.

Inhibition of either GABA_A_ or GABAB signaling using bicuculline or Saclofen treatment, respectively, increased the proportion of cells with CM (45–50% compared to ˜30% in control) (Fig 6E). However, inhibition of GABAA resulted in a decrease in the proportion of cells demonstrating DM (˜25% compared to ˜40% in control, Fig 6E), while inhibition of GABAB signaling reduced the proportion of cells demonstrating EM. These data suggested that GABA stimulates the directed migration of interneurons via the GABA_A_ receptor, while GABAB signaling promotes exploratory migration. In contrast, inhibition of GABAA‐rho by TPMPA treatment resulted in a proportional reduction of CM (Fig 6E and F), indicating that GABAA‐rho signaling was responsible for the mediation of CM. These results supported the previous observations of diverging roles for different GABA receptors in the modulation of interneuron migration (Fig 5D).

Inhibition of glutamatergic NMDA and non‐NMDA receptors with either AP‐5 or CNQX, respectively, increased the proportion of cells depicting CM (40–45% compared to ˜30% in control) (Fig 6E and F), while the proportion of cells displaying DM was strongly reduced (Fig 6E and F). These results indicate that glutamatergic signaling via NMDA and non‐NMDA receptors may stimulate the directed migration of interneurons, thereby serving motogenic functions similar to the effects observed for GABAergic signaling through GABA_A_ receptors.

Inhibition of glycinergic signaling by strychnine decreased the proportion of cells showing DM (Fig 6E and F). However, the proportion of cells with EM increased (˜30% compared to ˜20% in control) (Fig 6E and F). These data indicated that glycinergic signaling stimulates the directed migration of interneurons. Since glycinergic signaling only affected interneuron migration during periods of movement (Figs 5C and D, and EV4C and H), this neurotransmitter system may be auxiliary to the stimulation of directed motion by GABA and glutamate, which showed strong motogenic influence on interneuron migration modes.

Enhancing serotoninergic signaling by ectopic serotonin addition increased the proportion of cells displaying EM (˜30% compared to ˜20% in control), while the proportion of cells with CM was strongly reduced (Fig 6E and F). Stimulation of the HTR3A receptor with SR57227 also led to an increase in the proportion of cells exhibiting EM; however, in contrast to serotonin stimulation, the proportion of cells with CM was increased as well (Fig 6E and F). These results further supported the notion that serotonin serves as a guidance cue for migrating interneurons. Importantly, this regulation cannot be solely mediated by the HTR3A receptor, as isolated activation of this receptor, in contrast to serotonin addition, actually enhanced CM. The results therefore suggest that serotonin may specifically promote exploratory migration and as such is involved in proper cortical dispersion and layer allocation of migrating human interneurons.

## Discussion

In this study, we developed a specific reporter that labels the major subtypes of cortical interneurons, striatal interneurons, and striatal projection neurons, indicating a broad coverage of GABAergic populations of the forebrain. This intergenic DLX5/6 enhancer‐based reporter is not tagged to the endogenous DLX5/DLX6 gene loci; therefore, we cannot assume that it is a completely faithful representation of endogenous DLX5/DLX6 expression. However, our in‐depth characterization indicates that the reporter allows precise identification of such GABAergic cells and is therefore suitable for the analysis of cortical interneuron development and as a general GABAergic lineage reporter.

Using the reporter, we profiled cortical interneurons across their ventral to dorsal migration and observed that more mature interneurons are present in dorsal regions of organoid fusions. Moreover, we also observed that there is no reduction in the proliferative potential of organoid fusions at older ages as older fusions still contained progenitors. These data also point toward the fact that the major determinant of interneuron maturation was not the age of the organoid but rather the localization of cells in either the ventral or dorsal region. These observations raise the intriguing question whether it is the process of migration itself or changes interneurons undergo after migration has subsided, that drive their maturation.

We then coupled neurotransmitter receptor expression by migrating interneurons to functional modulation of their multimodal migration using large‐scale time‐lapse imaging of migrating interneurons and development of a migration track analysis software, TrackPal. Correct migration is a multivariate process dependent on cell‐intrinsic properties, surrounding molecular cues and interaction with other cell types, such as radial glial cells and cortical projection neurons (Lodato *et al*, 2011; Marín, 2013). Migrating interneurons hence exhibit periods of movement separated by periods of pausing, in which cells sample their surrounding environment and are guided in their migration (Bellion *et al*, 2005). Therefore, any quantitative study requires isolated analysis of these phases to be able to detect intricate details of and changes in the exhibited migration behavior. Using TrackPal, we can specifically differentiate such phases in migration of interneurons and identify subtle changes in their migratory behavior. Therefore, our analysis expands the knowledge of the migratory behavior of isolated interneurons by succinctly analyzing the various phases of interneuron migration.

Interneurons migrate from their progenitor regions into the developing cortex and proceed through different migration modes. They initially follow a directed migration in migratory streams from their progenitor zones (Walsh & Cepko, 1988; Ang *et al*, 2003). Once in the developing cortex, a complex, random‐walk migration ensues where interneurons disperse within the cortex until they reach their target regions (Tanaka *et al*, 2009). Here, migration terminates and integration into the developing circuits begins. Thus, interneuron migration follows a dynamic, multimodal process (Hatanaka *et al*, 2016). Our quantitative analysis of migration using TrackPal identified migration behavior clusters of directed, exploratory and confined cells which resemble these qualitatively described modes of migration *in vivo*. Directed cells are fast and exhibit limited direction changes, reminiscent of the stream migration described for newly generated cells performing initial tangential migration (Ang *et al*, 2003). Exploratory cells show a combination of speed and directional changes, similar to the multidirectional tangential behavior observed in the marginal zone (MZ) of developing rodent cortices (Tanaka *et al*, 2006, 2009). Lastly, confined cells reflect the migration behavior of interneurons that begin to integrate into cortical circuits, with limited motility and increased directional changes. However, in vivo migration behavior of interneurons is qualitatively defined based on the location of cells relative to anatomical brain structures and layers. Cerebral organoid anatomy is not stereotyped, and therefore identifying migration modes as performed *in vivo* is not possible based on the localization of interneurons within a particular region of the dorsal or ventral organoids. Our analysis seems to overcome this limitation through quantification and clustering of single‐cell migration tracks based solely on their intrinsic migration dynamics. The fact that TrackPal classifies interneuron migration modes which resemble qualitatively described modes *in vivo*, despite not having the anatomical landmarks which allow this qualitative description, suggests that migration dynamics alone can be used to identify migration modes. Therefore, multimodal migration can now be studied i*n vitro* using this approach. As the synthesis and expression levels of different signaling cues acting on migrating interneurons are strictly regulated (Marín, 2013), they may act on specific modes of migration. By pharmacologically perturbing neurotransmitter receptors, we are hence able to discern during which mode of migration different external signaling pathways function. GABA, glutamate, and glycine may regulate tangential migration (Directed), serotonin may act as a guidance cue for multidirectional tangential migration (Exploratory) and GABA via the GABAA‐rho receptor may stimulate the integration of interneurons into circuits (Confined). It is important to note that maturing interneurons may also reduce their migration and thus exhibit confined motion. We cannot exclude the possibility that neurotransmitter modulation may lead to such changes in the maturity status of organoids and cause changes in their behavior. However, considering the short duration of the modulation this possibility is unlikely. In summary, the analyzed neurotransmitters are collectively involved in the complex regulation of cortical interneuron migration. However, it is important to note the different mechanisms guiding tangentially migrating cells in the developing human brain. Here, we apply a drug to an entire tissue and readout the collective effect. Therefore, we cannot account for changes in the cellular environment of the migrating interneurons and cannot discern whether the effect is solely the result of manipulation of receptor signaling on the cells that we monitor. It is possible that changes to receptor signaling in other cells such as pyramidal neurons may produce a secondary effect on migrating interneurons. Thus, organoid fusions and our migration analysis assay provide an ideal platform for future studies of the interaction between migrating interneurons and their cellular environment.

Here, we present a focused analysis on migrating cortical interneurons using our tracking analysis software TrackPal. However, migratory cells are integral in many key processes across organ systems and species. We developed TrackPal as open‐source software in Python—the most popular language in data science—to unify existing track analysis algorithms with track partitioning to create a simple and modular track analysis tool. Applying this approach to other cell types and tissues can help expand our understanding of the role and regulation of migration in organogenesis.

## Materials and Methods

### Stem cell culture

Feeder‐free human ES cell line (H9; WA09 from WiCell) was cultured on hESC‐qualified Matrigel (Corning, cat. #354277)‐coated plates (Eppendorf, cat. #0030720016) with Essential8 (E8) medium (Thermo Fisher, cat. #A1517001) supplemented with BSA. Cells, when confluent, were passaged by incubation in 1 mM EDTA (PanReac AppliChem, cat. #A1103) in sterile D‐PBS without calcium and magnesium (Thermo Fisher, cat. #14190136) for 5 min at 37°C and subsequent transfer at a 1:6 ratio to a Matrigel‐coated plate with E8 medium. Cells were routinely checked for mycoplasma and karyotyped.

### Cloning and generation of reporter lines

For the generation of an interneuron‐specific reporter cell line, a reporter construct was inserted into the AAVS1 safe harbor locus in hESCs using TALEN technology, as described previously (Hockemeyer *et al*, 2011). To specifically label interneurons, we designed a human version of the mouse Dlx5/6 enhancer (mDlx), which had been shown to have interneuron specificity in murine and human hPSCs (Dimidschstein *et al*, 2016). We inserted the human Dlxi56 enhancer, driven by a minimal promoter, into the AAVS1 donor vector we had used previously using eGFP to monitor expression (Bagley *et al*, 2017). The following expression construct was inserted: 2xCHS4‐Dlxi56‐HBBminP‐Intron‐eGFP‐WPRE‐SV40‐2xCHS4. Sequence files and construct plasmid DNA can be obtained from the corresponding author upon request.

Nucleofection, selection of clones with correct insertion, and quality control were performed as described previously (Bagley *et al*, 2017). Cell clones with correctly targeted insertions were archived by freezing with Cell Banker 2 solution (Amsbio, cat. #11891).

### Culture of cerebral organoid fusions

Cerebral organoid fusions (Fusions) were generated with a modified protocol, expanding upon previous organoid generation protocols from our laboratory (Lancaster *et al*, 2013, 2017; Dataset EV1).

Briefly, cells at around 75% confluency were treated with 1× Accutase solution (Sigma‐Aldrich, cat. #A6964) for 5 min at 37°C and resuspended in embryoid body medium (EB medium): E8 medium supplemented with Revitacell™ Supplement (Thermo Fisher Scientific, cat. #A2644501) for cell counting. For both dorsal and ventral forebrain embryoid body (EB) generation, 9,000 cells suspended in 150 μl of EB medium were added to each well of a 96‐well low‐attachment U‐bottom plate (Corning, cat. #COR7007). On day 3, EB medium was replaced with E8 medium for dorsal forebrain EBs (dorsal EBs) and neural induction medium (NI medium; Lancaster *et al*, 2017) for ventral forebrain EBs (ventral EBs). From day 5–11, dorsal EBs were cultured in NI medium, while ventral EBs received drug‐patterning treatment and were cultured in NI medium supplemented with 100 nM SAG (Merck‐Millipore, cat. #US1566660) and 2.5 μM IWP2 (Sigma‐Aldrich, cat. #IO536). In contrast to previous protocols, EBs were not embedded in Matrigel (Corning, cat. #3524234) at this point, but transferred to dishes with Matrigel‐supplemented media. On day 11, 10‐cm^2^ cell‐culture dishes were coated with anti‐adherent Pluronic® F‐127 (Sigma‐Aldrich, cat. #P2443) solution and EBs were transferred to coated dishes containing either 10 ml NI medium supplemented with 1% Matrigel (dorsal) or 10 ml NI Medium supplemented with 1% Matrigel, 100 nM SAG, and 2.5 μM IWP2 (extended ventral). On day 13, medium was changed to either improved differentiation medium—A supplemented with 1% Matrigel (Imp‐A + MG) and 3 μM CHIR99021 (Merck, cat. #361571) (dorsal) or Imp‐A + MG, 100 nM SAG and 2.5 μM IWP2 (extended ventral; Lancaster *et al*, 2017). Dorsal organoids received an additional CHIR99021 (3 μM) boost on Day 15 (no media change). On day 17, both dorsal and ventral EBs received Imp‐A + MG and dishes were transferred to an orbital shaker at 57 rpm.

Dorsal and ventral organoids were placed in wells of a low‐attachment 96‐well plate containing Imp‐A + MG on day 20, to allow pre‐fusion before embedding. As organoids are already fairly large at this stage, pre‐fusion limits manual positioning and enables more efficient embedding on day 21. For embedding, pre‐fused organoids were gently placed in a droplet of Matrigel and pushed together with a 10 μl pipette tip, if needed. The prolonged duration of ventral forebrain induction in our extended ventral protocol may lead to even smaller sizes for ventral organoids than has been noted previously (Sloan *et al*, 2018) (Xiang *et al*, 2017). CHIR treatment leads to the expansion of progenitor pools and subsequently the cortical plate (Lancaster *et al*, 2017) and thus causes the large size of the dorsal organoids, resulting in unequal fusions. Fusions were placed in improved differentiation medium + (Imp + A) (Lancaster *et al*, 2017) and transferred to an orbital shaker at 57 rpm on day 25–26. After embedding, organoids were fed with Imp + A every 3–4 days.

### Histological and immunohistochemical analysis

Organoids, when suitable for analysis, were washed 3× in PBS and fixed in 4% PFA (Sigma‐Aldrich, cat. #441244) for 1 h at RT or at 4°C overnight. Post‐fixation, tissue were washed 3× in PBS again, after which they were transferred to a 30% sucrose in PBS at 4°C overnight. Thereafter, tissue was incubated in a 50:50 mixture of 30% sucrose (Sigma‐Aldrich, cat. #84097) and OCT (Sakura, cat. #4583) for 2 h at RT, before being transferred to a cryomold. Once in cryomold, excessive sucrose‐OCT mixture was removed, cryomold was filled with OCT and frozen on dry ice. 20‐μm slices of frozen tissue were obtained on Superfrost Ultraplus slides (Thermo Fisher, car. #10417002) using a Cryostar NX70 cryostat (Thermo Fisher, cat. #957000H). Tissue was stored at −20°C after drying overnight.

Immunofluorescence was performed on slides of sliced organoid tissue directly. Slices were surrounded by hydrophobic PAP‐pen (Sigma‐Aldrich, cat. #Z377821) and allowed to dry. Slides were washed 1× in PBS and then post‐fixed with 4% PFA for 15 min. After washing 2× with PBS, remaining 4% PFA was hydrolyzed with sodium borohydride (Sigma‐Aldrich, cat. #452882) for 5 min. Slides were washed 1× PBS and permeabilization and blocking of tissue was performed for 1 h at RT with 5% BSA and 0.3% Tween‐20 in PBS. Thereafter, primary antibodies were added to staining solution (5% BSA and 0.1% Tween‐20 in PBS) at desired concentrations and slides were incubated with prepared solutions at 4°C overnight. Slides were washed 3× in PBST (0.1% Tween‐20 in PBS), secondary antibodies were added to staining solution at 1:500, and slides were incubated with prepared solution for 1 h at RT. DAPI solution (2 μg/ml) was added for 5–10 min, and slides were washed 2× in PBST. Before mounting, slides were washed 1× in PBS and then mounted using DAKO mounting agent (Agilent Pathology solutions, cat. #S3023). Slides were stored at 4°C for imaging and −20°C for long‐term storage.

All primary and secondary antibody information is listed in Appendix Tables S2 and S3, respectively.

### Imaging and microscopy of fixed tissue

Cell‐culture imaging of live tissue was performed on a widefield microscope (Zeiss Axio Vert A1, Zeiss GmbH) with a Zeiss Axiocam ERc 5s camera (Zeiss GmbH), using Zeiss Plan‐Neofluar 2.5 × 0.085 and Zeiss LD A‐Plan 10 × 0.25 Ph1 objectives (Zeiss GmbH). ImageJ was used to merge fluorescent and brightfield images.

Confocal imaging of fixed tissue was performed on a Zeiss LSM800 Axio Imager with either a 20 × 0.8 plan‐apochromat or a 25 × 0.8 LD LCI pan‐apochromat multi‐immersion objective. XY scanning stage was used for tile scans. 405 nm (5 mW), 488 nm (10 mW), 561 nm (10 mW), and 639 nm (5 mW) lasers along with SP470, SP545, LP575, LP665, and SP620 wavelength filters were used for recording. All post‐recording image adjustments and quantifications were done in Fiji.

### Quantification of histological and immunohistochemical Images

Quantification of marker expression of Dlxi56‐eGFP^+^ cells was done using tissue sections imaged with a 20× objective on the Zeiss LSM800 microscope. Marker expression of GFP^+^ cells was quantified in two regions of interest (ROIs) in both the ventral and the dorsal regions of each fusion section. Two different sections of each organoid fusion were quantified. The percentages were then averaged across five organoids from two independent differentiations. All quantifications were done manually using the “Cell Counter” plugin on Fiji.

Rosette area quantification was performed on entire stitched organoid sections of single organoids grown using the different protocols in Fig 1A. The area was determined by manual selection using the “polygon” selection tool and area measurement using the “Analyze” function in Fiji.

### Cerebral organoid fusion slice culture and drug treatment

Organoid fusions at 60–80 days of age were sectioned using a vibratome (Leica VT1200, Leica Biosystems) to obtain slice cultures for live imaging of migrating interneurons.

Organoid fusions were embedded in 4% low‐melt agarose (Biozym, cat. #850080) and sectioned in ice‐cold HBSS (Thermo Fisher, cat. #14025092) supplemented with 0.5% glucose to obtain 400‐μm‐thick sections. Sections were immediately placed on Millicell cell‐culture inserts (Merck, cat. #PICM0RG50) in 6‐well plates (Eppendorf, cat. #0030720016) containing Imp + A differentiation medium and cultured overnight.

For time‐lapse imaging with drug treatment, slice cultures were obtained two days before imaging and placed on cell‐culture inserts as described above. Slices were pre‐treated with either fresh medium (control) or medium containing different drugs one day before imaging. Information and concentrations of all drugs used in the live‐imaging experiments are listed in Appendix Table S1. The drug concentrations were not determined using titration experiments, but were based on previous analyses in human interneurons and other cells with these drugs. After overnight pre‐treatment, slices were lifted off of cell‐culture inserts and placed in wells of a coverglass‐bottom 24‐well μ‐Plate (Ibidi, cat. #82406) containing fresh medium supplemented with the corresponding drug treatments. Slices were immobilized with a custom‐made weight attached to a protective net.

### Live imaging of slice culture

Live imaging was performed using either the Olympus IX3 Series (IX83, Olympus Corp.) or the Visiscope (Visitron Systems GmbH) spinning disk confocal microscope systems, equipped with incubation chambers. Images were acquired with a 488 nm laser and the corresponding 525/50 wavelength filters using either a 10×/0.4 UPLS‐Apo (Olympus IX83) or a 10×/0.45 CFI Plan APO lambda (Visiscope) objective.

The entire dorsal regions of each fusion were captured by scanning complete tile regions, which were stitched automatically (Olympus IX83‐acquired images) or manually with the Grid/Collection stitching plugin in Fiji (Visiscope‐acquired images; Preibisch *et al*, 2009). Slices (2–6 per condition) were imaged for a total duration of 84 h with an image acquisition every 20 min. 300‐μm z‐stacks with a step size of 5μm were obtained for each slice to account for the entire depth of the slice and any morphological tissue changes occurring over the course of imaging.

### Image processing and cell tracking

Maximum intensity z‐projection, using the “z‐projection” plugin in Fiji, was performed for all stitched images to obtain z‐projected time stacks for each recording. The “StackReg” plugin was used to correct for drift and drift‐corrected time stacks were subsequently used for further analysis.

The open‐source image classification software Ilastik was used to segment cell bodies of GFP^+^ migrating cell bodies with its “Pixel Classification” function (Sommer & Straehle, 2011; Berg *et al*, 2019). The random forest pixel classifier was trained on a control time stack to separate cell bodies from processes and background. Using the “Batch Processing” tool, all subsequent movies were automatically segmented with the same trained classifier, providing binary probability maps for each time stack. The binary probability maps and unsegmented/raw time stacks were then merged to form multi‐channel time stacks, which were then used for the cell body tracking. The image analysis software Imaris (Bitplane, Oxford Instruments) was used for cell body tracking as its high efficiency in tracking cell migration has been well reported (Mitchell *et al*, 2020). Using the “Spots” function, segmented cell bodies in the binary probability maps were recognized as spots and their movement over time was captured with the “Autoregressive Motion” algorithm, creating tracks for the migration of each cell body. By overlaying the generated tracks over the raw time stacks, each cell could be visualized. Erroneous tracks created by drifting particles or non‐moving cells were manually removed. Using the “Record” button, mp4 format videos of migrating cells with and without overlaid tracks were generated (Movies [Link], [Link], [Link], [Link], [Link], [Link]). Information on the x‐y positions of all spots of each track was exported as comma separate values and used for further analysis of track characteristics.

### Analysis of interneuron migration

Cell tracks were analyzed with custom‐written Python (ver. 3.6.4) routines based on the TrackPal (version 0.2.0; on github: https://github.com/sommerc/trackpal; Software EV1), pandas (version 1.0.4; McKinney, 2010), scipy (version 1.4.1; Virtanen *et al*, 2020) and scikit‐learn (version 0.21.1; Pedregosa *et al*, 2011) libraries.

#### Feature computation and filtering

First, the comma separated text files containing all tracked cells were read using the trackpal *read.imaris()* function as pandas *DataFrames*. Tracks shorter than 12 frames (240 min) were excluded, yielding a total number of 4,298 tracks with the following number of tracks per group: control—754, bicuculline‐methiodide—1,213, Saclofen—589, TPMPA—145, AP‐5—468, CNQX—577, strychnine—112, serotonin—197, and SR57227—243.

The *trackpal.feature* module was used to compute the following track feature classes: track_length, track_duration, track_diameter_stats, directional_change_stats, directional_change_count, speed_stats, velocity_average, gyration_tensor, partition (with t_scale = 1.1 and ɛ = 3.3), confinement_ratio, mean_straight_line_speed, rdp_simplification (for ɛ = 0.5 and ɛ = 3 µm). The resulting features descriptors from each feature class were concatenated yielding in total 48 feature descriptors per cell track (Appendix Table S2).

Using the features *track_diameter_max* and *gyration_tensor_major_axis_length,* the cell tracks were filtered to exclude non‐moving cell tracks. Thresholds for non‐moving cell tracks were set to 15 µm for *track_diameter_max* and 5 µm for *gyration_tensor_major_axis_length* by visual inspection of tracks (Appendix Fig S3). In total, 319 cell tracks were excluded, yielding 3,979 moving cell tracks with the following number of tracks per group: control—696, bicuculline‐methiodide—1189, Saclofen—527, TPMPA—138, AP‐5—429, CNQX—500, strychnine—98, serotonin—180, and SR57227—222.

#### Clustering of control interneurons

The 48 feature descriptors of the 696 control interneuron cell tracks were normalized to have zero mean and unit variance (Z‐score). Then principal component analysis (scikit‐learn) was performed on the control interneurons, where the number of principal components was chosen to yield at least 95% of explained variance resulting in 18 principal components. The principal components were clustered into *K* = 10 groups using K‐means clustering method (scikit‐learn) with 20 random initializations. The number of cluster K was chosen based on the elbow heuristic. After clustering the control interneurons, all interneuron cell tracks were normalized, transformed into principal component space and assigned a cluster with closest cluster center from the K‐means clustering. For simpler subsequent visualization, the cluster assignment numbers were reordered.

Hierarchical cluster analysis (scipy) was applied to the 48 feature descriptors using Ward's minimum variance method (Ward, 1963). Flat clusters were formed using the *maxclust* method to yield L = 9 parameter clusters. The “super clustering” (Appendix Fig S8) was performed in an unbiased clustering, as the highly aberrant nature of cluster 4 with high parameter variance (high values for speed and shape but also pausing) did not enable the differentiation of minute differences between clusters 5–9 in biased hierarchical k‐means clustering (Appendix Fig S8).

For the shift in proportions of clusters after neurotransmitter modulation (treatment groups), no statistical analysis was performed. These ratios are shifts in the fractions (or compositions) of the cluster modes and are computed on the basis of all the data (hence no repetitions for statistical analysis).

#### Mean‐squared displacement and velocity autocorrelation curves

For the mean‐squared displacement (MSD) and velocity autocorrelation curves (VAC), the trackpal modules *trackpal.msd* and *trackpal.velocity* were used.

### RT‐PCR analysis

Organoids at 30–40 days of age were collected in RNAse free Microfuge tubes (Ambion, cat. #AM12450) for RNA extraction. RNA extraction was performed using a magnetic‐bead based RNA purification kit (Molecular Biology Services, Vienna BioCenter). 1 μg of extracted RNA was used for cDNA synthesis using the Superscript III reverse transcriptase (Thermo Fisher, cat. #18080044) according to manufacturer protocols. SybrGreen GoTaq qPCR master mix (Promega, cat. #A6002) was used for the RT‐PCR on a 384‐well RT‐PCR detection machine (Bio‐Rad, cat. #CFX384). The protocol used was as follows: (I) 95°C for 3 min (II) 95°C for 10s (III) 62°C for 10s (IV) 72°C for 40s (V) go to II (40 cycles) (VI) 95°C for 1 min (VII) 50°C for 10 s. Δ*C*_t_ values of gene expression were then calculated by using TBP as a reference gene. Expression values (2^−Δ^ *^C^* ^t^)were calculated relative to TBP in Microsoft Excel. All primers used in the study are listed in Appendix Table S4.

Analysis was performed on 5–6 organoids from multiple independent differentiations for the control (7 differentiations), dorsal (7 differentiations), extended ventral (9 differentiations), and short ventral (5 differentiations) protocols.

### RNA sequencing

RNA sequencing analysis was performed on cells obtained from single dorsal organoids generated from WT H9 hESCs, single ventral organoids generated from H9 hESCs containing the interneuron‐specific Dlxi56‐eGFP construct and dissected ventral and dorsal regions of fusions of these organoids. GFP^+^ cells in the single ventral organoids, dissected ventral, and dissected dorsal regions of fusions were obtained via flow cytometry. The seven unique groups we analyzed were therefore (I) GFP‐ cells—single dorsal organoids, (II) GFP^+^ cells—single ventral organoids, (III) GFP‐ cells—single ventral organoids, (IV) GFP^+^ cells—dissected ventral regions of fusions (V) GFP‐ cells—dissected ventral regions of fusions, (VI) GFP^+^ cells—dissected dorsal regions of fusions, (VII) GFP‐ cells—dissected dorsal regions of fusions. In total, 32 single organoids and 34 fusions were used to obtain 52 samples for the RNA‐sequencing analysis.

For dissection, fusions were placed in a wax‐coated dissection dish filled with Imp + A differentiation medium. A widefield stereomicroscope (Zeiss SteREO Discovery V12) with fluorescent illumination was used to visualize Dlxi56‐eGFP signal. Using two sharp tweezers (Dumont, cat. #0203‐54‐PO), fusions were dissected along the boundary of the dorsal and ventral regions.

For dissociation, pools of single organoids or dissected regions of organoid fusions were collected at the desired ages in gentleMACS™ dissociator C tubes (Miltenyi, cat. #130093237). Single‐cell suspensions were achieved by enzymatic dissociation with 2 ml of a 1:1 mixture of Accutase (Sigma‐Aldrich, cat. #A6964) and trypsin (Thermo Fisher, cat. #15090046) using the 37C_NTDK_1 program on a gentleMACS™ dissociator (Miltenyi, cat. #130093235). Suspensions were then diluted by addition of 9 volumes of ice‐cold DMEM‐F12 (Thermo Fisher, cat. #11330057) and centrifuged at 1,000 *g* for 5 min at 4°C. Supernatants were then removed, and cell pellets were resuspended in 300 μl PBS^−/−^ supplemented with 2% BSA and 2 mM EDTA and passed through a 35‐μm cell strainer. Cells were sorted using a fluorescent cell sorter (Sony Biotechnology, SH800S) into 1.5‐ml Eppendorf tubes.

RNA extraction was performed simultaneously for all 52 samples in a 96‐well KingFisher™ deepwell plate (Thermo Fisher, cat. #95040450) using a magnetic‐bead based RNA purification kit (Molecular Biology Services, Vienna BioCenter) and the KingFisher™ Flex Purification System (Thermo Fisher, cat. #24074431). RNA quality control and concentration measurements were performed on the 5400 Fragment Analyzer™ (Agilent, cat. #M5312AA). 100 pg of RNA per sample was used for cDNA library preparation using the SmartSeq‐2 protocol (Illumina) and sequenced on a HiSeq V4 sequencing lane (Illumina).

### RNA‐sequencing data analysis

Adapters were clipped with trimgalore (v0.5.0) (Krueger F, Trim Galore, https://github.com/FelixKrueger/TrimGalore) (Martin, 2011). Abundant sequences (rRNA, mitochondrion, phiX, adapter, polyA/C) were removed with bowtie2 (v2.3.4.1) (Langmead & Salzberg, 2012). Cleaned reads were aligned against the genome (GRCh38) with STAR (v2.6.0c) (Dobin *et al*, 2013). Reads were counted toward their corresponding gene (Ensembl 94) with featureCounts (v1.6.2) (Liao *et al*, 2014). Differentially expressed genes were detected with DESeq2 (v1.18.1) (Love *et al*, 2014). Coverage tracks were created with deepTools (normalize BPM; v3.0.2) (Ramírez *et al*, 2016). Two technical replicates (two sequencing lane runs) were combined using samtools (htslib.org).

### Single‐cell RNA sequencing

Ten organoid fusions at day 70 and 90 each were first dissected. Fusions were placed in a wax‐coated dissection dish filled with Imp + A differentiation medium. A widefield stereomicroscope (Zeiss SteREO Discovery V12) with fluorescent illumination was used to visualize Dlxi56‐eGFP signal. Using two sharp tweezers (Dumont, cat. #0203‐54‐PO), fusions were dissected along the boundary of the dorsal and ventral regions.

Dissected regions were washed 2× with PBS^−/−^ and then collected in 2‐ml Eppendorf tubes containing 1 ml of a 1:1 mixture of Accutase and Trypsin. Tissue was dissociated by a 30‐min incubation at 37°C at 800 rpm shaking speed on a ThermoMixer® (Eppendorf, cat. #5384000012). Single‐cell suspensions were diluted by addition of 9 volumes of cold DMEM F/12 and centrifuged at 1,000 *g* for 5 min at 4°C. Supernatants were then removed, and cell pellets of dissected regions at the same age were resuspended and pooled in 600μl PBS^−/−^ supplemented with 2% BSA and 2 mM STA to obtain 4 samples for flow cytometry: (I) Dorsal regions—day 70, (II) ventral regions—day 70, (III) dorsal regions—day 90, (II) ventral regions—day 70. GFP^+^ cells from all samples were sorted on a FACSAria III sorter (BD Biosciences) and collected in 1.5‐ml Eppendorf tubes. After centrifugation at 1,000 g for 3 min at 4°C, cells were resuspended in 20 μl ice‐cold PBS^−/−^ supplemented with 0.04% BSA, counted and 16,000 cells were loaded onto a chromium single‐cell 3’ B Chip and processed with the chromium controller, generating Gel Bead Emulsions (GEMs) for cDNA library preparation. Libraries were generated with the 10× chromium single‐cell 3’ GEM, Library & gel Bead Kit v3 (10× Genomics, cat. #PN‐1000075). Libraries were pooled and sequenced on a NovaSeq S1 flow cell (Illumina) with 2.6–3.2 billion paired‐end reads.

### Single‐cell RNA‐sequencing data analysis

We first aligned reads to GRCh38 human reference genome with Cell Ranger 3.1 (10× Genomics) using pre‐mRNA gene models and default parameters to produce the cell‐by‐gene, Unique Molecular Identifier (UMI) count matrix. UMI counts were then analyzed using the Seurat R package v.3. We filtered for high‐quality cells based on the number of genes detected [min. 900; max. 6000] and the fraction of mitochondrial reads [min. 1%; max. 15%] and ribosomal reads [min. 5%; max. 40%]. As described previously (Kanton *et al*, 2019), we excluded clusters with “glycolysis” identity based on GO enrichment of cluster‐specific marker genes. Thereafter, expression matrices of high‐quality cells were normalized ("LogNormalize") and scaled to a total expression of 10K UMI for each cell. Regression of variables at this step did not improve clustering results; hence, no variables were regressed and removed.

### Data integration and batch correction

Variable genes were identified by Seurat's FindVariableFeatures implementation (“FastLogVMR”). Next, we aligned and merged the four sequencing libraries by Seurat's canonical correlation analysis or CCA (dimensions: 50; Butler *et al*, 2018) using the intersection variable genes across datasets.

### Clustering and Marker Gene identification

Next, principal components were calculated on the variable genes, and the first 50 components were then used to calculate UMAP coordinates.

For clustering, we used Seurat's implementation of kNN/Louvain clustering. Therein, we first calculate the k‐nearest‐neighbor (kNN) graph of cells in PCA‐space (dimensions:50). Next, each edge (between two cells) is weighted by the fraction of overlap between the two cells' k‐nearest neighbors (also known as Jaccard similarity). Louvain clustering on this graph identified clusters of cells. Differentially expressed genes were identified by Wilcoxon test and filtered for *P*‐values below 0.001, and fold change larger than 2. Genes were ranked on a score combining the *P*‐value and log‐fold change (−log10(*P*)*logFC) and are provided (Appendix Table S1). All parameters of the analysis are defined as in "Parameters.iNM.R" in the GitHub repository (vertesy/interneuronmigration).

### Pseudotime analysis

For pseudotime analysis, we used Slingshot (Street *et al*, 2018). Essentially, it constructs a minimum spanning tree (MST) of cluster centers in a reduced‐dimensionality space (2D UMAP in our case) to find the global lineage structure, including branching points. We provided the starting (0, dividing cells) and terminal clusters (5, 8, 9). Based on known marker genes ZFHX3, SIX3, ISL1, FOXP1, and EBF1, we observed that striatal lineages had no corresponding progenitor populations; therefore, we excluded these clusters (3,6,7) and the smallest cluster of TH cortical interneurons (10) and focused the pseudotemporal analysis on the MGE and CGE developmental lineages. After removal of the clusters relating to LGE/striatum and the TH cells, a total of 2968 cells were used for pseudotemporal analysis.

### Statistical analysis

Graphs were generated and statistical analysis was performed on R Studio, Python, and Prism (version 8, GraphPad). Samples were tested for normality before analysis. Analysis of RT‐PCR (Fig 1B) results for the different groups was performed using one‐way ANOVA and post hoc Tukey’s comparison of means. For quantification of marker co‐expression of DLX‐i56 GFP^+^ cells in dorsal and ventral regions (Fig 1G), an unpaired two‐tailed Student’s t‐test was performed. For quantification of rosette sizes of the organoids grown from the different protocols (Fig EV1E), statistical analysis was performed using one‐way ANOVA and post hoc Tukey’s comparison of means. For analysis for the 48 parameters of the tracking analysis (Figs 5C and EV4), first a Kruskal–Wallis test was performed to determine the variance between the different groups. Then, using non‐parametric Mann−Whitney U‐test with Bonferroni correction to correct for type 1 errors, statistical significances were examined. All results are noted in the corresponding figures or their legends. No statistical methods were used to pre‐determine sample size, which was estimated based on previous experience with similar setups. Experiments were not randomized. Investigators were blinded for immunohistochemistry analysis and not blinded for live‐imaging analysis.

## Author contributions

SB, JAB, and JAK conceived the project, designed experiments, and wrote the manuscript. SB performed experiments and analyzed data. JAB analyzed data and supervised experiments. CS programmed the tracking analysis software and analyzed tracking data. AV performed the analysis of scRNAseq data. VK performed immunostaining experiments and quantitative analysis. SNW performed tissue culture experiments and immunostaining experiments. JL‐S generated the DLXi56‐GFP reporter cell line. JAB and JAK supervised the project.

## Supporting information



Appendix

Expanded View Figures PDF

Dataset EV1

Dataset EV2

Dataset EV3

Dataset EV4

Movie EV1

Movie EV2

Movie EV3

Movie EV4

Movie EV5

Movie EV6

Software EV1

Source Data for Expanded View and Appendix

Source Data for Figure 5

Source Data for Figures 5 and 6

## Data Availability

The single‐cell RNA‐sequencing data have been uploaded to Gene Expression Omnibus (GEO) under reference number GSE161550 (http://www.ncbi.nlm.nih.gov/geo/query/acc.cgi?acc=GSE161550). The RNA‐sequencing data have been uploaded to Gene Expression Omnibus (GEO) under reference number GSE182614 (http://www.ncbi.nlm.nih.gov/geo/query/acc.cgi?acc=GSE182614). The code for analysis can be accessed on Github: https://github.com/vertesy/InterNeuronMigration. The custom‐written Python TrackPal library (version 1.2.0) is openly available on Github: https://github.com/sommerc/trackpal.
