# Supplementary material for: Neurotransmitter signaling regulates distinct phases of multimodal human interneuron migration
Source: EMBO J. 2021 Oct 18;40(23):e108714. doi: 10.15252/embj.2021108714 (PMC8634123; doi:10.15252/embj.2021108714)
Supplement: Supplementary file 1 — Appendix [file EMBJ-40-e108714-s003.pdf]

Appendix to

Neurotransmitter signaling regulates distinct phases of multimodal human interneuron migration

Sunanjay Bajaj[1,2#], Joshua A. Bagley[1,3,#], Christoph Sommer[4], Abel Vertesy[1], Sakurako Wong[1], Veronica Krenn[1], Julie Lévi-Strauss[1], Juergen A. Knoblich[1, 5,*]

The appendix contains the following supplementary information:

Appendix Figures S1-S8
Appendix Tables S1-S4

Appendix Figure Legends

Appendix Figure S1 – Marker expression identifies diverse GABAergic cell types

UMAPs depicting the relative expression of:

A. Markers of cycling progenitors
B. Markers of intermediate progenitors.
C. Genes associated with the medial ganglionic eminence (MGE).
D. Genes associated with the caudal ganglionic eminence (CGE).
E. Genes associated with the lateral ganglionic eminence (LGE).
F. Markers of striatal projection neurons.
G. Markers of striatal interneurons.
H. Early markers of TH+ striatal interneurons.
I. Markers of young, migrating interneurons.
J. Markers of synaptic transmission.

Appendix Figure S2 – scRNAseq distinguishes migrating human cortical interneurons

A. Heatmap depicting the expression of differentially expressed (DE) genes for all cells across the 11 clusters identified in Figure 2B. Clusters are color-coded and labeled according to depiction in Figure 2B.
B. Heatmap depicting the expression of selected migration genes for all cells across the 11 clusters identified in Figure 2B. Clusters are color-coded and labeled according to depiction in Figure 2B. Clusters are labeled as progenitors, striatal, migrating and mature to reflect the cell types.

Appendix Figure S3 – Neurotransmitter receptors are expressed by GABAergic interneurons

A. Heatmap depicting the weighted expression of selected neurotransmitter receptor genes for GFP+ cells from ventral and dorsal regions of fusion from the RNA sequencing depicted in Figure EV2. Populations were stratified according to their GFP-positivity, region they were isolated from and organoid age. Genes are sorted into groups relating to the neurotransmitter receptor they relate to.

B.-G **.** Magnified (63x) dorsal regions from organoids fusions at day 60; Co-immunostaining of GFP with GABRA1, GRIA2/3, GRIK2, NMDAR1, HTR2C and GLYR. Scale bars, 5µm.

Appendix Figure S4 – Precise, large-scale tracking of migrating cortical interneurons
A. Overview of image processing steps for tracking of migrating interneurons. Cell nuclei are segmented in Ilastik, which allows for proper spot recognition in Imaris. Recognized spots are then tracked over time. Scale bars; 25µm.
B. Representative overview of an organoid fusion section as captured by the live-imaging analysis. Dashed blue lines mark the ventral region of the fusion, while the dashed yellow lines outline the dorsal regions. Migrating interneurons are only tracked in the dorsal region. Scale bars, 100µm.
C. Magnified view of the region marked in Figure 4D after tracking analysis. Migration tracks for the cells are visible, with the duration of each track represented by the color-coding seen in the time scale bar. Scale bars, 100µm.

Appendix Figure S5 – TrackPal identifies and removes cell-track artifacts
A. Scatter plot depicting the distribution of all cell tracks based on their expression value for the major axis length of the gyration tensor over the maximal track diameter. Blue box represents the magnified view depicted in Appendix Figure S5B.
B. Magnified view of a sub-set of cell tracks with low values for the major axis length of the gyration tensor and the maximal track diameter. Cell tracks with low-levels were identified (red box) and excluded from further analysis.
C. Visualization of all tracks identified in Appendix Figure S5B and excluded from further analysis. Scale bars, 20µm.

Appendix Figure S6 – TrackPal provides robust analysis of single cell tracking data
Principal component analysis (PCA) comparison of the assignment of clusters based on the cell tracks for all cells and cell tracks for control cells only. The comparison is shown for the first 5 principal components.

Appendix Figure S7 – Migration modes have characteristic migratory properties
A. Mean square displacement (MSD) in μm^2 over the delay in minutes (min) for all cell tracks in each cluster. The standard error of the mean (SEM) is shown as a shaded (gray) region. For visualization purposes, the MSD is shown for a time delay corresponding to 24 hours of imaging.
B. Velocity auto-correlation (VAC) over the delay in minutes (min) for all cell tracks in a cluster. The standard error of the mean (SEM) is shown as a shaded (gray) region. For visualization purposes, the VAC is shown for a time delay corresponding to 24 hours of imaging.

Appendix Figure S8 – TrackPal provides robust analysis of single cell tracking data
Comparison of supervised and unsupervised super-clustering of the 10 clusters identified by clustering in Figure 6A using the heatmap in Figure EV5. The super-clusters are color coded to represent the directed, exploratory and confined migratory patterns.

Appendix Table Legends

Appendix Table S1

Detailed descriptions of all drugs used for neurotransmitter signaling perturbation in live-imaging analysis.

Appendix Table S2

List of primary antibodies used in this study.

Appendix Table S3

List of secondary antibodies used in this study.

Appendix Table S4

List of primers used for RT-PCR analysis in this study.

AFS1

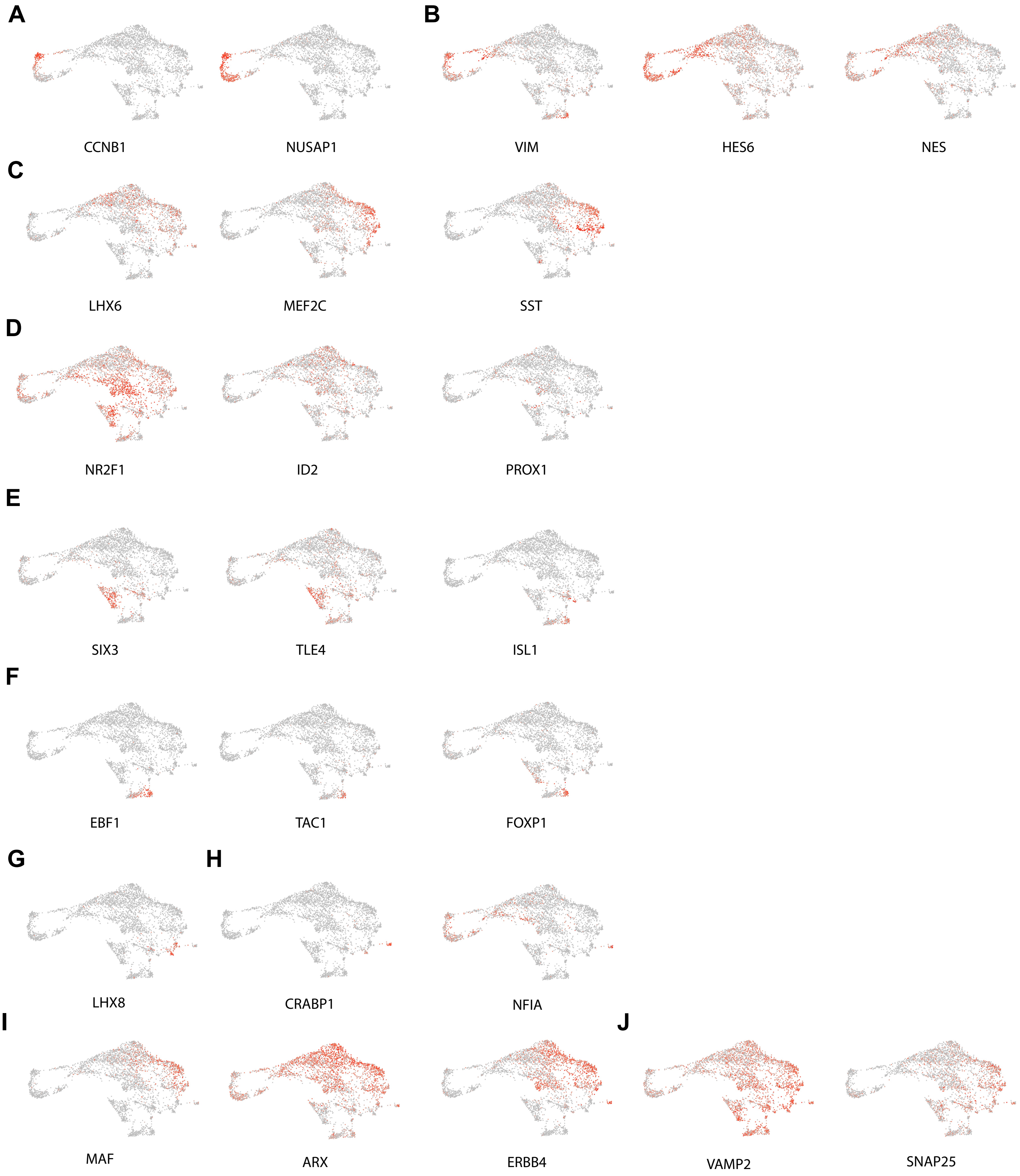
A
CCNB1
NUSAP1
B
VIM
HES6
NES
C
LHX6
MEF2C
SST
D
NR2F1
ID2
PROX1
E
SIX3
TLE4
ISL1
F
EBF1
TAC1
FOXP1
G
H
LHX8
CRABP1
NFIA
I
MAF
ARX
ERBB4
J
VAMP2
SNAP25

AFS2

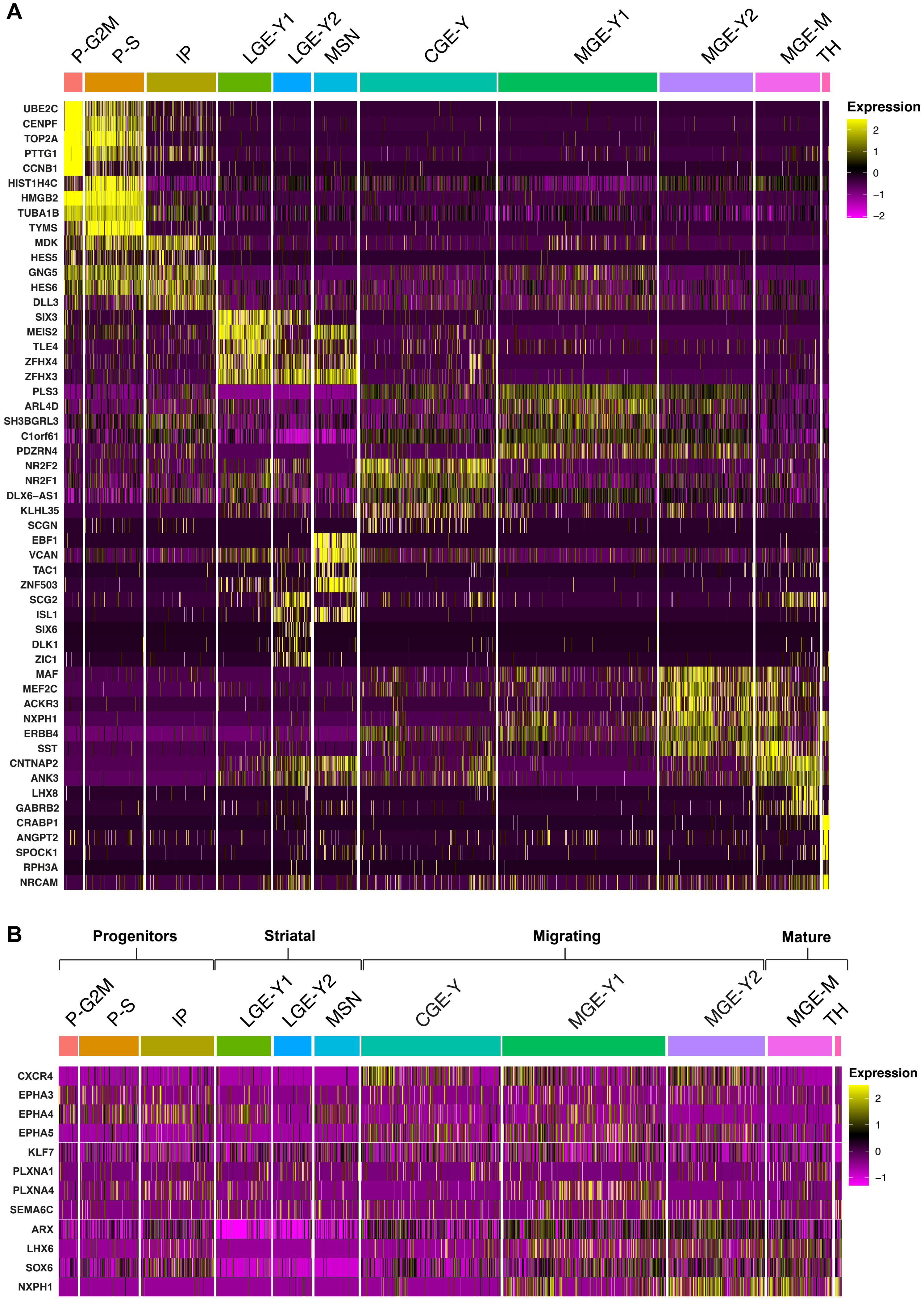
A
P-G2M
P-S
IP
LGE-Y1
LGE-Y2
MSN
CGE-Y
MGE-Y1
MGE-Y2
MGE-M
TH
UBE2C
CENPF
TOP2A
PTTG1
CCNB1
HIST1H4C
HMGB2
TUBA1B
TYMS
MDK
HES5
GNG5
HES6
DLL3
SIX3
MEIS2
TLE4
ZFHX4
ZFHX3
PLS3
ARL4D
SH3BGRL3
C1orf61
PDZRN4
NR2F2
NR2F1
DLX6−AS1
KLHL35
SCGN
EBF1
VCAN
TAC1
ZNF503
SCG2
ISL1
SIX6
DLK1
ZIC1
MAF
MEF2C
ACKR3
NXPH1
ERBB4
SST
CNTNAP2
ANK3
LHX8
GABRB2
CRABP1
ANGPT2
SPOCK1
RPH3A
NRCAM
Expression
2
1
0
−1
−2
B
Progenitors
Striatal
Migrating
Mature
P-G2M
P-S
IP
LGE-Y1
LGE-Y2
MSN
CGE-Y
MGE-Y1
MGE-Y2
MGE-M
TH
CXCR4
EPHA3
EPHA4
EPHA5
KLF7
PLXNA1
PLXNA4
SEMA6C
ARX
LHX6
SOX6
NXPH1
Expression
2
1
0
−1

AFS3

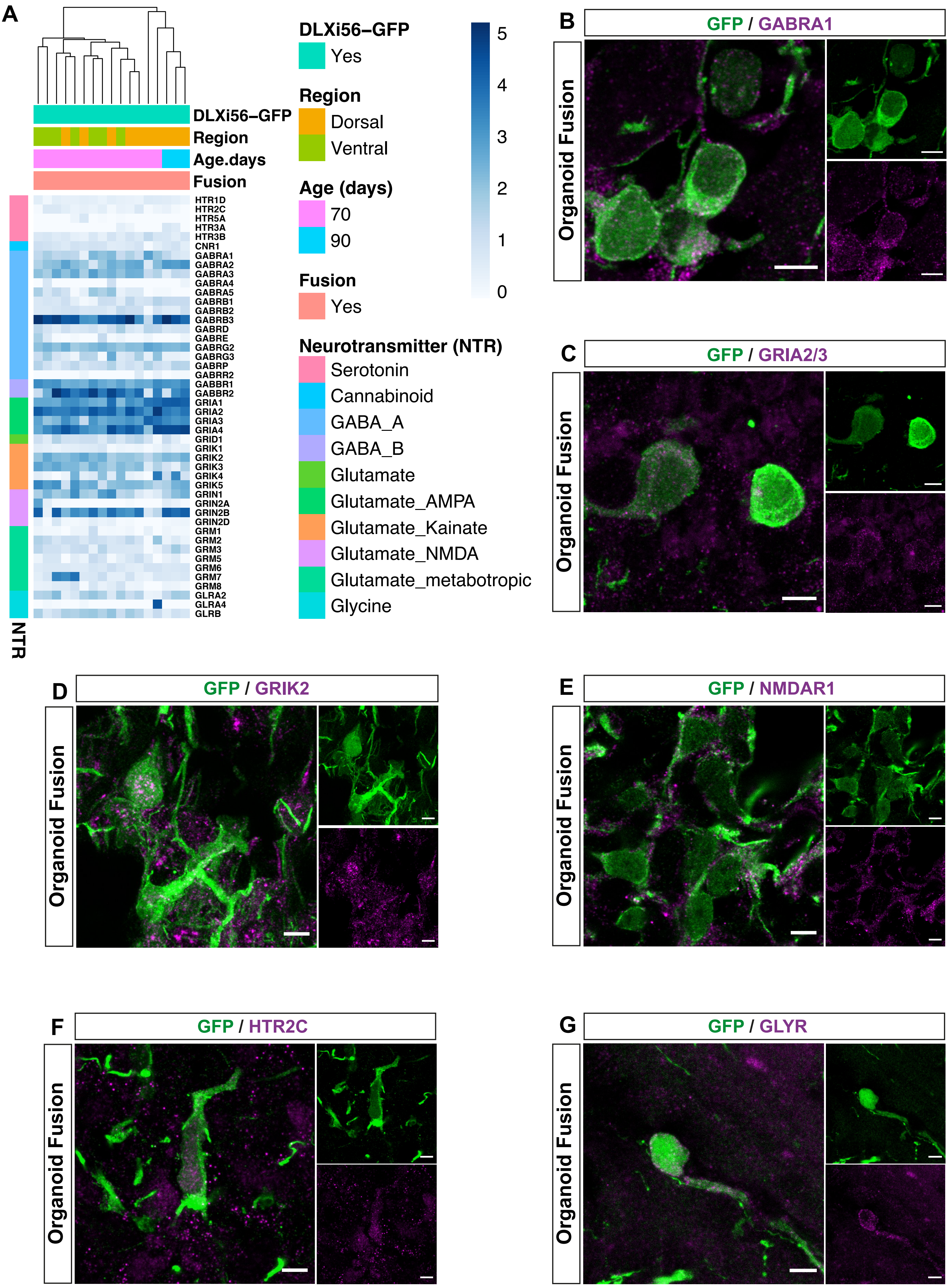
A
DLXi56–GFP
Region
Age.days
Fusion
HTR1D
HTR2C
HTR5A
HTR3A
HTR3B
CNR1
GABRA1
GABRA2
GABRA3
GABRA4
GABRA5
GABRB1
GABRB2
GABRB3
GABRD
GABRE
GABRG2
GABRG3
GABRP
GABRR2
GABBR1
GABBR2
GRIA1
GRIA2
GRIA3
GRIA4
GRID1
GRIK1
GRIK2
GRIK3
GRIK4
GRIK5
GRIN1
GRIN2A
GRIN2B
GRIN2D
GRM1
GRM2
GRM3
GRM5
GRM6
GRM7
GRM8
GLRA2
GLRA4
GLRB
NTR
DLXi56–GFP
Yes
Region
Dorsal
Ventral
Age (days)
70
90
Fusion
Yes
Neurotransmitter (NTR)
Serotonin
Cannabinoid
GABA_A
GABA_B
Glutamate
Glutamate_AMPA
Glutamate_Kainate
Glutamate_NMDA
Glutamate_metabotropic
Glycine
5
4
3
2
1
0
B
GFP / GABRA1
Organoid Fusion
C
GFP / GRIA2/3
Organoid Fusion
D
GFP / GRIK2
Organoid Fusion
E
GFP / NMDAR1
Organoid Fusion
F
GFP / HTR2C
Organoid Fusion
G
GFP / GLYR
Organoid Fusion

AFS4

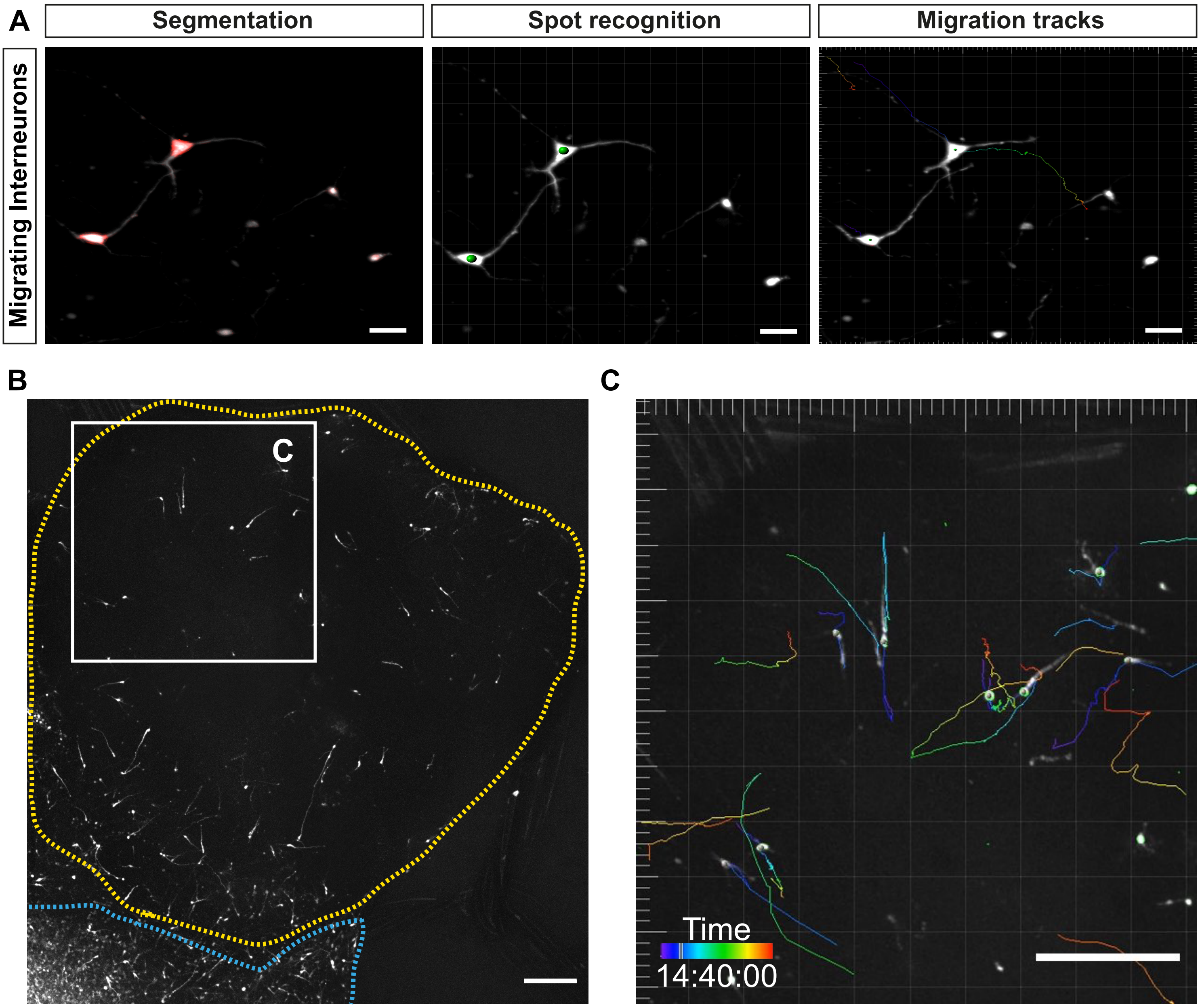
A
Segmentation
Spot recognition
Migration tracks
Migrating Interneurons
B
C
C
Time
14:40:00

AFS5

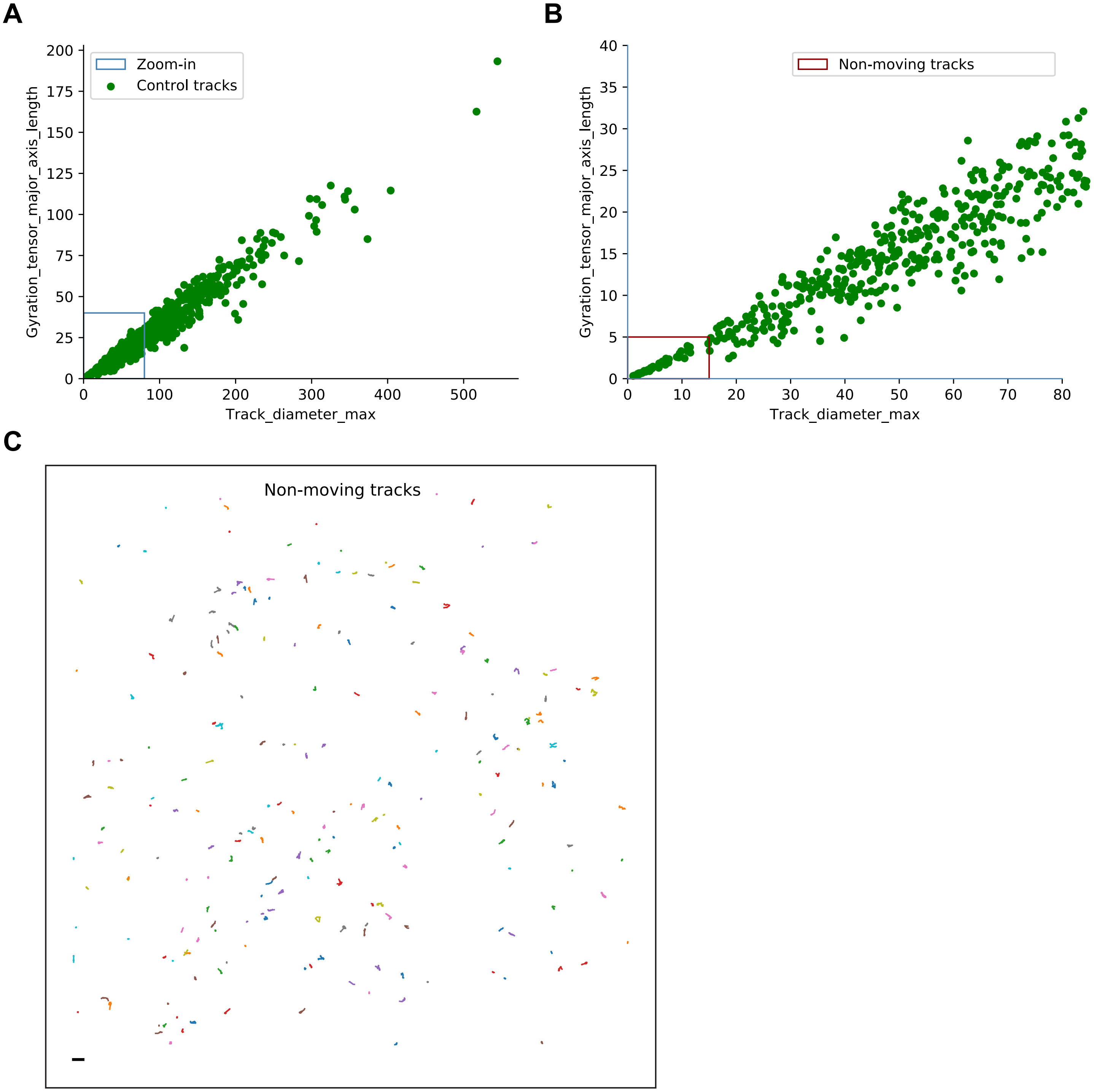

A
Zoom-in
Control tracks
Gyration_tensor_major_axis_length
Track_diameter_max
0
25
50
75
100
125
150
175
200
0
100
200
300
400
500
B
Non-moving tracks
Gyration_tensor_major_axis_length
Track_diameter_max
0
5
10
15
20
25
30
35
40
0
10
20
30
40
50
60
70
80
C
Non-moving tracks

AFS6

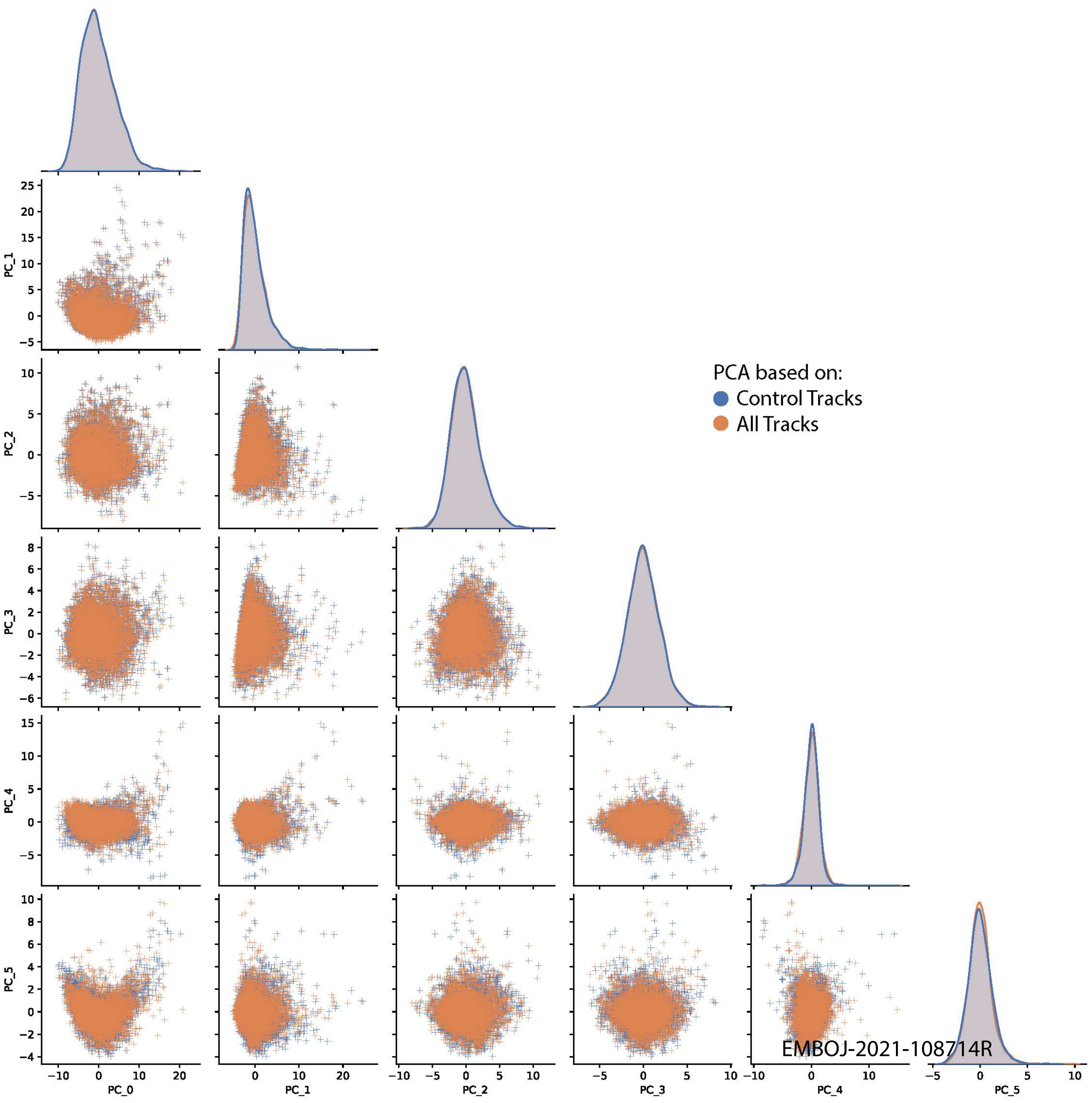

EMBOJ-2021-108714R

AFS7

A

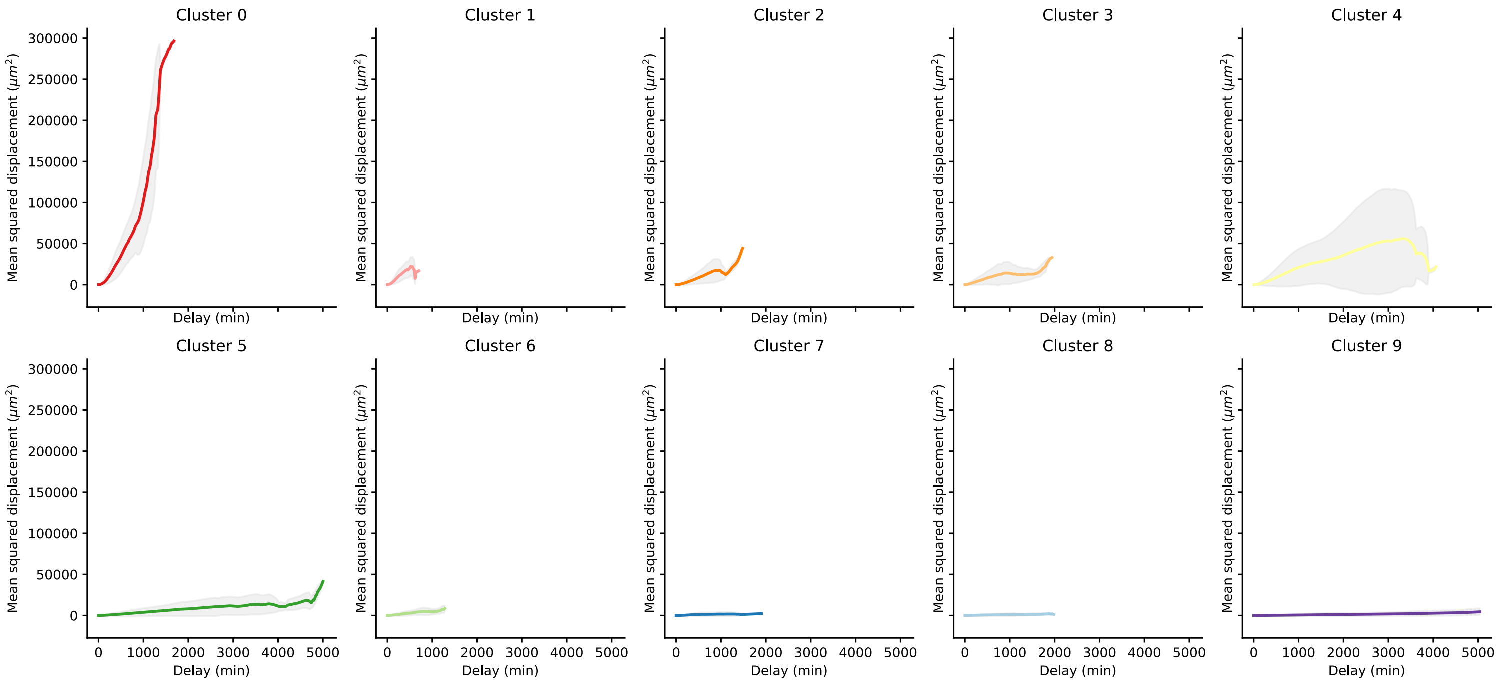

Cluster 0
Cluster 1
Cluster 2
Cluster 3
Cluster 4
Cluster 5
Cluster 6
Cluster 7
Cluster 8
Cluster 9
Mean squared displacement (μm^2)
Delay (min)
300000
250000
200000
150000
100000
50000
0
1000
2000
3000
4000
5000

B

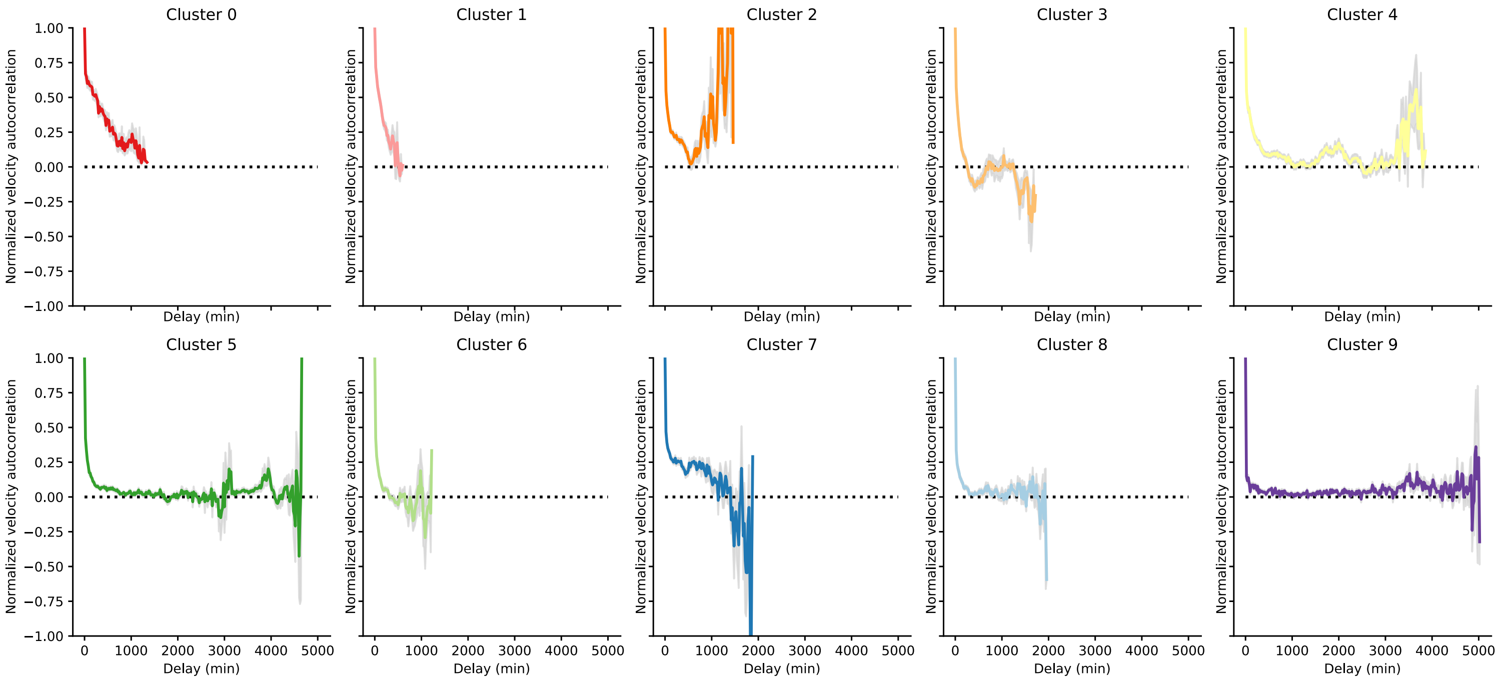

Cluster 0
Cluster 1
Cluster 2
Cluster 3
Cluster 4
Cluster 5
Cluster 6
Cluster 7
Cluster 8
Cluster 9
Normalized velocity autocorrelation
Delay (min)
1.00
0.75
0.50
0.25
0.00
−0.25
−0.50
−0.75
−1.00
0
1000
2000
3000
4000
5000

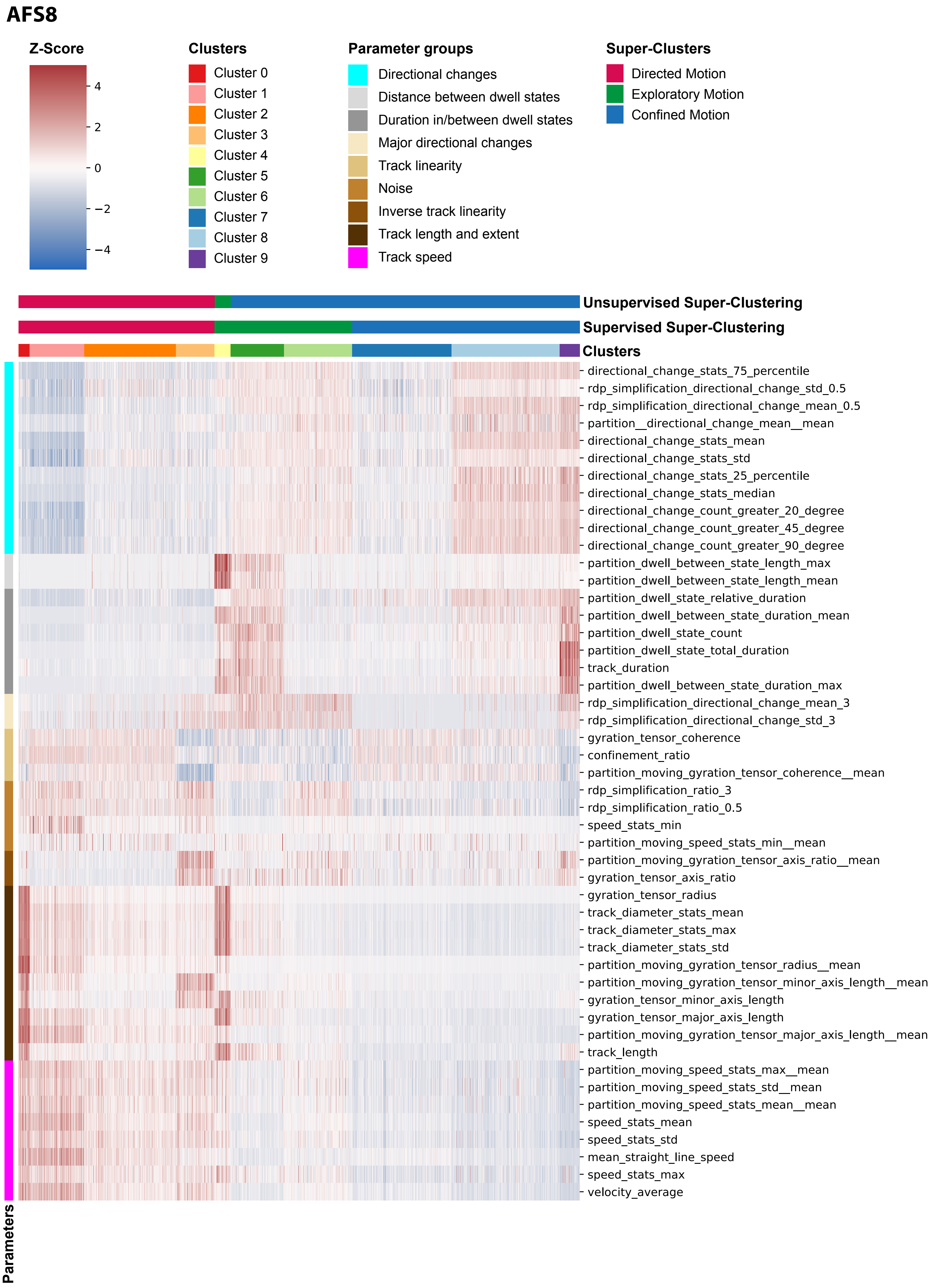
AFS8
Z-Score
4
2
0
−2
−4
Clusters
Cluster 0
Cluster 1
Cluster 2
Cluster 3
Cluster 4
Cluster 5
Cluster 6
Cluster 7
Cluster 8
Cluster 9
Parameter groups
Directional changes
Distance between dwell states
Duration in/between dwell states
Major directional changes
Track linearity
Noise
Inverse track linearity
Track length and extent
Track speed
Super-Clusters
Directed Motion
Exploratory Motion
Confined Motion
Unsupervised Super-Clustering
Supervised Super-Clustering
Clusters
directional_change_stats_75_percentile
rdp_simplification_directional_change_std_0.5
rdp_simplification_directional_change_mean_0.5
partition__directional_change_mean__mean
directional_change_stats_mean
directional_change_stats_std
directional_change_stats_25_percentile
directional_change_stats_median
directional_change_count_greater_20_degree
directional_change_count_greater_45_degree
directional_change_count_greater_90_degree
partition_dwell_between_state_length_max
partition_dwell_between_state_length_mean
partition_dwell_state_relative_duration
partition_dwell_between_state_duration_mean
partition_dwell_state_count
partition_dwell_state_total_duration
track_duration
partition_dwell_between_state_duration_max
rdp_simplification_directional_change_mean_3
rdp_simplification_directional_change_std_3
gyration_tensor_coherence
confinement_ratio
partition_moving_gyration_tensor_coherence__mean
rdp_simplification_ratio_3
rdp_simplification_ratio_0.5
speed_stats_min
partition_moving_speed_stats_min__mean
partition_moving_gyration_tensor_axis_ratio__mean
gyration_tensor_axis_ratio
gyration_tensor_radius
track_diameter_stats_mean
track_diameter_stats_max
track_diameter_stats_std
partition_moving_gyration_tensor_radius__mean
partition_moving_gyration_tensor_minor_axis_length__mean
gyration_tensor_minor_axis_length
gyration_tensor_major_axis_length
partition_moving_gyration_tensor_major_axis_length__mean
track_length
partition_moving_speed_stats_max__mean
partition_moving_speed_stats_std__mean
partition_moving_speed_stats_mean__mean
speed_stats_mean
speed_stats_std
mean_straight_line_speed
speed_stats_max
velocity_average
Parameters

Appendix Table S1 - Drug List for Time-Lapse Imaging

Drug	Function	Stock concentration	Working concentration	Dilution	Company	Catalogue #	References for Concentrations
Glutamate receptors							
CNQX	Antagonist - AMPA and Kainate receptors	10mM	10 µM	1:1000	Tocris	1045	https://doi.org/10.1016/j.stem.2019.08.002; https://doi.org/10.1523/JNEUROSCI.1033-06.2006
AP-5	Antagonist - NMDA receptors	20mM	20 µM	1:1000	Biotrend	0106	
GABA receptors							
Bicuculine Methiodide	Antagonist - GABA-A receptors	10mM	10 µM	1:1000	Tocris	2503	https://doi.org/10.1016/j.stem.2017.07.007; https://doi.org/10.1177/1073858406298918
TPMPA	Antagonist - GABA-A rho receptors	10mM	10 µM	1:1000	Tocris	1040	https://doi.org/10.1371/journal.pone.0027048
Saclofen	Antagonist - GABA-B receptors	10mM	100µM	1:100	Tocris	0246	https://doi.org/10.1038/s41467-020-17521-w; https://doi.org/10.1093/cercor/13.9.932
Glycine receptors							
Strychnine	Antagonist - Glycine receptors	1mM	1 µM	1:1000	Sigma	S0532	https://doi.org/10.1113/jphysiol.2014.278994; https://doi.org/10.1016/j.celrep.2013.07.016
Serotonin	Agonist - Serotonin receptors	100mM	100nM	1:1000000	Tocris	3547	https://doi.org/10.1038/ncomms6524; https://doi.org/10.1002/bmc.4135
SR 57227	Antagonist - HTR3A receptor	100mM	200µM	1:500	Tocris	1205	

Appendix Table S2

Antigen	Company	Catalogue #	Host	Dilution
GFP	Aves Labs	GFP1020	Chicken	1:1000
FOXG1	Abcam	AB18259	Rabbit	1:200
PAX6	R&D Systems	AF8150	Sheep	1:500
NKX2-1	Dako	M3575	Mouse	1:50
NKX2-1	Epitomics	6594-1	Rabbit	1:1000
GABA	Sigma-Aldrich	A2052	Rabbit	1:1000
NeuN	Millipore	MAB377	Mouse	1:500
DLX2	Santa Cruz	SC393879	Mouse	1:100
CAMKII	Cell Signaling	50049S	Mouse	1:100
GABA-A	Abcam	AB72445	Rabbit	1:300
GRIA2/3	Sigma-Aldrich	AB1506	Rabbit	1:300
GRIK2	Almone Labs	AGC-009	Rabbit	1:400
NMDAR1	Abcam	AB109182	Rabbit	1:100
HTR2C	Thermo	PA5-27164	Rabbit	1:300
GlyR	Santa Cruz	SC398964	Mouse	1:300

Appendix Table S3

Host	Primary	Fluorophore	Company	Catalogue #	Dilution
Donkey	Chicken	Alexa Fluor 488	Jackson Immuno	703-605-155	1:500
Donkey	Mouse	Alexa Fluor 568	Invitrogen	A10036	1:500
Donkey	Mouse	Alexa Fluor 647	Invitrogen	A31571	1:500
Donkey	Rabbit	Alexa Fluor 568	Invitrogen	A10042	1:500
Donkey	Rabbit	Alexa Fluor 647	Invitrogen	A31573	1:500
Donkey	Goat	Alexa Fluor 647	Invitrogen	A21447	1:500
Donkey	Sheep	Alexa Fluor 647	Jackson Immuno	713-605-147	1:500

Appendix Table S4

Gene	Primer 1	Primer 2
TBP	GGGCACCACTCCACTGTATC	CGAAGTGCAATGGTCTTTAGG
FOXG1	TGGCCCATGTCGCCCTTCCT	GCCGACGTGGTGCCGTTGTA
PAX6	CTGGTTGGTATCCGGGGACT	TCGCATTTGAGCCTCATCTGA
EMX2	CTGGAACACGCCTTTGAGA	CCAGCTTCTGCCTTTTGAAC
NKX2-1	GCCGTACCAGGACACCATG	ATGTTCTTGCTCACGTCCCC
GSX2	CACCGCCACCACCTACAAC	CAGGAGTTGCGTGCTAGTGA
VSX2	AGACTATAGCCCCTGGTGTG	GTGGACTCCTGGATTTCTCCC
COUPTFII/NR2F2	GCTGCACGTTGACTCAGCC	AATCTCGTCGGCTGGTTGGG
