# Supplementary material for: Neurotransmitter signaling regulates distinct phases of multimodal human interneuron migration
Source: EMBO J. 2021 Oct 18;40(23):e108714. doi: 10.15252/embj.2021108714 (PMC8634123; doi:10.15252/embj.2021108714)
Supplement: Supplementary file 2 — Expanded View Figures PDF [file EMBJ-40-e108714-s009.pdf]

Expanded View Figures

Figure EV1. Modified protocols generate region-specific cerebral organoids.

A Whole organoid scans of single organoids generated using the different protocols listed in Fig 1A. Cryosection of organoids at day 40 immunostained for markers of forebrain (FOXG1; green) and dorsal forebrain (PAX6; magenta) identity. Scale bars, 500 μm.

B–D Whole organoid scans of single organoids generated using the different protocols listed in 1A. Cryosection of organoids at day 40 immunostained for DAPI (green) and an excitatory neuron marker (B; CAMKII; magenta), an MGE marker (C; NKX2-1; magenta), or an interneuronal marker (D; DLX2; magenta). Scale bars, 500 μm.

E Quantification of area ($\mu m^2 \times 1,000$) of individual $SOX1^+$ rosettes within the single organoids generated using the different protocols at day 40. Quantification was performed on a total of 3–4 organoids per protocol. Each dot represents the size of one rosette within the analyzed organoids. Significance values, ***< 0.001. Statistical analysis was performed using one-way ANOVA and post hoc Tukey's comparison of means.

© 2021 The Authors

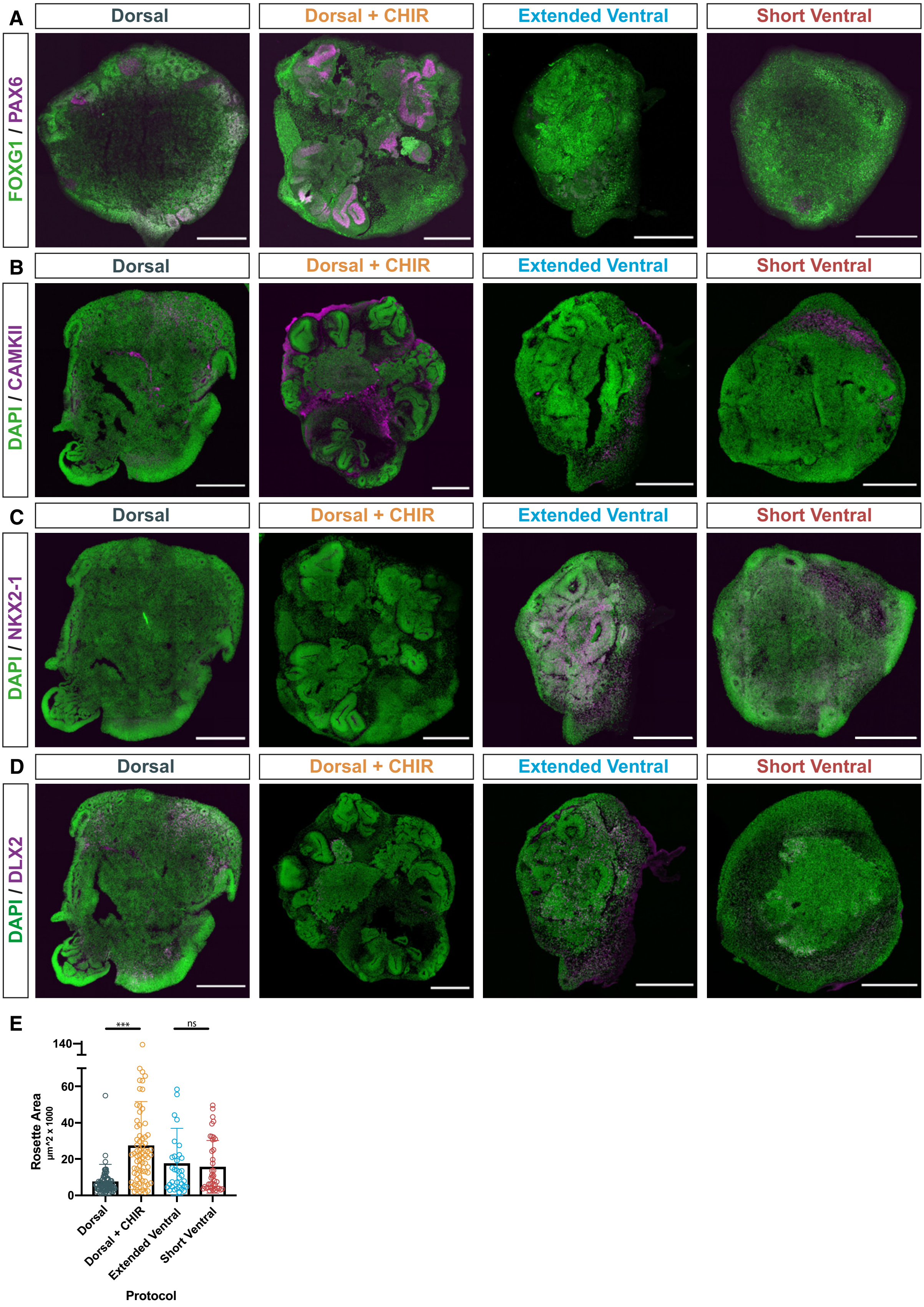
A
Dorsal
Dorsal + CHIR
Extended Ventral
Short Ventral
FOXG1 / PAX6
B
DAPI / CAMKII
C
DAPI / NKX2-1
D
DAPI / DLX2
E
***
ns
Rosette Area
µm^2 x 1000
140
60
40
20
0
Dorsal
Dorsal + CHIR
Extended Ventral
Short Ventral
Protocol

Figure EV1.

Figure EV2. RNA sequencing reveals cellular transcriptomes in organoids.

A Schematic representation of the experimental strategy for the RNA sequencing of cells from single and fused organoids. GFP^+(G) and GFP-/unlabeled(U) cells were obtained from both ventral and dorsal regions of dissected organoid fusions. G and U cells were obtained from single ventral organoids, and U cells were obtained from single dorsal organoids. In total, 7 different populations of cells were then analyzed using RNA sequencing.

B Representative visualization of fusion using brightfield imaging for identification of ventral and dorsal regions during manual dissection. Images show fusion before and after dissection.

C Flow cytometry plots for dissociated cells from either dorsal or ventral regions of dissected fusions. Cells were sorted based on their EGFP intensity (x-axis). GFP^+ proportion within each region is indicated within figure (dorsal—1.6%; ventral—29.59%). Across multiple dissections, proportions for GFP^+ cells remained consistently at 1–7% for dorsal regions and 30–50% for ventral regions.

D Heatmap depicting the weighted gene expression (z-score, bar indicating values from blue to red) of the different cell populations analyzed using RNA sequencing. Populations were stratified according to their GFP positivity, region they were isolated from, organoid age and whether they were isolated from a region of a fusion or a single organoid. Genes are sorted into groups relating to the cell type they relate to. Hierarchical clustering separates the individual samples into clusters which are labeled at the bottom.

Source data are available online for this figure.

 © 2021 The Authors

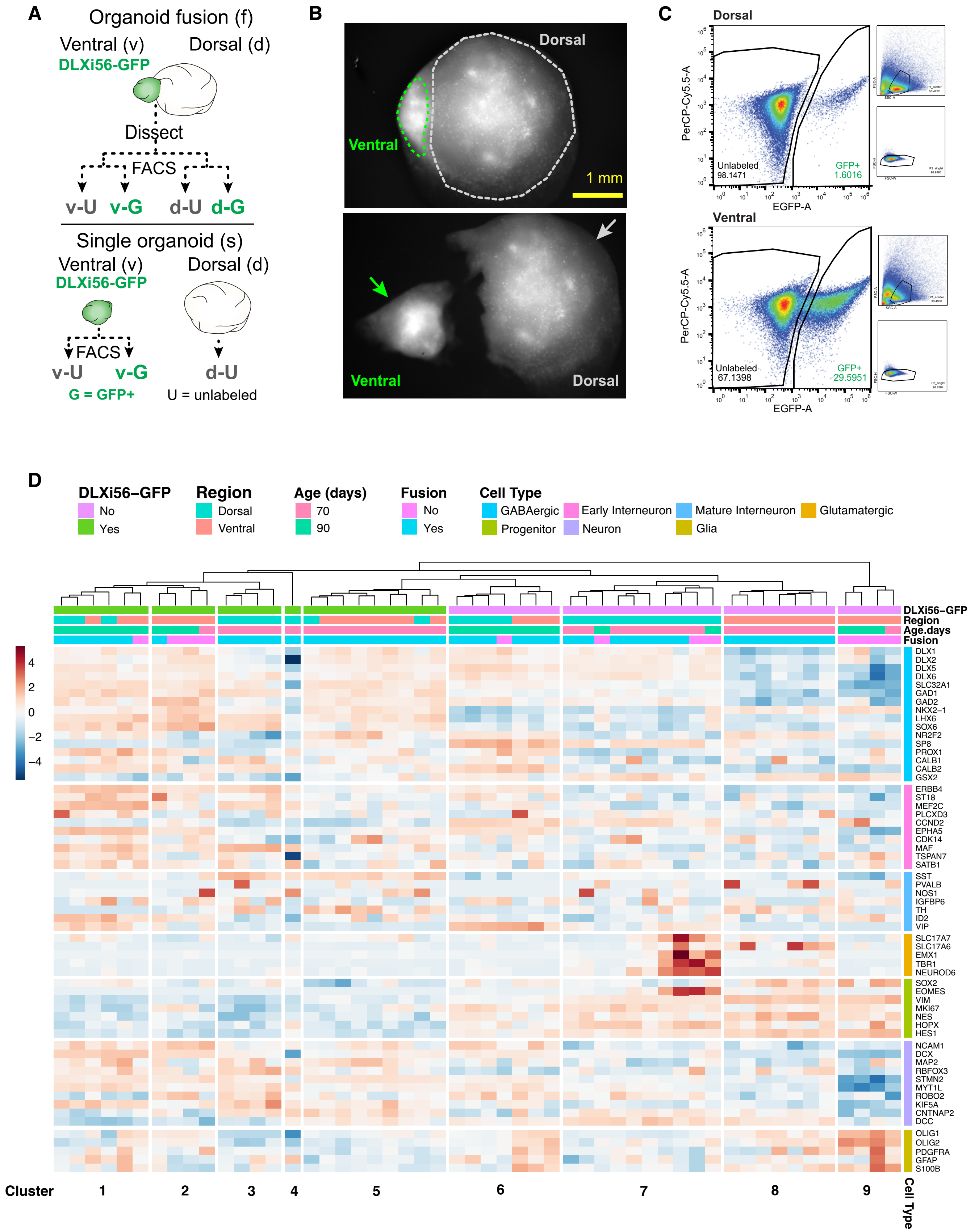
A
Organoid fusion (f)
Ventral (v)
Dorsal (d)
DLXi56-GFP
Dissect
FACS
v-U
v-G
d-U
d-G
Single organoid (s)
Ventral (v)
Dorsal (d)
DLXi56-GFP
FACS
v-U
v-G
d-U
G = GFP+
U = unlabeled
B
Dorsal
Ventral
1 mm
Ventral
Dorsal
C
Dorsal
PerCP-Cy5.5-A
Unlabeled
98.1471
GFP+
1.6016
EGFP-A
Ventral
PerCP-Cy5.5-A
Unlabeled
67.1398
GFP+
29.5951
EGFP-A
D
DLXi56–GFP
No
Yes
Region
Dorsal
Ventral
Age (days)
70
90
Fusion
No
Yes
Cell Type
GABAergic
Early Interneuron
Mature Interneuron
Glutamatergic
Progenitor
Neuron
Glia
DLXi56–GFP
Region
Age.days
Fusion
4
2
0
−2
−4
DLX1
DLX2
DLX5
DLX6
SLC32A1
GAD1
GAD2
NKX2−1
LHX6
SOX6
NR2F2
SP8
PROX1
CALB1
CALB2
GSX2
ERBB4
ST18
MEF2C
PLCXD3
CCND2
EPHA5
CDK14
MAF
TSPAN7
SATB1
SST
PVALB
NOS1
IGFBP6
TH
ID2
VIP
SLC17A7
SLC17A6
EMX1
TBR1
NEUROD6
SOX2
EOMES
VIM
MKI67
NES
HOPX
HES1
NCAM1
DCX
MAP2
RBFOX3
STMN2
MYT1L
ROBO2
KIF5A
CNTNAP2
DCC
OLIG1
OLIG2
PDGFRA
GFAP
S100B
Cluster
1
2
3
4
5
6
7
8
9
Cell Type

Figure EV2.

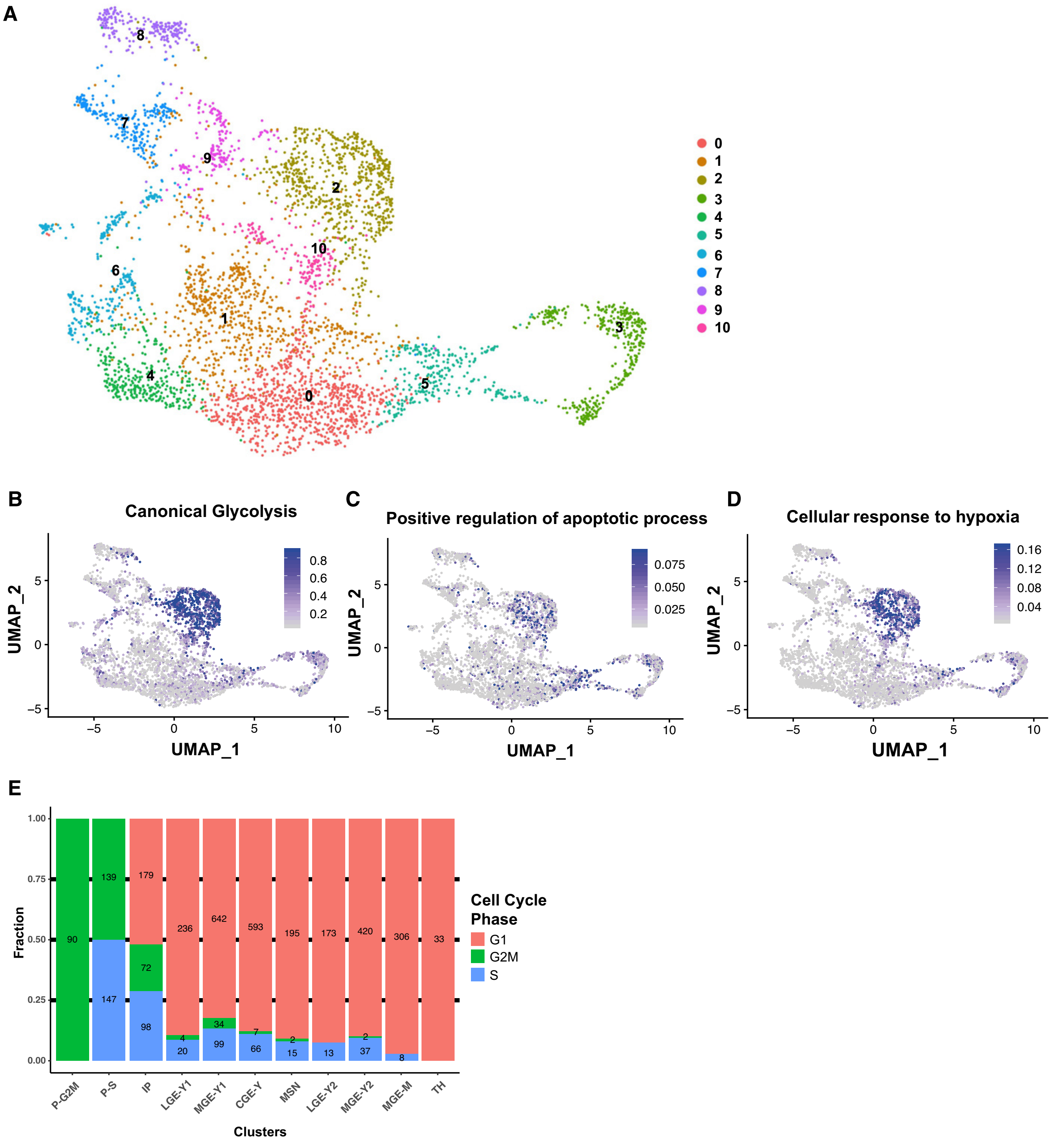

Figure EV3. scRNAseq characterizes GABAergic cells in cerebral organoid fusions.

A Visualization of single-cell RNA-sequencing data from GFP^+ cells from the different groups mentioned in Fig 2A using UMAP and color-coded based on the clustering.
B UMAP depicting the relative expression of genes linked to the GO-term "Canonical glycolysis" (GO-0061621).
C UMAP depicting the relative expression of genes linked to the GO-term "Positive regulation of apoptotic process" (GO-0043065).
D UMAP depicting the relative expression of genes linked to the GO-term "Cellular response to hypoxia" (GO-0071456).
E Proportions (y-axis) and numbers (within bars) of cells in either G1, G2 M, or S Phase of the cell cycle are listed for each cluster after filtering and removal of stressed populations.

 © 2021 The Authors

Figure EV4. Neurotransmitters differentially regulate cortical interneuron migration.

A Heatmap depicting the control-normalized values (log_2-scaled) for all 48 parameters calculated by TrackPal for the 8 receptors analyzed in Fig 5B. Gray values indicate non-significant values. For visualization purposes, parameters are grouped according to their function – direction, pausing, shape, and speed.

B Box plot visualizing the comparison of the mean speed for control and 8 treatment groups. The bars are colored as in Fig 5B to indicate their involvement in activation or inhibition of a neurotransmitter system: yellow bars—GABA, purple bars—glutamate, red bar—strychnine, blue bars—serotonin. All *P*-values are calculated with a Mann–Whitney *U*-test with Bonferroni correction. Blue central band on bars indicates the median for each group, the boxes depict values between lower and upper quartiles, the whiskers display the minimum and maximum values, and the dashed line visualizes the median for the control group for comparison. Significance values, *< 0.05, **< 0.01, ***< 0.001.

C–K Box plot visualizing: (C) Mean moving tracklet speed for control and treatment groups mentioned in Fig 5B. Analysis performed as described above. (D) Major axis length of the gyration tensor for control and treatment groups mentioned in Fig 5B. Analysis performed as described above. (E) Mean track diameter for control and treatment groups mentioned in Fig 5B. Analysis performed as described above. (F) Median of directional changes for control and treatment groups mentioned in Fig 5B. Analysis performed as described above. (G) Proportion of directional changes over 20 degrees for control and treatment groups mentioned in Fig 5B. Analysis performed as described above. (H) Mean moving tracklet directional change for control and treatment groups mentioned in Fig 5B. Analysis performed as described above. (I) Relative dwell state duration for control and treatment groups mentioned in Fig 5B. Analysis performed as described above. (J) Number of dwell states for control and treatment groups mentioned in Fig 5B. Analysis performed as described above. (K) Confinement ratio for control and treatment groups mentioned in Fig 5B. Analysis performed as described above.

© 2021 The Authors

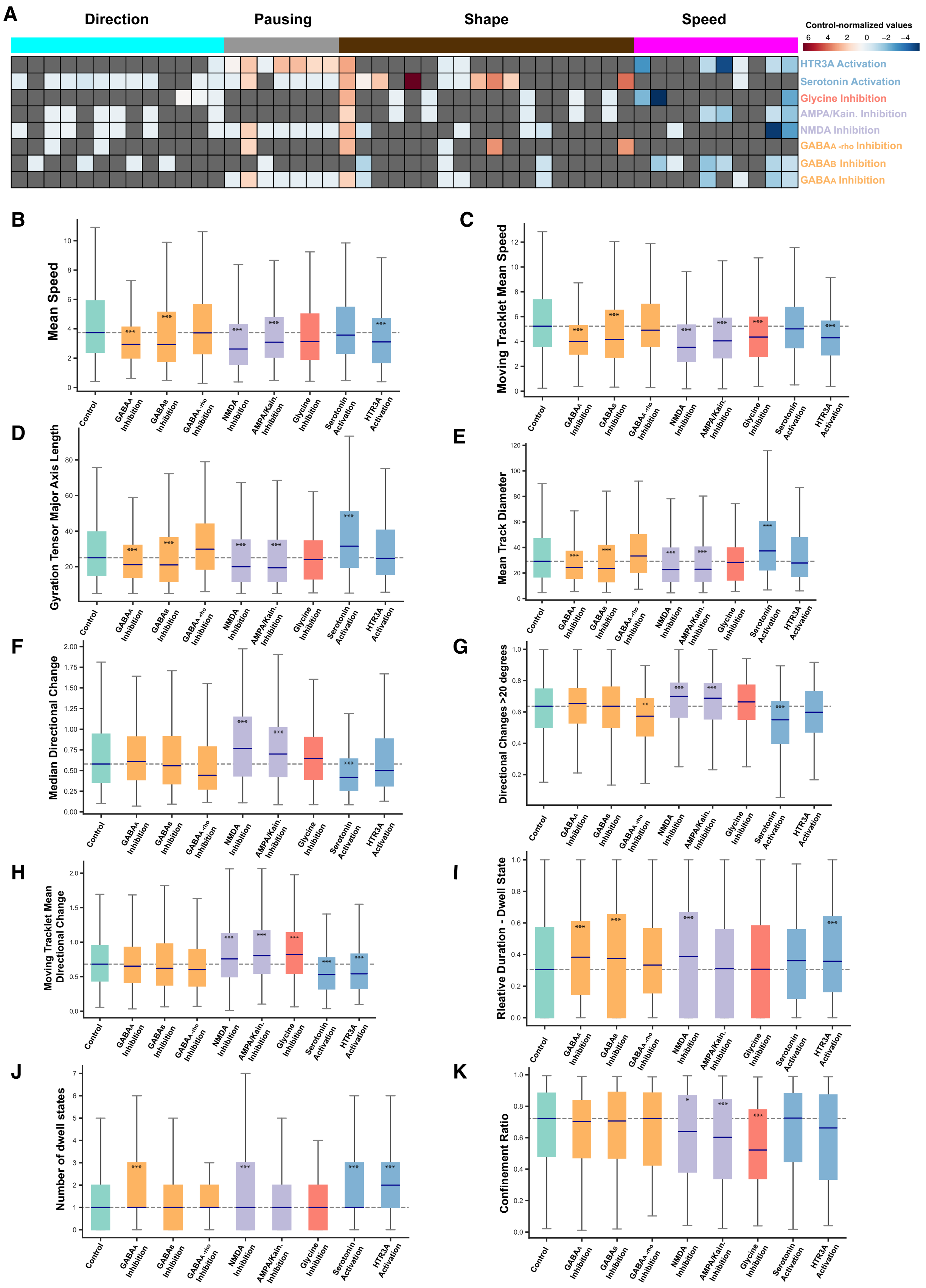

Figure EV4.

© 2021 The Authors

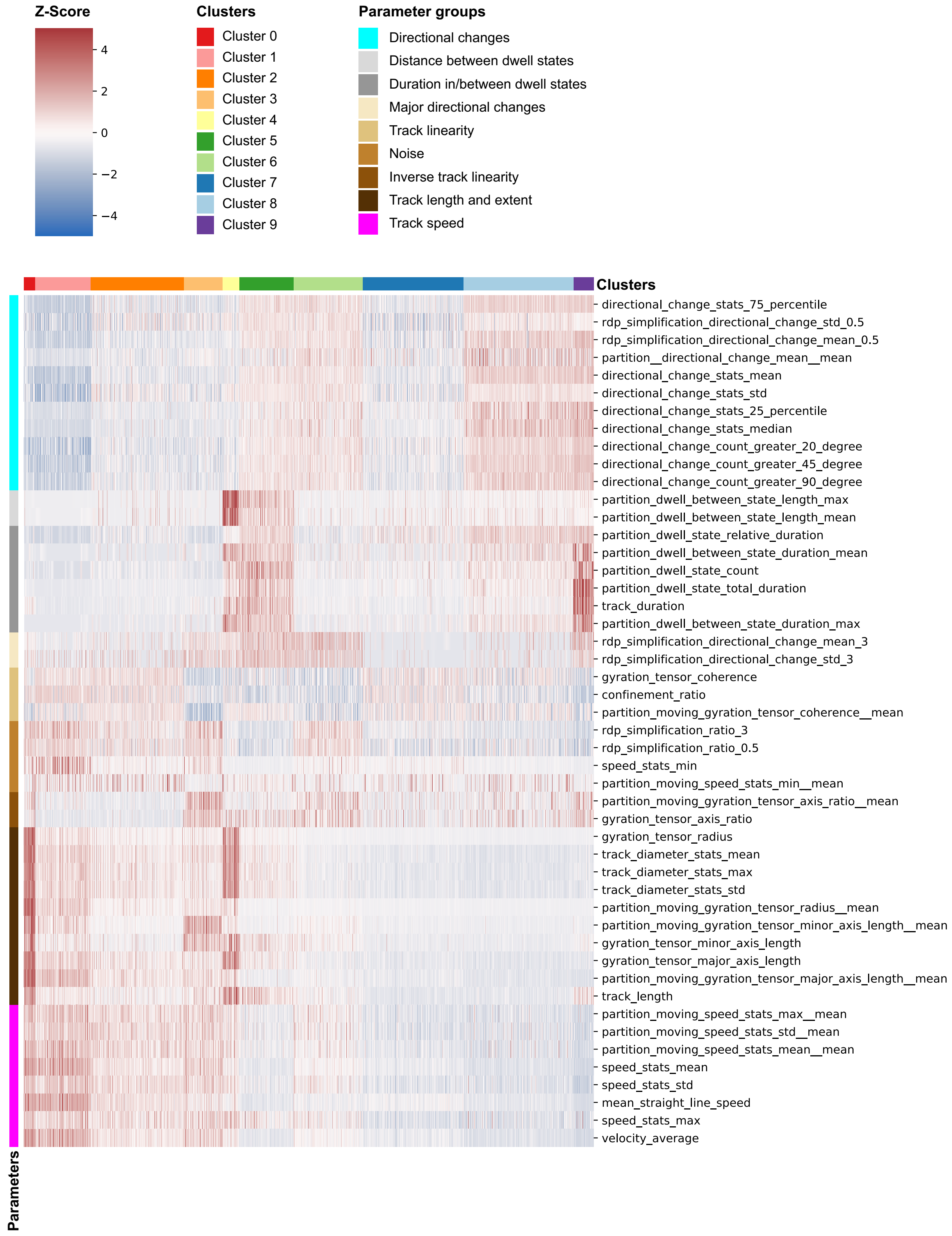

Figure EV5. Migrating cortical interneurons display migration modes.

Detailed view of heatmap in Fig 6A, depicting normalized values for 48 migration parameters for all cells tracked. Parameters are grouped into 9 different subgroups, which are color-coded and described. Cell tracks are clustered into 10 different clusters based on their expression values for the different parameters. The clusters are color-coded and numbered. The z-score scale describes the expression values.

© 2021 The Authors
