# Supplementary material for: Neurotransmitter signaling regulates distinct phases of multimodal human interneuron migration
Source: EMBO J. 2021 Oct 18;40(23):e108714. doi: 10.15252/embj.2021108714 (PMC8634123; doi:10.15252/embj.2021108714)
Supplement: Supplementary file 3 — Dataset EV1 [file EMBJ-40-e108714-s006.zip › Dataset_EV1_legend.docx]

**Dataset EV1**

Detailed descriptions of the protocols for organoid culture generation and the media used.
