# Supplementary material for: Neurotransmitter signaling regulates distinct phases of multimodal human interneuron migration
Source: EMBO J. 2021 Oct 18;40(23):e108714. doi: 10.15252/embj.2021108714 (PMC8634123; doi:10.15252/embj.2021108714)
Supplement: Supplementary file 5 — Dataset EV3 [file EMBJ-40-e108714-s016.zip › Dataset_EV3_legend.docx]

**Dataset EV3**

Detailed descriptions for all parameters calculated for analysis of migration tracking data.
