# Supplementary material for: Neurotransmitter signaling regulates distinct phases of multimodal human interneuron migration
Source: EMBO J. 2021 Oct 18;40(23):e108714. doi: 10.15252/embj.2021108714 (PMC8634123; doi:10.15252/embj.2021108714)
Supplement: Supplementary file 6 — Dataset EV4 [file EMBJ-40-e108714-s004.zip › Dataset_EV4_legend.docx]

**Dataset EV4**

Zoomed-in view of three migrating cortical interneurons captured by time-lapse imaging. Scale Bar, 20μm.
