# Supplementary material for: Neurotransmitter signaling regulates distinct phases of multimodal human interneuron migration
Source: EMBO J. 2021 Oct 18;40(23):e108714. doi: 10.15252/embj.2021108714 (PMC8634123; doi:10.15252/embj.2021108714)
Supplement: Supplementary file 8 — Movie EV2 [file EMBJ-40-e108714-s001.zip › EMBOJ-2021-108714R_Movie_EV2_legend.docx]

**Movie EV2**

Overview of migrating cortical interneurons across an entire organoid fusion captured by time-lapse imaging. Dorsal and ventral regions indicated in Figure S6B. Duration, 84 hours. Scale Bar, 100μm.
