# Supplementary material for: Neurotransmitter signaling regulates distinct phases of multimodal human interneuron migration
Source: EMBO J. 2021 Oct 18;40(23):e108714. doi: 10.15252/embj.2021108714 (PMC8634123; doi:10.15252/embj.2021108714)
Supplement: Supplementary file 10 — Movie EV4 [file EMBJ-40-e108714-s008.zip › EMBOJ-2021-108714R_Movie_EV4_legend.docx]

**Movie EV4**

Tracks for migrating cortical interneurons visualized in Movie S2. Tracks for interneurons are superposed on video and color-coded according to time bar. Duration, 84 hours. Scale Bar, 100μm.
