# Supplementary material for: Neurotransmitter signaling regulates distinct phases of multimodal human interneuron migration
Source: EMBO J. 2021 Oct 18;40(23):e108714. doi: 10.15252/embj.2021108714 (PMC8634123; doi:10.15252/embj.2021108714)
Supplement: Supplementary file 12 — Movie EV6 [file EMBJ-40-e108714-s007.zip › EMBOJ-2021-108714R_Movie_EV6_legend.docx]

**Movie EV6**

Tracks for migrating cortical interneurons across an entire organoid fusion capture by time-lapse imaging. Tracks for interneurons are superposed on video and color-coded according to time bar. Duration, 84 hours. Scale Bar, 100μm.
