# Supplementary material for: Neurotransmitter signaling regulates distinct phases of multimodal human interneuron migration
Source: EMBO J. 2021 Oct 18;40(23):e108714. doi: 10.15252/embj.2021108714 (PMC8634123; doi:10.15252/embj.2021108714)
Supplement: Supplementary file 13 — Software EV1 [file EMBJ-40-e108714-s017.zip › Supplementary_Software/Readme.docx]

**Supplementary Software**

*0. Dependencies*

Requires CPython >= 3.6 with following libraries installed:

- numpy
- pandas>=1.0.4
- scikit-image
- scikit-learn==0.21.1
- tifffile
- tqdm
- scipy
- statsmodels
- matplotlib
- rdp
- pingouin
- seaborn

*1. TrackPal source code (version 1.2.0) in folder “trackpal-1.2.0-source-code”*

We recommend installing TrackPal using the python installation program (pip)

pip install TrackPal==1.2.0

*2. Analysis scripts in folder “track-analysis-using-trackpal”*

The tracking analysis is done with the following Jupyter notebook scripts.

- 01_read-and-resave-tracks.ipynb
- 02_compute-track-features.ipynb
- 03_filter-non-moving.ipynb
- 04_feature-clustering.ipynb
- 05_msd-vac-curves.ipynb
- 06_global-parameter-statistics.ipynb
- 07_cluster-analysis.ipynb

An additional python script:

- global_constants.py

contains specific definitions such as color assignments to treatment groups and clusters and is used only from within the Jupyter notebook scripts. For further information, see the inline documentation in the notebooks
