# Supplementary figures and images for: Neurotransmitter signaling regulates distinct phases of multimodal human interneuron migration
Source: EMBO J. 2021 Oct 18;40(23):e108714. doi: 10.15252/embj.2021108714 (PMC8634123; doi:10.15252/embj.2021108714)

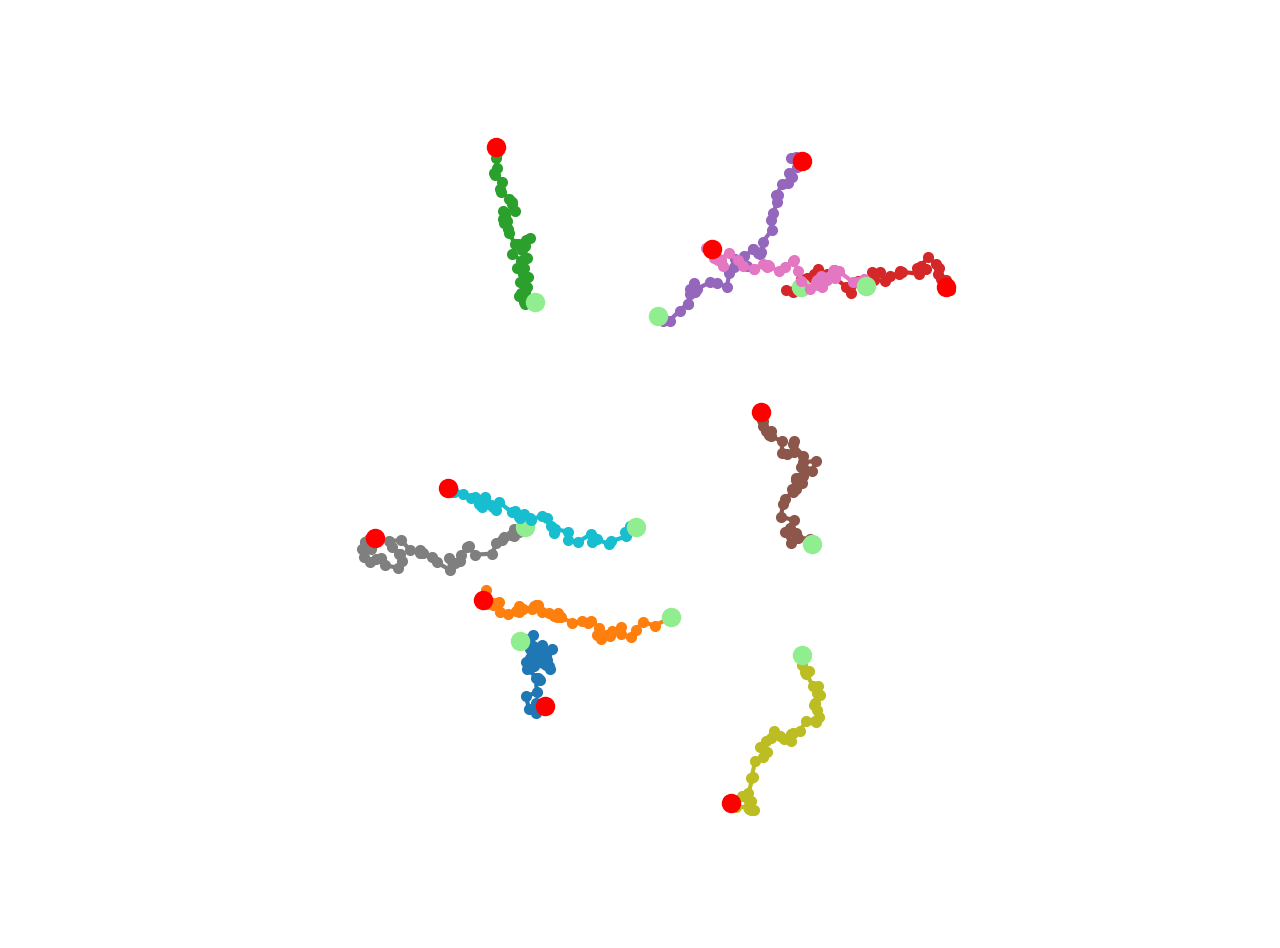

Supplement: Supplementary file 13 — Software EV1 [file EMBJ-40-e108714-s017.zip › Supplementary_Software/trackpal-1.2.0-source-code/doc/img/bl_tracks_01.png]
