# Supplementary material for: Neurotransmitter signaling regulates distinct phases of multimodal human interneuron migration
Source: EMBO J. 2021 Oct 18;40(23):e108714. doi: 10.15252/embj.2021108714 (PMC8634123; doi:10.15252/embj.2021108714)
Supplement: Supplementary file 14 — Source Data for Expanded View and Appendix [file EMBJ-40-e108714-s015.zip › Source_Data_for_Expanded_View_and_Appendix/Figures_EV2_AppendixFIgureS3/Source_Data_Expanded_View_Appendix_Table1_legend.docx]

**Source Data Table for Expanded View and Appendix 1**

List of gene counts for all samples analyzed using the bulk RNA sequencing. This data can be used to re-perform the complete analysis and reproduce Figures EV2D and Appendix Figure S3A.
