# Supplementary material for: Neurotransmitter signaling regulates distinct phases of multimodal human interneuron migration
Source: EMBO J. 2021 Oct 18;40(23):e108714. doi: 10.15252/embj.2021108714 (PMC8634123; doi:10.15252/embj.2021108714)
Supplement: Supplementary file 15 — Source Data for Figure 5 [file EMBJ-40-e108714-s014.zip › EMBOJ-2021-108714R1_Source_Data_For_Figure_5C_legend.docx]

**Source Data for Figure 5C and EV4**

List of significant values for each parameter for all cells in a group (drug or control) which was used for analysis and creation of Figures 5C and EV4.
