# Supplementary material for: Neurotransmitter signaling regulates distinct phases of multimodal human interneuron migration
Source: EMBO J. 2021 Oct 18;40(23):e108714. doi: 10.15252/embj.2021108714 (PMC8634123; doi:10.15252/embj.2021108714)
Supplement: Supplementary file 16 — Source Data for Figures 5 and 6 [file EMBJ-40-e108714-s010.zip › EMBOJ-2021-108714R1_Source_Data_For_Figure5+6_legend.docx]

**Source Data for Figures 5 and 6**

List of values for all parameters for each individual cell analyzed in the tracing analysis using live-imaging. Individual cells are labeled according to the group they belong to. This data can be used to re-perform the entire migration analysis and create Figures 5-6.
