# Supplementary material for: Neurotransmitter signaling regulates distinct phases of multimodal human interneuron migration
Source: EMBO J. 2021 Oct 18;40(23):e108714. doi: 10.15252/embj.2021108714 (PMC8634123; doi:10.15252/embj.2021108714)
Supplement: Supplementary file 17 [file EMBJ-40-e108714-s011.pdf]

THE EMBO JOURNAL

Neurotransmitter signaling regulates distinct phases of multimodal human interneuron migration

Sunanjay Bajaj, Joshua Bagley, Christoph Sommer, Abel Vertesy, Sakurako Wong, Veronica Krenn, Julie Lévi-Strauss and Juergen Knoblich
DOI: 10.15252/embj.2021108714

Corresponding author(s): Juergen Knoblich (juergen.knoblich@imba.oeaw.ac.at)

Review Timeline:

Submission Date:	13th May 21
Editorial Decision:	2nd Jul 21
Revision Received:	30th Aug 21
Editorial Decision:	21st Sep 21
Revision Received:	26th Sep 21
Accepted:	28th Sep 21

Editor: Karin Dumstrei

Transaction Report:

(Note: With the exception of the correction of typographical or spelling errors that could be a source of ambiguity, letters and reports are not edited. Depending on transfer agreements, referee reports obtained elsewhere may or may not be included in this compilation. Referee reports are anonymous unless the Referee chooses to sign their reports.)

1st Editorial Decision 2nd Jul 2021

Dear Jürgen,

Thank you for submitting your manuscript to The EMBO Journal. I am sorry for the delay in getting back to you with a decision, but I have now received the three referee reports.

As you can see from the comments, the referees find the analysis interesting and appreciate the breath of the analysis. I would like to invite you to submit a revised manuscript that addresses the raised issues.

As you can see from the comments, referees #1 and 2 raise issues regarding conceptual novelty and if we gain enough mechanistic insight. Referee #3 has some comments/suggestions regarding the specific experiments.

I think the value of the paper lies in that you provide a comprehensive analysis of interneuron migration using hESC-derived interneurons. If somethings have previously been shown in rodents or human models that doesn't take away from the current analysis as long as this is clearly acknowledged and discussed. Regarding the question about mechanism - if you have any data hand to address the constructive comments raised by the referees please include it. Otherwise let's discuss this issue further. What is important is to have a well-validated data set.

I think it would be helpful to discuss the revisions further via email or a video call. Let me know what works best for you

When preparing your letter of response to the referees' comments, please bear in mind that this will form part of the Review Process File, and will therefore be available online to the community. For more details on our Transparent Editorial Process, please visit our website: https://www.embopress.org/page/journal/14602075/authorguide#transparentprocess

I thank you for the opportunity to consider your work for publication. I look forward to discussing your revisions further with you

I have attached a document with helpful tips on how to prepare the revised version. Please pay attention to the parts on the Data Availability Section and figure legends.

with best wishes

Karin

Karin Dumstrei, PhD
Senior Editor
The EMBO Journal

Guide For Authors: https://www.embopress.org/page/journal/14602075/authorguide

Revision to The EMBO Journal should be submitted online within 90 days, unless an extension has been requested and approved by the editor; please click on the link below to submit the revision online before 30th Sep 2021:

https://emboj.msubmit.net/cgi-bin/main.plex

Referee #1:

This paper is another testament of the quality and robustness of the Knoblich laboratory. The use of cerebral organdies for studying interneuron migration and its specific modes in particular is emphasised here. The paper encompasses single-cellRNA-seq at time points, clustering and molecular profiling, gene and receptor identification, the development and use of novel tracking algorithms and others. Quite clearly, this is a refined and technically immaculate paper. Nevertheless, I believe this is one of its problems, too, because some of the technicalities cast shadow on conceptual novelties. In fact, many elements of the data were shown (or interpreted as such) earlier in rodent and human experiments/models.

Specific comments:
1. A recurring debate is that "organdies are not human brains". Therefore, it would be helpful to place the age of organdies in vitro into some human developmental perspective (i.e. 40 vs. 60 days, 70 vs. 90 days in vitro). Likewise, it would be helpful to show that all cellular components that allow for both tangential and vertical (intracortical) migration are present (Kgs, pyramidal cells), for all interneuron subtypes.
2. Some of the experiments are done at different time points in culture. One would wish to see a justification that these do not affect data interpretation and amalgamating the various aspects of the paper.

3. How do the 11 in vitro clusters of GABA cells relate to/equate what is reported in human cortex (ie coverage, neurochemical identities)?
4. It would be necessary to control for the expression of the GFP reporter in combination with in situ hybridisation. I.e. that no interneuron population is missed because of the lack of Dlx gene expression.
5. When the authors discuss the presence of strital interneuron identities in ventral organoids, do the cells that cannot route to the striatum will be eliminated or instead continue to aggregate at a ventral node? Given this physical barrier, do stratal interneurons reroute to cortical sites (aberrant migration) upon pharmacological challenges (i.e. cannabinoid and glutamate receptor KOs exhibit such phenotypes).
6. Oe would expect that organoids cease the production of new neurons if they recapitulate human development. Does this occur after 90 days? Is there a reduction in proliferative potential between 70 and 90 days? If not, one could hypothesize that enrichment in neuronal subclasses is a stochastic process and unrelated to what is reported in post-morteum tissues.
7. On page 7, it would be helpful to add a sentence and reference to papers from eg the Kriegstein lab on receptor expression in prenatal interneurons in vivo. Receptor profiling of human interneurons during development has been the subject of earlier studies, which should be mentioned.
8. For TrackPal, it would be great to have direct comparison on its precision in perhaps a simpler system, e.g. IncuCyte scratch-wound assay or similar and migrating human neurons in 2D or 3D.
9. Reference to papers from the Ben-Ari lab on interneuron migration vs. GABA and glutamate receptor function in mouse cortex should be given. Even if mouse work, yet most of the phenotypic changes that are described here are known, and hence credit should be given where it is due.
10. One wonders why interneurons intrinsically express 5HT2C receptors if 5HT is not available. How much is the 5HT receptor expression pattern biased by the model (i.e. comparison to human fetal expression patterns shall be presented). 5HTR2C mediates excitatory transmission. It is coded on the X chromosome in humans - i.e. does the gender identity of the organoid affect expression and/or function/responses? 5HTR2C receptors seem to modulate eg dopamine release by being presynaptic. Are these receptors in e.g. the growth cones of GABA interneurons for chemotropic guidance and subsequently at presynaptic locations once the cells become stationary? Can one elicit directional migration by serotonin in organoids (as of "fewer directional changes")? It would be particularly interesting if these interneurons were sensitive to antidepressant action (i.e. dose-dependent antidepressant action at a subthreshold extracellular 5HT concentration), which could significantly increase the novelty value of the paper.
11. Similarly, the authors mention cannabinoid receptors: why were these not tested functionally? Cannabinoid receptors are selectively on CCK interneurons (i.e. CGE-derived/5HT3A interneurons), and will induce aberrant migration and growth cone retraction. Showing selectivity to THC or a prototypic CB1R ligand (WIN55,212-2 or HU210 could work well) could again be of significant functional value.
12. what are the mechanistic underpinnings of the different modes of migration? Can one migratory mode transit into the other (e.g. upon disruption for cytoskeleton or pharmacological challenge?).

In sum, this is a very well elaborated paper. Yet increasing its value in terms of novel discovery and mechanistic impact can be seen as a must for it to be out of a "niche" modelling paper.

Referee #2:

This manuscript describes an advanced method to generate hESC-derived assembloids where the ventral and dorsal forebrain patterning steps are improved to generate cells that better recapitulate features of cortical interneurons after fusion. Moreover, the authors report a novel automatized tracking tool to assess cell migration, thereby showing that cortical interneurons navigating into the dorsalized regions of the assembloid (which is devoid of tissue organization) tend to show moving behavior reminiscent of what has been described in the mouse cortical wall (guided, exploratory/dispersed, and more confined migration). These observations are interesting and suggest that an important part of the multimodal migration of cortical interneurons is cell-intrinsically programmed. The work also focuses on the role played by some metabotropic and ligand-gated ion channel receptors activated by neurotransmitters during migration. This has previously been described in rodent, but never in hESC-derived interneurons. Overall, the manuscript provides a new set of biological and analysis tools that will be of great interest for neuro- an cell biologists. However, while there is no doubt that the data have been carefully generated, the manuscript in its present form reports interesting scientific observations that need further validation. Moreover, this work fails short in providing a mechanistic understanding of how distinct neurotransmitters impact differently cortical interneuron migration in assembloids.

Major comments

> Regarding Figure 1 and the advanced method to generate assembloids. Since organoids have been generated via a new method, the dorsal and extend ventral organoids need further analysis (proportion of cycling cells, survival...). The reviewer also suggests to better match the name of dorsal organoids generated classically or by the improved method (as this is the case for the ventral ones) to help the reading of non-specialists. The extended ventral protocol seems to present more ventricle as compared to control, is that is significative? (Fig S1A-D). It it surprising that ventrally and dorsally patterned organoids are fused at such unequal size (see fig 1D). This has not yet been reported (Sloan 2018, Birey 2017, Bagley 2017) and should be clearly explained if it represents and additional step of the improved protocol.

>The figure 1F show that the dorsal region is full of GFP-negative cells that express GABA, what are they?

>In Figure 2E, the number of striatal neurons is higher in 70-days old organoids (127) as compared to 90-days old (34). This suggests a temporal bias of generation of these neurons in organoids and this should be discussed.

>The data presented in figure 3A and 3B are unclear and need to be better explained and represented for non-specialists.

>The analysis of the scRNAseq data obtained from the assembloids provide the demonstration that human-derived cortical interneurons express bona fide markers as well as an important repertoire of neurotransmitters receptors with dynamic expression across maturation stages. These data need further validation by qRT-PCR and immunostainings (when ab are available). Moreover, there is no functional demonstration (electrophysiology, calcium imaging..) that these receptors can be pharmacologically activated/ blocked and there is no clue provided about the source of neurotransmitters in assembloids.

>The figure 5 is very dense and not easy to understand at first look. This is particularly the case when trying to compare figure 5C and D. How can you extract simple effect (green and red arrows/lines) of the activation of neurotransmitter receptor (which is indeed not always demonstrated as blockers and not neurotransmitters are used in the culture medium) as represented in figure 5D when blockers impact so many migration parameters, as shown on the heatmap of figure 5C?

>The blockade of glutamate receptors alters the directionality of movement of interneurons. It would be interesting to test whether GluRs are indeed necessary to initiate migration from ventral to dorsal regions (AP5/CNQX treatment before migration initiation at 40-50 days). Moreover, along this line, it would be great to know whether the inhibition of GABA-rho and GluRs at early time points (40-50 days) alters the density of migrating interneurons in the dorsal part (proximal vs distal area to ventral area).

>While the author suggest that the pharmacological inhibition of distinct neurotransmitter receptors alters the migration of interneurons in organoids, they haven't addressed the underlying mechanisms. Moreover, it is unclear whether these changes are strictly cell autonomous as cortical interneurons migrate between other dorsal cells that are likely to express neurotransmitter receptors. Thus, it is necessary to test whether KD neurotransmitters receptors directly in interneurons (shRNA/miRNA/CrispR) affect their migration in dorsal area of assembloids.

> The figure 6 nicely shows the multimodal migration mode adopted by cortical interneurons. Can authors correlates this observation with position in the dorsal areas of assembloids (proximal vs distal to the ventral area).

Specific comments
> Line 120-129 and Fig 1 A: The authors should provide a better description of the protocols used and make them easy- to read for non-specialists
> Fig 5D: Not legend about arrows, etc.
>Lines 475-476: inhibition of GABAB signaling "reduced" the proportion of cells demonstrating CM should be replaced by "increased"

Referee #3:

In this manuscript by Bajaj and colleagues, the authors postulate that different neurotransmitter signaling pathways impact on the interneuron migration mode. They introduce an improved patterning protocol used prior to fusion of dorsally and ventrally pre-patterned brain organoids. To allow cell-type specific tracing of migrated interneurons within dorsal structures of fused organoids, they generate a transgenic reporter (DLXi56-GFP) pluripotent stem cell line. scRNA-seq was then used to characterize interneurons and in particular the migrated cells on a molecular level. Transcriptomic characterization of GFP-positive FAC sorted cells revealed the existence of all major human forebrain GABAergic populations. Next, the authors put a particular focus on migrating interneurons by pseudotemporal reconstruction of development trajectories. Increasing expression of several neurotransmitter receptor genes correlating with the degree of maturation was observed. In the second part of the study the authors aimed at studying how neurotransmitter signaling would impact on interneuron migration behaviors. Therefore, they developed an image analysis software package to study neuronal migration by implementing 48 different track parameters. This was used to analyze about 4000 interneurons migration tracks including the ones of neurotransmitter treated cells and revealed distinct alterations of neurotransmitter-treated cell migration modes. Unfortunately the final analyses need some refinement.
In sum, this is a very interesting study which allows detailed dissection of interneuron migration modes by applying TrackPal to the organoid fusion system. However, I have some concerns, suggestions, and comments as specified below.

Major points
• On d70 and d90 fused organoids were dissected to perform scRNA-seq of the ventral and dorsal regions. Given the example picture in Fig. 1E and the fact that there are many GFP cells in the previously dorsally patterned organoids, I wonder whether

GFP expression is accurate enough. Or is it the size difference that is used? Can the authors provide a picture and/or comment on this aspect? What is the fraction of GFP-positive cells in the dorsal parts?
• To me it is surprising and rather counterintuitive that there are more progenitors in the d90 organoids than in the d70 ventral regions (Fig. 1E). Can the authors comment on this?
• The authors state in the discussion that the maturation is migration-dependent. There is no evidence shown that the maturation is indeed dependent on the migration. Are there no comparably mature interneurons in the ventral part? In this context it would be good to see how corresponding interneuron transcriptomes look like without fusion?
• The authors performed pseudotemporal analyses using all cells introducing that they want to focus on the cells that migrated. I wonder whether it wouldn´t be advisable to selectively focus on the GFP+ cells isolated from the dorsal regions since these had been migrated there. If they did then it was not clear. Also, I did not find how many cells were included in the pseudotemporal analysis (see minor comments below).
• Concerning the clustering approach to classify different migratory behaviors in Fig. 6. According to the methods section, the authors performed first PCA analysis and clustering of 696 control interneurons and then assigned the closest control cluster ID to the treated interneurons. In the main text however, they mention that all tracked interneurons were utilized to increase the robustness of the clustering. The authors should clarify this. Why not cluster all (tracks of non-treated and treated cells) together? What if there is completely new migration mode?
• The authors provide a mathematical framework to group the migration modes into 9 clusters. However, the higher-order groups (directed, explanatory, confined) are hand-picked and not based on an unbiased classification. This requires a clearly defined, unbiased, and reproducible approach. It is essential, in particular because the effect of the neurotransmitter treatments on the migration modes is based on this higher-order groupings.
• Through the treatments they see shifting of the fractions of directed, exploratory, and confined modes (Fig. 6F). They should include statistics to show that the described effects are meaningful.
• Further, I wonder whether they can exclude that shifting towards the confined mode (e.g. through NMDA inhibition) instead means increased maturation. This could be assessed by scRNA-seq of neurotransmitter treated organoids.

Minor points
• The authors mention medium change on d3 for the generation of dorsal EBs. This is not indicated in Fig. 1A.
• In the box within Fig. 1A it says: Imp+/-A - Differentiation medium. I am assuming that this refers to either improved differentiation medium minus (-) or plus (+) Vitamin A? It is not specified in the methods section that Imp+A is used until d20 but stated that it is added only after fusion and transfer of the organoids on the orbital shaker on d25-26.
• It is obvious that the ventrally patterned organoids are smaller in size although they started out with the same cell number. Can the authors comment on this?
• After some filtering and selection steps, the authors started out from 3635 cells. How many cells are included in the pseudotemporal analysis in Fig. 3A? This should be indicated somewhere, e.g. in the Figure legend.
• I think it would be interesting if the authors would comment on whether the migration behavior could also be studied in a less complex system like directly differentiated neurons in 2D.

General response to the reviewers' comments
We would like to thank all three reviewers for carefully assessing our manuscript and for advising us on how it can be improved. We hope we were able to address all their concerns in this revised version.

Summary of reviewers' comments
Our manuscript uses a transgenic GFP reporter to track the migration of cortical interneurons in human cerebral organoid fusions from their origins in the ventral forebrain organoids into the developing cortical regions in the dorsal forebrain. We provide an in-depth characterization of the migration using a cell tracking analysis platform, TrackPal, and determine the role of neurotransmitters in modulating human interneuron migration. In general, all the reviewers highlighted the importance and quality of the transcriptomic and tracking analysis. The reviewers further appreciated that our study investigates for the first time the modulation of interneuron migration by neurotransmitters in a human system. The depth of our analysis was acknowledged to be technically sound and thorough. However, all reviewers shared **specific concerns** regarding the validation of the observations:
All three reviewers requested clarification regarding the protocols for organoid generation. Reviewer 1 highlighted questions regarding the specificity of the Dlxi56-eGFP reporter, discussion about the comparison of our observed GABAergic cell types to known cell types in fetal human tissue, and the localization of receptors in the migrating interneurons. Reviewer 2 emphasized the need for validation of the neurotransmitter receptor expression in interneurons and the discussion of the possible mechanisms that regulate migration. Reviewer 3 requested further insight into the proliferative potential in organoids, the migration track clustering analysis of TrackPal and possible mechanisms of modulation.

Main modifications
To address the remarks, we now include 4 new supplementary figures, 1 new supplementary table and adapted versions of several existing figures. We provide new data from a bulk RNA sequencing experiment which details the transcriptomic identity of both organoid fusions and non-fused organoids, new immunohistochemical analysis of the localization of neurotransmitter receptors in cortical interneurons, quantifications to highlight the proliferative potential of organoids, and further clarification of the TrackPal cell track analysis software. The reviewer comments prompted us to include additional data which we believe greatly enhance our study, and we hope the reviewers will agree that the revised manuscript addresses all the criticism that was raised on the first version.

Referee #1:

> This paper is another testament of the quality and robustness of the Knoblich laboratory. The use of cerebral organdies for studying interneuron migration and its specific modes in particular is emphasised here. The paper encompasses single-cellRNA-seq at time points, clustering and molecular profiling, gene and receptor identification, the development and use of novel tracking algorithms and others. Quite clearly, this is a refined and technically immaculate paper. Nevertheless, I believe this is one of its problems, too, because some of the technicalities cast shadow on conceptual novelties. In fact, many elements of the data were shown (or interpreted as such) earlier in rodent and human experiments/models.
>
> Specific comments:
> 1.1 A recurring debate is that "organdies are not human brains". Therefore, it would be helpful to place the age of organdies in vitro into some human developmental perspective (i.e. 40 vs. 60 days, 70 vs. 90 days in vitro). Likewise, it would be helpful to show that all cellular components that allow for both tangential and vertical (intracortical) migration are present (Kgs, pyramidal cells), for all interneuron subtypes.

We appreciate the reviewer's comment regarding the developmental comparison of the fetal human brain and human brain organoids. Previous studies compared in vitro to in vivo human brain development (Lancaster *et al*, 2013; Velasco *et al*, 2019). The cortical plate forms after ˜2 months (60 days) of fetal brain development, which has also been recapitulated in cerebral organoids (Lancaster *et al*, 2017). Therefore, from a developmental perspective, the 40-60 compared to 70-90 day timepoints relate to before and after cortical plate formation, respectively. Interneurons initially perform stream migration at early embryonic stages (Ang *et al*, 2003), enter the developing cortical structure and perform complex multidirectional intracortical migration, which has been shown to extend well into postnatal stages in both mouse (Faux *et al*, 2012) and human (Arshad *et al*, 2016; Paredes *et al*, 2016b). By analyzing the migration of cortical interneurons in organoid fusions at this later stage of development (60 days onwards) we therefore aimed to capture both the directed, stream migration as well as the more complex intracortical dispersion of migrating interneurons. We described this logic in the original manuscript in the discussion of the migration mode, but have added additional explanation as requested by the reviewer to emphasize the importance of the timing of the experiments (Lines 198-201, 470-476).
Regarding all the cellular components that allow for both tangential and vertical migration, the regional identities required for both types of migration is recreated in dorsal-ventral organoid fusions. This has been described previously (Bagley *et al*, 2017; Birey *et al*, 2017; Xiang *et al*, 2017) and we are extending the analysis of this migratory behavior. In Figure S1B we show that CAMKII-expressing pyramidal cells are present in the cortical regions of the fusions as shown by immunofluorescence. We now further include a bulk RNA-seq experiment addressing the presence of other cell types such as pyramidal neurons within fusions as suggested by the reviewer as Supplementary Figure 2. Herein, we perform RNA sequencing for both GFP+ and GFP- cells from both ventral and dorsal regions of fusions (Figure S2A),

which allows us to capture the presence of pyramidal cells (cluster 7. Figure S2D) and glial cells (cluster 8-9, Figure S2D) which are not labeled by the DLXi56-eGFP reporter (Lines 167-185). This confirms the presence of all cellular components required for correct intracortical interneuron migration. This data enhances the developmental context and characterization of our system, and we thank the reviewer for this suggestion.

> 1.2. Some of the experiments are done at different time points in culture. One would wish to see a justification that these do not affect data interpretation and amalgamating the various aspects of the paper.

We thank the reviewer for their important comment on the different time points of our analyses, which may not be clear enough in the paper. However, we are unsure to which exact experiments the reviewer is referring. Nonetheless, we feel this should be clarified as the reviewer suggests: Since one purpose of our analysis was to assess maturation status, the use of different timepoints was required to be able to compare different ages (maturation stages). This is now clearly explained in the results section introducing the scRNA-seq analysis (Lines 198-201).

> 1.3. How do the 11 in vitro clusters of GABA cells relate to/equate what is reported in human cortex (ie coverage, neurochemical identities)?

We welcome the reviewer's question regarding the comparison of the observed cell types with transcriptomic data from human cortical tissue. Yes, our single cell RNA sequencing dataset does cover the diversity of GABAergic cells reported in the human cortex, further indicating the faithful transcriptomic representation of the human cortex in the human cerebral organoid system.
The entire diversity of interneurons observed in the brain is derived from the ganglionic eminences (GEs) of the ventral forebrain, with both cortical and striatal interneurons predominantly generated in the medial (MGE) and caudal regions (CGE) whereas the striatal projection neurons are mostly derived from the LGE (Marín & Rubenstein, 2003). In our analysis, we can differentiate the cells generated from these regions and properly classify subtypes within these groups.
Firstly, we observe progenitors expressing well known markers TOP2A and NUSAP1 (Figure 2C, S4A) and intermediate progenitor cells expressing known markers VIM, HES6 and NES (Figure S4A). These cells importantly express DLX genes (Figure 2C), indicating they are indeed GABAergic progenitors. This is consistent with the transcriptomic identity of progenitors in mouse GE tissue (Mayer *et al*, 2018). A recent pre-print from the Kriegstein lab indicates this to be consistent with cells from human ganglionic eminence tissue as well (Velmeshev *et al*, 2021). Next we observe the stratification of the post-mitotic neurons into their respective fates.
The molecular signature of cortical interneurons derived from the MGE and the CGE changes during the maturation of these interneurons. Young MGE interneurons express markers LHX6 and SOX6, while young CGE neurons express markers NR2F1, NR2F2 and PAX6 (Figures 2B-C, S4C-D and S5A). This is well established in mouse GE tissue (Mayer *et al*, 2018) but appears to be true in human as well (Velmeshev *et al*, 2021). We observe this exact signature within our analysis and can identify these populations of cells termed as MGE-young and CGE-young (Figure 2B). Transcriptomic data of mature cortical interneurons derived from human

forebrain tissue has been analyzed in multiple previous studies (Krienen *et al*, 2020; Zhong *et al*, 2018; Hodge *et al*, 2019). Four major classes of mature interneurons are observed across all these studies – SST+ and PVALB+ interneurons from the MGE; VIP+ and LAMP5+ interneurons from the CGE. In our current analysis we only observe the presence of SST+ interneurons, which we define as MGE-mature (Figure 2B). However, we can already see the expression of MEF2C (Figure S4C), a known early marker of PVALB+ interneurons(Mayer *et al*, 2018), indicating the generation of PVALB+ interneurons as well. We do not see the presence of mature CGE interneuron subtypes. As we perform scRNA-seq analysis on only 90 day old organoid fusions, it is expected that not all mature subtypes can be observed. Furthermore, it has been noted previously that CGE interneurons may be generated later in development and continue to mature well into postnatal stages (Faux *et al*, 2012; Mayer *et al*, 2018), further explaining the lack of mature CGE interneuron subtypes. A recent pre-print from the Knoblich lab provides further evidence of the delayed generation of CGE interneurons and their importance for proper brain development (Eichmüller *et al*, 2020).
Finally, we also observe the existence of striatal interneuron and medium spiny neuron (MSN) subtypes , which remain in ventral regions of the cortex and form the striatum. Most of the information relating to the transcriptomic identity of striatal cells has been obtained from mouse striatal tissue (Muñoz-Manchado *et al*, 2018; Saunders *et al*, 2018; Gokce *et al*, 2016). Striatal interneurons can hereby be differentiated into five major subtypes – NPY/SST, NPY/MIa, CHAT+, TH+ and PTHrP+ striatal interneurons. Recent human datasets appear to indicate the existence of these subtypes as well (Krienen *et al*, 2020). In our dataset, we can identify LHX8+ cholinergic interneurons which make up a part of the CHAT+ cholinergic interneurons (Ahmed *et al*, 2019) and few TH+ interneurons (Figure 2B,D and S5A). As these interneurons are also generated at later stages of embryonic development (Muñoz-Manchado *et al*, 2018), we may only be able to observe such mature populations at later stages of organoid development. Finally, we also observed developing and more mature MSN (Figure 2B, S4E-F) expressing markers such as SIX3, ISL1 and EBF1 (Gokce *et al*, 2016), indicating the development of the different cells generated during striatal development.
Overall, our single-cell RNAseq analysis of DLXi56-eGFP positive cells derived from cerebral organoid fusions encompasses a broad assortment of the currently known GABAergic cells from both mouse and human forebrain development. However, we do not see certain mature subtypes of cortical and striatal interneurons, since we only perform scRNA-seq analysis up to 90 days of organoid development. This was intentional because our focus was on interneuron migration rather than cortical maturation. Future studies analyzing fusions at more advanced stages may provide further characterization of human interneuron maturation.
This in-depth explanation was not provided in the initial manuscript because of space constraints as well as clarity. We are happy to include this explanation in this rebuttal to the reviewer to incorporate the reviewer’s suggestion.

> 1.4. It would be necessary to control for the expression of the GFP reporter in combination with in situ hybridisation. I.e. that no interneuron population is missed because of the lack of Dlx gene expression.

We appreciate the reviewer’s comment regarding the expression of the DLXi56-eGFP reporter and its validation. As we perform a thorough characterization of the

identified GFP+ cells using immunostaining analysis and scRNA-seq, which shows the GABAergic nature of the identified GFP+ interneurons and identifies the major subtypes of cortical interneurons, we believe that the current analysis provides clear evidence about the specificity of the GFP reporter. We agree with the reviewer that we cannot completely assume that every interneuron population is captured by the reporter. However, we do see expression of LHX8+ cholinergic interneurons and striatal neurons, indicating a broad coverage of GABAergic cell types labeled by the receptor. Furthermore, the goal of analyzing interneuron migration and tracking migrating cortical interneurons does not require the exhaustive coverage of every rare subtype, but instead a consistent and well characterized reporter so that the experimenter has a transparent understanding of the identity of the cells under observation. Therefore, we believe that the DLXi56-eGFP reporter as characterized and presented in this study is suitable for identification and analysis of cortical interneuron development. We agree with the reviewer that their point about the precision of the DLXi56 reporter should be mentioned, and therefore have added this clarification in the results section discussing the DLXi56-eGFP reporter (lines 186-192 and lines 563-571).

> 1.5. When the authors discuss the presence of strital interneuron identities in ventral organoids, do the cells that cannot route to the striatum will be eliminated or instead continue to aggregate at a ventral node? Given this physical barrier, do stratal interneurons reroute to cortical sites (aberrant migration) upon pharmacological challenges (i.e. cannabinoid and glutamate receptor KOs exhibit such phenotypes).

As identified by the scRNA-seq analysis in Figure 2D, we observe that striatal interneurons and MSNs are predominantly present in the ventral regions of fusions, corresponding to their position within the developing neostriatum (Tepper & Bolam, 2004; Bolam *et al*, 2000). In contrast, the live-imaging experiments to assess acute migration dynamics do not allow discrimination of molecular identity which would be required for GABAergic subtype identification (i.e. striatal versus cortical interneurons). Therefore, it is not possible to determine whether striatal interneurons migrate into the cortex when a pharmacological treatment was applied. This may be determined from longer term treatments, however with the large caveat of non-specific effects due to long-term pharmacological perturbation. Therefore, although we find the reviewer’s hypothesis very intriguing, we believe this is beyond the scope of our study and assay system.

> 1.6. One would expect that organoids cease the production of new neurons if they recapitulate human development. Does this occur after 90 days? Is there a reduction in proliferative potential between 70 and 90 days? If not, one could hypothesize that enrichment in neuronal subclasses is a stochastic process and unrelated to what is reported in post-morteum tissues.

We appreciate the reviewer’s comment regarding the production of new neurons in organoids, however in contrast to mouse brain development, human interneurons have been reported to migrate into the cortex even throughout the first year of postnatal life (Arshad *et al*, 2016; Paredes *et al*, 2016a).In addition, proliferative cells have been observed in human ganglionic eminence regions until ˜28 weeks (˜200 days) (Bigio, 2011). Therefore, we would not expect a reduction in proliferative

potential in human ventral forebrain organoid tissue. Furthermore, the human organoid fusion systems appear to recapitulate this timing of human fetal development, with cortical interneurons (particularly from the CGE) (Hodge *et al*, 2019) being generated late and maturing well into postnatal stages (Nicholas *et al*, 2013). This explanation is now added to the manuscript in the results section of the scRNA-seq analysis (Lines 235-238).

> 1.7. On page 7, it would be helpful to add a sentence and reference to papers from eg the Kriegstein lab on receptor expression in prenatal interneurons in vivo. Receptor profiling of human interneurons during development has been the subject of earlier studies, which should be mentioned.

We thank the reviewer for their important comment about previous studies profiling the neurotransmitter profile of both excitatory and inhibitory neurons in the developing human cortex. In particular, these studies show AMPA-sensitivity of particularly MGE-born interneurons using calcium-imaging and transcriptomic data, which is important for the overall understanding of the migration assay we perform. The relevant references such as (Mayer *et al*, 2019) have been added to the discussion of glutamate receptor modulation in our study (lines 416-419).

> 1.8. For TrackPal, it would be great to have direct comparison on its precision in perhaps a simpler system, e.g. IncuCyte scratch-wound assay or similar and migrating human neurons in 2D or 3D.

We welcome the reviewer's point about the need for an assessment of TrackPal's precision. TrackPal combines new and classical track descriptors designed for the analysis of complex motion. For this task it is therefore not clear how a performance criteria or benchmark could be defined. The use of a simpler system such as IncuCyte or other 2D cells such as leukocytes would enable the assessment of the tracking of the moving cell itself. In our setup, the actual tracking was performed with the commercially available software Imaris (Bitplane AG). The quality of Imaris tracking has recently been compared to other freely available and commercial tools such as Icy and Phagosight (Mitchell *et al*, 2020), indicating its high efficiency in tracking cell migration. We expand upon the already efficient Imaris system by enhancing the tracking with pre-segmentation of the interneuron cell body before tracking in order to differentiate the cell body from the leading process more cleanly. This overcomes the issue of the leading process being recognized as the cell body without proper segmentation and enables correct interneuron tracking. Overall, we believe the accuracy of Imaris, supplemented by Ilastik tracking, is supported by the available data and a comment about the precision of Imaris tracking is now added to the methods section (Lines 837-838).

> 1.9. Reference to papers from the Ben-Ari lab on interneuron migration vs. GABA and glutamate receptor function in mouse cortex should be given. Even if mouse work, yet most of the phenotypic changes that are described here are known, and hence credit should be given where it is due.

We thank the reviewer for this comment and absolutely agree that particularly the function of GABA and glutamate receptors in the mouse cortex has been well

studied and reviewed by Luhmann and colleagues (Luhmann *et al*, 2015). The studies by Manent and colleagues from the Ben-Ari lab (Manent *et al*, 2005, 2006; Manent & Represa, 2007) looking at hippocampal and cortical interneurons in mouse brains and their sensitivity to glutamate and GABA signaling modulation are seminal in our understanding of early interneuron modulation and activity. These relevant references have now been added to the results section discussing the effects of glutamate and GABA modulation (Lines 388-391).

> 1.10. One wonders why interneurons intrinsically express 5HT2C receptors if 5HT is not available. How much is the 5HT receptor expression pattern biased by the model (i.e. comparison to human fetal expression patterns shall be presented). 5HTR2C mediates excitatory transmission. It is coded on the X chromosome in humans - i.e. does the gender identity of the organoid affect expression and/or function/responses? 5HTR2C receptors seem to modulate eg dopamine release by being presynaptic. Are these receptors in e.g. the growth cones of GABA interneurons for chemotropic guidance and subsequently at presynaptic locations once the cells become stationary? Can one elicit directional migration by serotonin in organoids (as of "fewer directional changes")? It would be particularly interesting if these interneurons were sensitive to antidepressant action (i.e. dose-dependent antidepressant action at a subthreshold extracellular 5HT concentration), which could significantly increase the novelty value of the paper.

We agree with the reviewer's assessment and also find it interesting that interneurons intrinsically express serotonin receptors. As we explained in the manuscript (Lines 447-455), serotonergic fibers originate from the midbrain and are not present in our system of the human cortex. However, interneurons even in the cortex express serotoninergic receptors (Frazer *et al*, 2017). In mouse studies, serotonin can alter interneuron migration (Riccio *et al*, 2009; Murthy *et al*, 2014), however this has not been tested in human neurons. Therefore, we show that receptors are transcriptionally present, and that migrating human interneurons can also functionally respond to activation of serotoninergic receptors.

The organoid tissue is generated only from female hESCs, therefore we cannot provide insight into gender-specificity. Although gender-specificity is an interesting topic, we believe this is beyond the scope of the current study.

We do not have any information on the subcellular localization of neurotransmitter receptors. We now include immunofluorescent staining for the neurotransmitter receptors in Supplementary Figure 6B-G to gain some insight on the localization of these receptors. We further observe the expression of the neurotransmitter receptor genes in RNA-sequencing data of GFP+ cells from fusions (Figure S6A), which validates our scRNAseq analysis (Lines 300-306). However, it is important to note that the presence of a channel protein does not prove it is functionally inserted correctly in the cellular membrane and makes further conclusions difficult. Moreover, the live-imaging analysis cannot be combined with molecular techniques. Therefore, the subcellular visualization of a receptor cannot be linked with migratory (i.e. moving or stationary) status. This is certainly an interesting question, but it is beyond the scope of this study.

We are also interested in the effect of antidepressants on interneuron migration. However, most antidepressants such as SSRI and tricyclic antidepressants act on the serotoninergic projections from the midbrain by blocking serotonin reuptake transporters (SERT). This results in larger concentrations of serotonin within the synaptic cleft, which signals through the receptors on migrating interneurons and other cells. Therefore, as we explained in the original manuscript (Lines 447-455), the application of serotonin may mimic the end result of increased serotonin, which could be caused by antidepressants. Thus, we believe our data provides some of the insight that the reviewer requests.

> 1.11. Similarly, the authors mention cannabinoid receptors: why were these not tested functionally? Cannabinoid receptors are selectively on CCK interneurons (i.e. CGE-derived/5HT3A interneurons), and will induce aberrant migration and growth cone retraction. Showing selectivity to THC or a prototypic CB1R ligand (WIN55,212-2 or HU210 could work well) could again be of significant functional value.

We thank the reviewer for this very valuable suggestion. In our dataset, we do not find CCK+ interneurons and in our drug treatment study focused on broader classes of interneurons. Nevertheless, we agree that testing the role of cannabinoid receptors would be an interesting additional experiment to include in our manuscript. Adding this experiment, however, would need at least half a year as we need to age the organoids to the appropriate stage. We admit that this slow time scale is a major drawback of our system. Nonetheless this would be of interest to test in a follow up study. For this manuscript, however, we feel that it would not change the major conclusions.

> 1.12. what are the mechanistic underpinnings of the different modes of migration? Can one migratory mode transit into the other (e.g. upon disruption for cytoskeleton or pharmacological challenge?).

We welcome the reviewer’s comments regarding the modes of migration and whether they can switch and are equally intrigued by this possibility. However, with the current implementation of TrackPal each cell track is assigned to one mode of migration, since we set out to understand whether quantitative analysis of interneuron migration could provide insight into the modes exhibited by migrating interneurons. The track parameters derived by TrackPal describe a single neuron for its entire observation period. If a neuron changes its migration mode over time, this leads to a continuous overlap of the modes, and that is actually what we observe. The analysis of the migration data would need to be tailored specifically for the goal suggested by the reviewer, which despite its interest to us was not suitable for the current goal of this study.

> In sum, this is a very well elaborated paper. Yet increasing its value in terms of novel discovery and mechanistic impact can be seen as a must for it to be out of a "niche" modelling paper.

Referee #2:

This manuscript describes an advanced method to generate hESC-derived assembloids where the ventral and dorsal forebrain patterning steps are improved to generate cells that better recapitulate features of cortical interneurons after fusion. Moreover, the authors report a novel automatized tracking tool to assess cell migration, thereby showing that cortical interneurons navigating into the dorsalized regions of the assembloid (which is devoid of tissue organization) tend to show moving behavior reminiscent of what has been described in the mouse cortical wall (guided, exploratory/dispersed, and more confined migration). These observations are interesting and suggest that an important part of the multimodal migration of cortical interneurons is cell-intrinsically programmed. The work also focuses on the role played by some metabotropic and ligand-gated ion channel receptors activated by neurotransmitters during migration. This has previously been described in rodent, but never in hESC-derived interneurons. Overall, the manuscript provides a new set of biological and analysis tools that will be of great interest for neuro- an cell biologists. However, while there is no doubt that the data have been carefully generated, the manuscript in its present form reports interesting scientific observations that need further validation. Moreover, this work fails short in providing a mechanistic understanding of how distinct neurotransmitters impact differently cortical interneuron migration in assembloids.

Major comments

2.1. Regarding Figure 1 and the advanced method to generate assembloids. Since organoids have been generated via a new method, the dorsal and extend ventral organoids need further analysis (proportion of cycling cells, survival...). The reviewer also suggests to better match the name of dorsal organoids generated classically or by the improved method (as this is the case for the ventral ones) to help the reading of non-specialists. The extended ventral protocol seems to present more ventricle as compared to control, is that is significative? (Fig S1A-D). It it surprising that ventrally and dorsally patterned organoids are fused at such unequal size (see fig 1D). This has not yet been reported (Sloan 2018, Birey 2017, Bagley 2017) and should be clearly explained if it represents and additional step of the improved protocol.

We thank the reviewer for their comment pertaining to the organoid method. As suggested by the reviewer, we have included qPCR data regarding the expression of classical markers of forebrain development (FOXG1), dorsal forebrain (PAX6 and EMX2) and ventral forebrain (NKX2-1) for the new protocols in the manuscript (Figure 1B, S1A), indicating correct generation of the required tissue.

We agree with the reviewer’s comment that the ventricular zones within the depicted organoid generated using the extended ventral protocol seem more pronounced (Figure S1). It is unclear whether larger ventricular zones within ventral organoids may be indicative of better differentiation. We therefore have now performed quantification of the size of the ventricular rosettes for extended ventral and ventral organoids and included this in Supplementary Figure1E. Here, we can observe no

significant difference between the ventricular zone area within extended ventral protocol when compared to the ventral protocol. Furthermore, we address this by immunostaining and qPCR analysis for medial ganglionic eminence marker NKX2-1 (Fig. 1B, S1C), which indicated proper ventral differentiation.
We agree that simplifying the naming of the improved and classical protocols will enhance the comprehension of Fig. 1A,B and have changed this in an improved manuscript version. The classical protocols are now simply labeled Dorsal (D) and Short Ventral (SV) and the new protocols are simply labeled Dorsal + CHIR (D+) and Extended Ventral (EV). We hope this simplification enables easier comprehension of our analysis.

Ventral organoids are consistently shown to be smaller in size to their dorsal counterparts in previous studies (Sloan *et al*, 2018, Figure 4B) (Xiang *et al*, 2017, Figure 7A). The prolonged duration of ventral forebrain induction in our extended ventral protocol may lead to smaller sizes than has been observed previously. On the other hand, it has been previously shown (Lancaster *et al*, 2017) that CHIR treatment leads to a rapid expansion of cortical plates within neural rosettes. This explains the larger size of CHIR-treated dorsal organoids than has been observed elsewhere (Birey *et al*, 2017; Velasco *et al*, 2019). We include a quantification for the rosette areas as Supplementary Figure 1E, revealing significantly larger rosette areas for organoids generated using the ED protocol, affirming the expansion of these areas due to CHIR treatment (lines 139-142).
Therefore, the combination of these two adapted protocols leads to generation of ventral forebrain organoids that are relatively smaller than dorsal forebrain organoids, causing fusions of organoids at an unequal size. We have furthermore added this vital point to the methods section discussing the protocols (lines 719-724) and completely agree with the reviewer that this should be noted in the manuscript.

> 2.2. The figure 1F show that the dorsal region is full of GFP-negative cells that express GABA, what are they?

As the reviewer correctly identifies, there are indeed GABA+ cells in dorsal regions that are not GFP+ and hence generated in the dorsal regions of the fusion. These are interneurons generated from limited CGE/LGE regions within the dorsal organoid, which are present due to the incomplete induction of pure dorsal fate within dorsal organoids generated using CHIR addition. This is consistent with previous studies indicating that few interneurons can still be produced in dorsally-patterned organoids (Lancaster *et al*, 2017; Giandomenico *et al*, 2019). Furthermore, we added data from a new RNA-sequencing experiment as Supplementary Figure 2 which further illustrates the presence of these interneurons within dorsal organoids (cluster 7, Figure S2D). A specific comment to this point is now added to the results section for Figure 1 discussing this population of GABAergic cells within dorsal tissue (Lines 182-185).

> 2.3. In Figure 2E, the number of striatal neurons is higher in 70-days old organoids (127) as compared to 90-days old (34). This suggests a temporal bias of generation of these neurons in organoids and this should be discussed.

We thank the reviewer for this comment regarding the number of striatal neurons and a possible age bias observed in our scRNA-seq analysis. These cells referred to as LGE-Y2 (Figure 2E) could be a specific type of striatal cell with an early temporal generation within ventral organoids. We completely agree with the reviewer that this intriguing observation has not been discussed in the manuscript and we have included it in the results section for the scRNA-seq analysis (Lines 244-249).

> 2.4. The data presented in figure 3A and 3B are unclear and need to be better explained and represented for non-specialists.

We thank the reviewer for their comment and changed the description of this data within the main manuscript to explain the pseudotemporal analysis presented in Fig. 3A and 3B in further detail. We specifically focused on explaining the exclusion of non-cortical cells (Lines 261-268). Moreover, we included information regarding the number of cells used for analysis in the figure legend.

> 2.5. The analysis of the scRNAseq data obtained from the assembloids provide the demonstration that human-derived cortical interneurons express bona fide markers as well as an important repertoire of neurotransmitters receptors with dynamic expression across maturation stages. These data need further validation by qRT-PCR and immunostainings (when ab are available). Moreover, there is no functional demonstration (electrophysiology, calcium imaging..) that these receptors can be pharmacologically activated/ blocked and there is no clue provided about the source of neurotransmitters in assembloids.

We welcome the comment of the reviewer highlighting the necessity of further validating the expression of the identified receptors. We now performed immunostainings of some of the identified receptors in fusion tissue where possible (GABRA1, GRIA2/3, GRIK2, NMDAR1, HTR2C and GLYR) and included it in Supplementary Figure 6B-G. We further perform RNA sequencing of cells from both GFP+ and GFP- populations from both ventral and dorsal regions (Figure S2) to identify the expression of neurotransmitter expression in bulk tissue (Figure S6A) to validate this result. This enhances the information gained by providing further evidence for the presence of the observed receptors within organoid fusions (lines 300-306).
We further agree with the reviewer that functional characterization of the receptor function is essential, and this was indeed the purpose of the live-imaging migration assay. Since the actions of these neurotransmitters in migrating cells is non-synaptic because migrating cells have not formed synaptic connections, an electrophysiological characterization (via calcium imaging or patch clamp) of receptor function will not assess the role of the signaling pathway on migration. Moreover, we would need to perform electrophysiology after assessing the migratory behavior of a cells. This highly complicated and challenging experiment would yield a low rate of success resulting in analysis of very few cells which cannot be adequately compared to the thousands of cells analyzed for migration. We find the experiment very intriguing but feel this would represent an entirely new stand-alone study.
We agree with the reviewer that the rationale regarding the source of neurotransmitters within organoid tissue has not been elucidated in detail. GABA is produced by GABAergic interneurons, which are produced in ventral organoids and

are labeled by the DLXi56-eGFP reporter and are characterized in Figures 1-2 (Lines 381-382). Glutamate is produced by pyramidal neurons, whose presence is determined using RNA sequencing (cluster 7, Figure S2D) (Lines 416-419) . Glycine is present in the culture media of cerebral organoids (Lancaster *et al*, 2017) (Lines 433-435). Serotonin is not produced in cerebral organoid fusions as these do not contain the serotonergic fibers that release serotonin (noted in Lines 447-455). Therefore, we inhibit GABA, glutamate and glycine receptors and add serotonin to stimulate the serotonin receptors in the live-imaging analysis (Figure 5B).

> 2.6. The figure 5 is very dense and not easy to understand at first look. This is particularly the case when trying to compare figure 5C and D. How can you extract simple effect (green and red arrows/lines) of the activation of neurotransmitter receptor (which is indeed not always demonstrated as blockers and not neurotransmitters are used in the culture medium) as represented in figure 5D when blockers impact so many migration parameters, as shown on the heatmap of figure 5C?

We agree with the reviewer's assessment that Fig. 5 is very dense when relating to the multitude of effects observed in Fig. 5C and S7A. As correctly identified by the reviewer, the fact that inhibitors of receptors are used to identify the effect of neurotransmitters further complicates comprehension. We clarified these results by integrating information about the action of the pharmacological treatment applied to Fig. 5D and directly show the results of the drug modulation. Moreover, we highlight the changes more effectively by explaining the arrows in Figure 5D in the figure legend and adding information on which parameters were used to identify these changes. Now, Figure 5D is a direct continuation of Figure 5C and S7A and lends itself to easier comprehension.

> 2.7. The blockade of glutamate receptors alters the directionality of movement of interneurons. It would be interesting to test whether GluRs are indeed necessary to initiate migration from ventral to dorsal regions (AP5/CNQX treatment before migration initiation at 40-50 days). Moreover, along this line, it would be great to know whether the inhibition of GABA-rho and GluRs at early time points (40-50 days) alters the density of migrating interneurons in the dorsal part (proximal vs distal area to ventral area).

We thank the reviewer for this comment and agree that early modulation of such receptors and their effect on distribution of migrating interneurons within fusions is indeed an intriguing possibility. However, the proliferation and migration of interneurons is continuous over many months, especially in the human cortex (Arshad *et al*, 2016). Short application of a pharmacological treatment may impact only a small wave of interneurons. Since our reporter does not include the possibility for pulse-labeling, this small effect will be diluted in quantification of overall numbers of interneurons after multiple weeks. Therefore, we do not feel our system, which focuses on acute receptor modulation and changes in single-cell migration behavior, can accurately assess the reviewer's suggestion. Moreover, the long-term pharmacological treatments could also cause other non-specific effects, which are minimized using the current experimental setup. Overall, the development of an additional genetic tool to allow pulse-labeling experiments would be an intriguing follow up study.

2.8. While the author suggest that the pharmacological inhibition of distinct neurotransmitter receptors alters the migration of interneurons in organoids, they haven't addressed the underlying mechanisms. Moreover, it is unclear whether these changes are strictly cell autonomous as cortical interneurons migrate between other dorsal cells that are likely to express neurotransmitter receptors. Thus, it is necessary to test whether KD neurotransmitters receptors directly in interneurons (shRNA/miRNA/CrispR) affect their migration in dorsal area of assembloids.

We thank the reviewer for their intriguing comment. Among the different mechanisms of migration guidance, chemotactic guidance seems to be the predominant form of migration guidance cortical interneurons are subjected to (Flames *et al*, 2004). Therefore, for this study, we chose to specifically target these receptors by blocking their transmission. However, we agree with the possibility that there may be other non-cell autonomous mechanisms (in particular heterotypic interaction with pyramidal cells) influencing the observed migration effects. Addressing this concern requires performing a genetic screen to analyze the effects of KD of the various receptors identified in this study. This would involve development of additional genetic tools which is very interesting, but we believe goes beyond the scope of this study and could serve as a stand-alone future study. Based on the reviewer's comments, we have added a point about cell-autonomous and non-autonomous effects as possible alternative mechanisms in the discussion section, as this is an important clarification of our current experimental setup (Lines 636-645).

2.9. The figure 6 nicely shows the multimodal migration mode adopted by cortical interneurons. Can authors correlates this observation with position in the dorsal areas of assembloids (proximal vs distal to the ventral area).

We absolutely agree with the reviewer that the matching of the mode exhibited by an interneuron to its proximity to ventral or dorsal regions is intriguing, since one could expect a bias towards directed motion in more ventrally located areas and a bias towards confined motion in more dorsal regions. However, as we noted in the discussion of these modes, the lack of a stereotypical organization of the ventricular zones and developing cortical layers with the fusion is the exact reason why such an analysis is challenging in the organoid system. This is also one of the major points that intrigued us to explore whether we could identify modes based solely on quantitative measurements of migration itself and thereby alleviate the lack of a stereotypical organ structure which is essential in analyses previously performed in mice studies. Therefore, we believe that our analysis of migration addresses this intriguing remark by the reviewer in an indirect but quantitatively assessed manner. This important point has now been clarified and highlighted in the discussion section (lines 612-622).

Specific comments

2.10. Line 120-129 and Fig 1 A: The authors should provide a better description of the protocols used and make them easy- to read for non-specialists

We thank the reviewer for this comment and have added Supplementary Table 1, which provides a detailed description of the protocols and the media used to make them easier to follow.

> 2.11. Fig 5D: Not legend about arrows, etc.

We appreciate the reviewer's comment regarding the lack of explanation of the arrows in Fig. 5D in the figure legend and have added this information in the figure legend.

> 2.12. Lines 475-476: inhibition of GABAB signaling "reduced" the proportion of cells demonstrating CM should be replaced by "increased"

We thank the reviewer for this comment and have made the requested changes to the manuscript (lines 524-525).

Referee #3:

In this manuscript by Bajaj and colleagues, the authors postulate that different neurotransmitter signaling pathways impact on the interneuron migration mode. They introduce an improved patterning protocol used prior to fusion of dorsally and ventrally pre-patterned brain organoids. To allow cell-type specific tracing of migrated interneurons within dorsal structures of fused organoids, they generate a transgenic reporter (DLXi56-GFP) pluripotent stem cell line. scRNA-seq was then used to characterize interneurons and in particular the migrated cells on a molecular level. Transcriptomic characterization of GFP-positive FAC sorted cells revealed the existence of all major human forebrain GABAergic populations. Next, the authors put a particular focus on migrating interneurons by pseudotemporal reconstruction of development trajectories. Increasing expression of several neurotransmitter receptor genes correlating with the degree of maturation was observed. In the second part of the study the authors aimed at studying how neurotransmitter signaling would impact on interneuron migration behaviors. Therefore, they developed an image analysis software package to study neuronal migration by implementing 48 different track parameters. This was used to analyze about 4000 interneurons migration tracks including the ones of neurotransmitter treated cells and revealed distinct alterations of neurotransmitter-treated cell migration modes. Unfortunately the final analyses need some refinement.
In sum, this is a very interesting study which allows detailed dissection of interneuron migration modes by applying TrackPal to the organoid fusion system. However, I have some concerns, suggestions, and comments as specified below.

Major points

3.1. On d70 and d90 fused organoids were dissected to perform scRNA-seq of the ventral and dorsal regions. Given the example picture in Fig. 1E and the fact that there are many GFP cells in the previously dorsally patterned organoids, I wonder whether GFP expression is accurate enough. Or is it the size difference that is used? Can the authors provide a picture and/or comment on this aspect? What is the fraction of GFP-positive cells in the dorsal parts?

We thank the reviewer for their comment regarding the GFP+ cells in the dorsally patterned organoids. Figure panels 1D and 1E display the growth of the fusions of ventrally-patterned organoids generated from the DLX56-eGFP cell line and unlabeled dorsal organoids at Day 20 of the fusion protocol. At day 40, only the ventral organoids show GFP expression, representing the generation of GFP+ interneurons in this region (Figure 1D). At 60 days of age, GFP+ cells migrate from the ventral organoid regions into the unlabeled dorsal organoids (Figure 1E). The discrimination between dorsal and ventral regions is a combination of size and number of GFP+ cells. There is an obvious size difference as well as a clear distinction based on brightness of GFP. Both of these aspects are shown in the figures 1D-E. We also now made this more clear in the methods section (lines 719 - 724). Furthermore, images of dissected organoids showing the differences in size

and GFP intensity between the two regions have been included in the manuscript in Supplementary Figure 2B.

In Figure S2C, we show a representative FACS plot displaying the proportion of GFP+ cells obtained from both the ventral and the dorsal regions of dissected fusions. In this dissection, 1.6% of sorted cells in dorsal regions and approximately 30% of sorted cells in ventral regions were GFP+. Across the dissections performed by us, the proportion of GFP+ cells ranged between 1-7% for dorsal regions and 30-50% for ventral regions. This information has been emphasized in Figure S2C (green labeling) and added to the figure legends .

> 3.2. To me it is surprising and rather counterintuitive that there are more progenitors in the d90 organoids than in the d70 ventral regions (Fig. 1E). Can the authors comment on this?

We appreciate the reviewer's comment regarding the number of progenitors in the cells obtained from fusions at day 70 and 90. The reviewer probably refers to Fig. 2E, which details the number of cells used for scRNA-seq analysis and we observe a higher number of progenitors obtained from 90-day old fusions in our scRNA-seq analysis. As the reviewer correctly suggests, this is rather counterintuitive, as one would expect older, mature organoids to contain a higher proportion of mature interneurons and correspondingly lower numbers of progenitors. However, our data in figure panels 2D and 2E show that the major determinant of interneuron maturation was not the age of the organoid (both day 70 and day 90 old fusions contained progenitors, young and mature interneurons) but rather the localization of cells in either the ventral or dorsal region (ventral regions contained higher proportion of progenitors and young neurons whereas dorsal regions contained a higher proportion of mature neurons). This observation that age of the organoid alone cannot be used to accurately assess maturation thus prompted us to perform the scRNA-seq analysis across both ventral and dorsal regions. Only then could we assess the importance of the spatial information in understanding the maturation of cortical interneurons. We incorporated the comments of the reviewer in further detail in the results section discussing the scRNA-seq analysis (lines 233-238; 252-258). In addition, proliferative cells have been observed in human ganglionic eminence regions until ˜28 weeks (˜200 days) (Bigio, 2011). Therefore, we would not expect a reduction in proliferative potential in human ventral forebrain organoid tissue. This explanation is now added to the manuscript in the results section of the scRNA-seq analysis (Lines 233-238).

> 3.3. The authors state in the discussion that the maturation is migration-dependent. There is no evidence shown that the maturation is indeed dependent on the migration. Are there no comparably mature interneurons in the ventral part? In this context it would be good to see how corresponding interneuron transcriptomes look like without fusion?

We agree with the reviewer's comment that no evidence is shown that maturation is dependent on migration and the term "migration-dependent maturation" is misleading. We show that GFP+ interneurons in the ventral regions of fusions are predominantly progenitors and young neurons, while more mature interneurons are

predominantly observed in dorsal regions of the fusions (Figure 2D). We do not determine whether it is the process of migration itself or changes interneurons undergo after migration has subsided, that drive their maturation. This point is not abundantly clear from the current version of the manuscript and is now clarified in the discussion (Lines 572-580), and we thank the reviewer for suggesting this clarification.

To address the intriguing question raised by the reviewer regarding the comparison of the maturity of transcriptomes of ventrally located GFP+ interneurons in single ventral organoids (non-fused), ventrally located GFP+ interneurons in fused organoids and dorsally located GFP+ interneurons in fused organoids, we have included an RNA sequencing experiment as Supplementary Figure 2. Here, we show that GFP+ interneurons which have migrated into dorsal regions (cluster 3, Figure S2D) show a higher relative expression of mature interneuron markers such as SST, PVALB and TH in comparison to GFP+ interneurons in the ventral regions (both fusions and non-fused single organoids; cluster 1 (partly) and cluster 2,Figure S2D). This confirms that interneurons in dorsal regions are more mature than ventrally located interneurons which have not migrated into dorsal regions, thus affirming the results of the scRNA-seq analysis (Figure 2).

> 3.4. The authors performed pseudotemporal analyses using all cells introducing that they want to focus on the cells that migrated. I wonder whether it wouldn´t be advisable to selectively focus on the GFP+ cells isolated from the dorsal regions since these had been migrated there. If they did then it was not clear. Also, I did not find how many cells were included in the pseudotemporal analysis (see minor comments below).

We perform pseudotemporal analysis using all cells in the scRNA-seq analysis. This is based on the previous analysis indicating that a maturation spectrum is observed according to spatial location (i.e. across the dorsal-ventral axis). Since the pseudotemporal analysis aimed to identify the entire developmental trajectory of interneurons from their generation to their maturation, we used GFP+ cells from both ventral and dorsal regions. Using only GFP+ cells from dorsal regions would exclude the younger cells and make reconstruction of a developmental trajectory difficult. This point was clarified in the results section for Figure 3 (lines 261-268). In addition, the exact number of cells used for the pseudotemporal analysis is now included in the figure legend. We thank the reviewer for these constructive suggestions.

> 3.5. Concerning the clustering approach to classify different migratory behaviors in Fig. 6. According to the methods section, the authors performed first PCA analysis and clustering of 696 control interneurons and then assigned the closest control cluster ID to the treated interneurons. In the main text however, they mention that all tracked interneurons were utilized to increase the robustness of the clustering. The authors should clarify this. Why not cluster all (tracks of non-treated and treated cells) together? What if there is completely new migration mode?

We thank the reviewer for emphasizing the approach to the clustering of migration tracks. As the reviewer correctly identifies from the methods section, in the current version of the manuscript, we only used control tracks for PCA and clustering

analysis. This was done in order to first determine and characterize “control” migration modes without the modulation of the neurotransmitter receptors. Then, we assigned a cluster ID to all treated interneuron migration tracks, because we expected changes in the composition of the migration modes. However, as the reviewer notes, this analysis cannot exclude the possibility that a completely new migration mode may not be detected. Hence, we performed PCA and cluster analysis on all tracks (regardless of their treatment status) and compared it to the results of the original control classification method. These data are now included in a new Supplementary Figure 12. We observe that the PCA derived from control tracks only is nearly identical to PCA derived from all tracks. We visualized this by showing the first 6 principal components (covering ~80% of variance explained) for the two PCA distinct derivations. Comparing the clustering achieved by the two methods also reveals the same relative migration modes and only a slight change in the composition (i.e. number of tracks assigned to each cluster). Overall, the reviewer’s important comment further strengthens the robustness of our analysis, and we thank them for this helpful suggestion. We now highlight this in the manuscript as well (Lines 479-484).

> 3.6. The authors provide a mathematical framework to group the migration modes into 9 clusters. However, the higher-order groups (directed, explanatory, confined) are hand-picked and not based on an unbiased classification. This requires a clearly defined, unbiased, and reproducible approach. It is essential, in particular because the effect of the neurotransmitter treatments on the migration modes is based on this higher-order groupings.

We thank the reviewer for this remark and agree with their assessment of the tracks. We initially performed an unbiased analysis on all cells to determine the existence of the different modes and decided to deviate from the unbiased “super-clustering” based on specific observations related to certain tracks, which we clarify here. After k-means clustering stratified the migration tracks into 10 different clusters (Figure 5A, S9) based on different categories of parameters (speed, shape, pausing and direction), we applied hierarchical k-means again by clustering the 10 parameter-averaged migration modes into 3 “super-clusters”. This is now included as a new Supplementary Figure 13. Using this unbiased clustering, we observed that the original clusters 0-3 were assigned to super-cluster 1, cluster 4 was assigned to super-cluster 2 and clusters 5-9 were assigned to super-cluster 3. We observed that cluster 4 was a highly variable cluster with high parameter variance (high values for speed and shape, but also pausing) and thus differed from the other clusters. However, due to this high variance of cluster 4, clusters 5-9 were all grouped into a single “super cluster”. This differs from our current biased analysis where clusters 4-6 are assigned to super-cluster 2 and clusters 7-9 are assigned to super-cluster 3 (see Supplementary Figure). Now, when we examine the values for the parameter groups within these groups, we clearly see that within this super-cluster 3 clusters 5 and 6 show much higher values for shape parameters (particularly cluster 5) and speed parameters (especially cluster 6), which differentiated them from clusters 7-9. This observation is strengthened by the direct visual representation of these tracks in Figure 6B, where one can see the highly confined nature of clusters 7-9 in comparison to clusters 5 and 6. We therefore concluded that due to the highly

aberrant characteristics exhibited by the tracks in cluster 4, hierarchical k-means could not detect the subtle difference within super-cluster 3. We thank the reviewer for bringing up this point and helping us clarify the prolonged procedure which led to the characterization of the super-clusters or migration modes which we ultimately describe. We included this explanation in the methods section (lines 894-898) about track clustering to provide clarity and transparency in our identification of the modes.

> 3.7. Through the treatments they see shifting of the fractions of directed, exploratory, and confined modes (Fig. 6F). They should include statistics to show that the described effects are meaningful.

We appreciate the reviewer’s comments regarding the statistical analysis to confirm the significance of the results. However, the experimental setup and the data do not allow proper statistical analysis. These ratios are shifts in the fractions (or compositions) of the cluster modes and are computed on the basis of all the data and are as such not “repeated”. The only way of performing statistical analysis would be to repeat the entire set of live-imaging experiments multiple times to gain a suitable number of independent “repetitions”. A theoretical option for analysis would be the bootstrapping method, i.e. repeating the analysis on randomly sampled subsets of the current experimental data. However, such an approach has fundamental problems. Firstly, the violation of independence caused by random sampling of the data into N groups is problematic as the cells assigned to different groups may not be entirely independent, e.g. Images recorded from different slice cultures from the same organoid fusion. Another issue would be the determination of the number of subgroups N. Estimating the mean fractional change in each subgroup requires N to be small enough to contain larger sub-groups, while simultaneously being large enough to assess the variance between the groups. It is not entirely clear how we would make this important determination. Therefore, we thank the reviewer for their comment relating to the missing statistical analysis and have added this to the methods section (lines 899-902) to clarify the current analysis.

> 3.8. Further, I wonder whether they can exclude that shifting towards the confined mode (e.g. through NMDA inhibition) instead means increased maturation. This could be assessed by scRNA-seq of neurotransmitter treated organoids.

We appreciate the reviewer’s insightful comment and agree that the possibility of receptor modulation leading to maturation of interneurons cannot be excluded. This would indeed also change their migration pattern based on the hypothesis that more mature interneurons shift towards more confined modes of migration (Hatanaka *et al*, 2016). However, we would also like to highlight that all live imaging experiments in our analysis consisted of acute drug treatments of organotypic slices of fusions. Therefore, it is unlikely that drug treatments of this duration would lead to strong changes in the maturation status of the interneurons. Nonetheless, the alternative possibility is highly intriguing and is now added to the discussion (lines 631-636).

> Minor points
> 3.9. The authors mention medium change on d3 for the generation of dorsal EBs. This is not indicated in Fig. 1A.

The E8 medium is indeed changed on day 3 of the dorsal EB protocol, however this change involves the replenishment of fresh E8 medium and no change in the actual medium. We agree that this is not entirely clear from Fig. 1A, thank the reviewer for their comment and changed this in the figure to indicate that E8 needs to be replenished at Day 3 of the protocol. We also now include a day-by-day detailed protocol description as Supplementary Table 1 to enable clear comprehension of the protocol steps.

> 3.10. In the box within Fig. 1A it says: Imp+/-A - Differentiation medium. I am assuming that this refers to either improved differentiation medium minus (-) or plus (+) Vitamin A? It is not specified in the methods section that Imp+A is used until d20 but stated that it is added only after fusion and transfer of the organoids on the orbital shaker on d25-26.

The reviewer is correct in observing that the Imp+/- does indeed refer to improved differentiation medium with and without Vitamin A. It is true that Figure 1A does not provide information on the days of the protocol in which Imp+A is added to the fusions and adding it in the legend box is rather confusing for the reader. Hence, we have addressed this issue by adapting the legend in Figure1A and only mentioning Imp-A, which is added until Day 20 (day of the pre-fusion) to avoid further confusion. We thank the reviewer for this helpful suggestion.

> 3.11. It is obvious that the ventrally patterned organoids are smaller in size although they started out with the same cell number. Can the authors comment on this?

The observation that organoids generated under ventral patterning are generally smaller in size compared to other patterning protocols is well known and has been shown to be consistent among many previous studies (Sloan *et al*, 2018, Figure 4B) (Xiang *et al*, 2017, Figure 7A). The prolonged duration of ventral forebrain induction in our extended ventral protocol may further lead to smaller sizes than has been observed previously.
Moreover, it has been previously shown (Lancaster *et al*, 2017) that CHIR treatment leads to a rapid expansion of the cortical plate of neural rosettes within cerebral organoids and hence is responsible for the larger size of CHIR-treated dorsal organoids. We included a quantification of the rosette areas within the organoids as Supplementary Figure 1E, which shows the significantly larger rosette areas in organoids generated using the extended dorsal protocol. This affirms the observation that CHIR treatment leads to larger ventricular zones in organoids and also explains the variation in size between the extended dorsal and extended ventral organoids (lines 719-724).

> 3.12. After some filtering and selection steps, the authors started out from 3635 cells. How many cells are included in the pseudotemporal analysis in Fig. 3A? This should be indicated somewhere, e.g. in the Figure legend.

We thank the reviewer for this important comment and have addressed this issue by adding the specific number of cells used for pseudotemporal analysis in the figure legend for Figure 3.

3.13. I think it would be interesting if the authors would comment on whether the migration behavior could also be studied in a less complex system like directly differentiated neurons in 2D.

We appreciate this comment by the reviewer as it is indeed an important comment that highlights the necessity of the organoid fusion culture system. It has been previously observed (Azzarelli *et al*, 2017; Hernández-Miranda *et al*, 2011) that 2D culture systems do not replicate the natural milieu required for complex migration behaviors exhibited by cells such as cortical interneurons. Migrating cells in 2D cultures generally display a persistent random walk movement (PRW) which is inadequate for the description of “anisotropic” 3D movement (Wu *et al*, 2014). Therefore, 2D cell culture experiments are not sufficient for the analysis of the complex migration patterns exhibited by migrating cortical interneurons and there is a need for the 3D structure provided by cortical organoid fusions.

References

Ahmed NY, Knowles R & Dehorter N (2019) New Insights Into Cholinergic Neuron Diversity. *Front Mol Neurosci* 12: 204

Ang ESBC, Haydar TF, Gluncic V & Rakic P (2003) Four-Dimensional Migratory Coordinates of GABAergic Interneurons in the Developing Mouse Cortex. *J Neurosci* 23: 5805–5815

Arshad A, Vose LR, Vinukonda G, Hu F, Yoshikawa K, Csiszar A, Brumberg JC & Ballabh P (2016) Extended Production of Cortical Interneurons into the Third Trimester of Human Gestation. *Cereb Cortex* 26: 2242–2256

Azzarelli R, Oleari R, Lettieri A, Andre' V & Cariboni A (2017) In Vitro, Ex Vivo and In Vivo Techniques to Study Neuronal Migration in the Developing Cerebral Cortex. *Brain Sci* 7: 48

Bagley JA, Reumann D, Bian S, Lévi-Strauss J & Knoblich JA (2017) Fused cerebral organoids model interactions between brain regions. *Nat Methods* 14: 743–751

Bigio MRD (2011) Cell proliferation in human ganglionic eminence and suppression after prematurity-associated haemorrhage. *Brain* 134: 1344–1361

Birey F, Andersen J, Makinson CD, Islam S, Wei W, Huber N, Fan HC, Metzler KRC, Panagiotakos G, Thom N, *et al* (2017) Assembly of functionally integrated human forebrain spheroids. *Nature* 545: 54–59

Bolam JP, Hanley JJ, Booth PA & Bevan MD (2000) Synaptic organisation of the basal ganglia. *J Anat* 196 (Pt 4): 527–42

Eichmüller OL, Corsini NS, Vértesy Á, Scholl T, Gruber V-E, Peer AM, Chu J, Novatchkova M, Paredes MF, Feucht M, *et al* (2020) Cerebral organoid model reveals excessive proliferation of human caudal late interneuron progenitors in Tuberous Sclerosis Complex. *Biorxiv*: 2020.02.27.967802

Faux C, Rakic S, Andrews W & Britto JM (2012) Neurons on the Move: Migration and Lamination of Cortical Interneurons. *Neurosignals* 20: 168–189

Flames N, Long JE, Garratt AN, Fischer TM, Gassmann M, Birchmeier C, Lai C, Rubenstein JLR & Marín O (2004) Short- and long-range attraction of cortical GABAergic interneurons by neuregulin-1. Neuron 44: 251–61

Frazer S, Prados J, Niquille M, Cadilhac C, Markopoulos F, Gomez L, Tomasello U, Telley L, Holtmaat A, Jabaudon D, *et al* (2017) Transcriptomic and anatomic parcellation of 5-HT3AR expressing cortical interneuron subtypes revealed by single-cell RNA sequencing. *Nat Commun* 8: 14219

Giandomenico SL, Mierau SB, Gibbons GM, Wenger LMD, Masullo L, Sit T, Sutcliffe M, Boulanger J, Tripodi M, Derivery E, *et al* (2019) Cerebral organoids at the air–liquid interface generate diverse nerve tracts with functional output. *Nat Neurosci* 22: 669–679

Gokce O, Stanley GM, Treutlein B, Neff NF, Camp JG, Malenka RC, Rothwell PE, Fuccillo MV, Südhof TC & Quake SR (2016) Cellular Taxonomy of the Mouse Striatum as Revealed by Single-Cell RNA-Seq. *Cell Reports* 16: 1126–1137

Hatanaka Y, Zhu Y, Torigoe M, Kita Y & Murakami F (2016) From migration to settlement: the pathways, migration modes and dynamics of neurons in the developing brain. *Proc Jpn Acad Ser B* 92: 1–19

Hernández-Miranda LR, Cariboni A, Faux C, Ruhrberg C, Cho JH, Cloutier J-F, Eickholt BJ, Parnavelas JG & Andrews WD (2011) Robo1 Regulates Semaphorin Signaling to Guide the Migration of Cortical Interneurons through the Ventral Forebrain. *J Neurosci* 31: 6174–6187

Hodge RD, Bakken TE, Miller JA, Smith KA, Barkan ER, Graybuck LT, Close JL, Long B, Johansen N, Penn O, *et al* (2019) Conserved cell types with divergent features in human versus mouse cortex. *Nature* 573: 61–68

Krienen FM, Goldman M, Zhang Q, Rosario RCH del, Florio M, Machold R, Saunders A, Levandowski K, Zaniewski H, Schuman B, *et al* (2020) Innovations present in the primate interneuron repertoire. *Nature* 586: 262–269

Lancaster MA, Corsini NS, Wolfinger S, Gustafson EH, Phillips AW, Burkard TR, Otani T, Livesey FJ & Knoblich JA (2017) Guided self-organization and cortical plate formation in human brain organoids. *Nat Biotechnol* 35: 659–666

Lancaster MA, Renner M, Martin C-A, Wenzel D, Bicknell LS, Hurles ME, Homfray T, Penninger JM, Jackson AP & Knoblich JA (2013) Cerebral organoids model human brain development and microcephaly. *Nature* 501: 373–379

Luhmann HJ, Fukuda A & Kilb W (2015) Control of cortical neuronal migration by glutamate and GABA. *Front Cell Neurosci* 9: 4

Manent J-B, Demarque M, Jorquera I, Pellegrino C, Ben-Ari Y, Aniksztejn L & Represa A (2005) A Noncanonical Release of GABA and Glutamate Modulates Neuronal Migration. *J Neurosci* 25: 4755–4765

Manent J-B, Jorquera I, Ben-Ari Y, Aniksztejn L & Represa A (2006) Glutamate Acting on AMPA But Not NMDA Receptors Modulates the Migration of Hippocampal Interneurons. *J Neurosci* 26: 5901–5909

Manent J-B & Represa A (2007) Neurotransmitters and Brain Maturation: Early Paracrine Actions of GABA and Glutamate Modulate Neuronal Migration. *Neurosci* 13: 268–279

Marín O & Rubenstein JLR (2003) Cell migration in the forebrain. *Annu Rev Neurosci* 26: 441–483

Mayer C, Hafemeister C, Bandler RC, Machold R, Brito RB, Jaglin X, Allaway K, Butler A, Fishell G & Satija R (2018) Developmental diversification of cortical inhibitory interneurons. *Nature* 555: 457–462

Mayer S, Chen J, Velmeshev D, Mayer A, Eze UC, Bhaduri A, Cunha CE, Jung D, Arjun A, Li E, *et al* (2019) Multimodal Single-Cell Analysis Reveals Physiological Maturation in the Developing Human Neocortex. *Neuron* 102: 143-158.e7

Mitchell C, Caroff L, Solis-Lemus JA, Reyes-Aldasoro CC, Vigilante A, Warburton F, Chaumont F de, Dufour A, Dallongeville S, Olivo-Marin J-C, *et al* (2020) Cell Tracking Profiler – a user-driven analysis framework for evaluating 4D live-cell imaging data. *J Cell Sci* 133: jcs241422

Muñoz-Manchado AB, Gonzales CB, Zeisel A, Munguba H, Bekkouche B, Skene NG, Lönnerberg P, Ryge J, Harris KD, Linnarsson S, *et al* (2018) Diversity of Interneurons in the Dorsal Striatum Revealed by Single-Cell RNA Sequencing and PatchSeq. *Cell Reports* 24: 2179-2190.e7

Murthy S, Niquille M, Hurni N, Limoni G, Frazer S, Chameau P, Hooft JA van, Vitalis T & Dayer A (2014) Serotonin receptor 3A controls interneuron migration into the neocortex. *Nat Commun* 5: 5524

Nicholas CR, Chen J, Tang Y, Southwell DG, Chalmers N, Vogt D, Arnold CM, Chen Y-JJ, Stanley EG, Elefanty AG, *et al* (2013) Functional Maturation of hPSC-Derived Forebrain Interneurons Requires an Extended Timeline and Mimics Human Neural Development. *Cell Stem Cell* 12: 573–586

Paredes MF, James D, Gil-Perotin S, Kim H, Cotter JA, Ng C, Sandoval K, Rowitch DH, Xu D, McQuillen PS, *et al* (2016a) Extensive migration of young neurons into the infant human frontal lobe. *Science* 354: aaf7073

Riccio O, Potter G, Walzer C, Vallet P, Szabó G, Vutskits L, Kiss JZ & Dayer AG (2009) Excess of serotonin affects embryonic interneuron migration through activation of the serotonin receptor 6. *Mol Psychiatr* 14: 280–290

Saunders A, Macosko EZ, Wysoker A, Goldman M, Krienen FM, Rivera H de, Bien E, Baum M, Bortolin L, Wang S, *et al* (2018) Molecular Diversity and Specializations among the Cells of the Adult Mouse Brain. *Cell* 174: 1015-1030.e16

Sloan SA, Andersen J, Pașca AM, Birey F & Pașca SP (2018) Generation and assembly of human brain region–specific three-dimensional cultures. *Nat Protoc* 13: 2062–2085

Tepper JM & Bolam JP (2004) Functional diversity and specificity of neostriatal interneurons. *Curr Opin Neurobiol* 14: 685–692

Velasco S, Kedaigle AJ, Simmons SK, Nash A, Rocha M, Quadrato G, Paulsen B, Nguyen L, Adiconis X, Regev A, *et al* (2019) Individual brain organoids reproducibly form cell diversity of the human cerebral cortex. *Nature* 570: 523–527

Velmeshev D, Chavali M, Nowakowski TJ, Bhade M, Mayer S, Goyal N, Alvarado B, Mancia W, Wang S, Speir M, *et al* (2021) Molecular diversity and lineage commitment of human interneuron progenitors. *Biorxiv*: 2021.05.13.444045

Wu P-H, Giri A, Sun SX & Wirtz D (2014) Three-dimensional cell migration does not follow a random walk. *Proc National Acad Sci* 111: 3949–3954

Xiang Y, Tanaka Y, Patterson B, Kang Y-J, Govindaiah G, Roselaar N, Cakir B, Kim K-Y, Lombroso AP, Hwang S-M, *et al* (2017) Fusion of Regionally Specified hPSC-Derived Organoids Models Human Brain Development and Interneuron Migration. *Cell Stem Cell* 21: 383-398.e7

Zhong S, Zhang S, Fan X, Wu Q, Yan L, Dong J, Zhang H, Li L, Sun L, Pan N, *et al* (2018) A single-cell RNA-seq survey of the developmental landscape of the human prefrontal cortex. *Nature* 555: 524–528

1st Revision - Editorial Decision **21st Sep 2021**

Dear Jurgen,

Thank you for submitting your revised manuscript. Your study has now been seen by the original referees #2 and 3. As you can see from the comments below the referees appreciate the introduced changes and support publication in The EMBO Journal.

I am therefore very pleased to accept the manuscript for publication here. There are just a few editorial points to resolve before I can send you the formal acceptance letter.

Please add 3-5 keywords

"Competing Financial Interests" should be labelled as Conflict of Interest

Please include the funding information in the Acknowledgements.

Please check figure callouts to Fig S9D+K and Fig S11 panels

You have 13 supplementary figures. You can either add them all into an appendix (Appendix Figure S#) or you can make 5 of the EV figures (Figure EV#) and add the rest into the appendix. Please see our guideline to authors for further information and the advantage of EV figures. EV figures need to be uploaded as individual figures while appendix figures need to be uploaded into one PDF file. The appendix needs a a ToC.

Regarding the tables => The supplemental tables should be turned into regular tables, Appendix tables (Appendix Table SXX) or uploaded as Datasets (Dataset EVXX). More complex tables (more than 5 columns or 20 rows) should be uploaded as datasets and the legend should be included in a separate tab and removed from main MS. I would suggest uploading Supplemental tables 1-3 as datasets. For Appendix tables please add legends to appendix and remove them from main MS.

The Movie legends should be removed from the Manuscript file and ZIPed along with each movie. The correct nomenclature is 'Movie EV#'.

You have a supplementary software file - please label the file as Software EV1. And include in the zip folder a README.txt file, with instructions on how to use the software or anything else that is important to mention. Make sure that you have a callout in the text to this file.

I think this should be all. Let me know if you need any help with the files and formats etc. juts let us know.

With best wishes

Karin

Karin Dumstrei, PhD
Senior Editor
The EMBO Journal

Guide For Authors: https://www.embopress.org/page/journal/14602075/authorguide

Please click on the link below to submit the revision.

https://emboj.msubmit.net/cgi-bin/main.plex

Referee #2:

We thank the authors for providing and updated manuscript. Its quality improved significantly and the authors answered experimentally our main concerns and discussed the others.
Please note that there is a remaining typo : line 79 - "develpoment"

Referee #3:

In this revised version the authors have substantially strengthened the manuscript both by the addition of new experimental data and with text modifications. I feel that the implementation of the new RNA-sequencing data further supports the suitability of the system. The combination of a biased, supervised super-clustering with an assessment of the relative contribution to these super-clusters after treatment, that is not tested for its relevance (statistics) remains suboptimal. Other than that, I am fully satisfied with these revisions and support puplication in EMBO Journal.

2nd Revision - Editorial Decision 28th Sep 2021

Dear Jürgen,

Thank you for submitting your revised manuscript to The EMBO Journal. I have now had a chance to look at the revised version and all looks good. I am therefore very pleased to accept the manuscript for publication here.

Congratulations on a nice study!

With best wishes

Karin

Karin Dumstrei, PhD
Senior Editor
The EMBO Journal

Please note that it is EMBO Journal policy for the transcript of the editorial process (containing referee reports and your response letter) to be published as an online supplement to each paper. If you do NOT want this, you will need to inform the Editorial Office via email immediately. More information is available here:
https://www.embopress.org/page/journal/14602075/authorguide#transparentprocess

Your manuscript will be processed for publication in the journal by EMBO Press. Manuscripts in the PDF and electronic editions of The EMBO Journal will be copy edited, and you will be provided with page proofs prior to publication. Please note that supplementary information is not included in the proofs.

Please note that you will be contacted by Wiley Author Services to complete licensing and payment information. The 'Page Charges Authorization Form' is available here: https://www.embopress.org/pb-assets/embo-site/tej_apc.pdf

Should you be planning a Press Release on your article, please get in contact with embojournal@wiley.com as early as possible, in order to coordinate publication and release dates.

If you have any questions, please do not hesitate to call or email the Editorial Office. Thank you for your contribution to The EMBO Journal.

** Click here to be directed to your login page: https://emboj.msubmit.net

EMBO PRESS

YOU MUST COMPLETE ALL CELLS WITH A PINK BACKGROUND

PLEASE NOTE THAT THIS CHECKLIST WILL BE PUBLISHED ALONGSIDE YOUR PAPER

Corresponding Author Name: Jürgen Knoblich	
Journal Submitted to: EMBO Journal	
Manuscript Number: EMBOJ-2021-108714	

USEFUL LINKS FOR COMPLETING THIS FORM

http://www.antibodypedia.com
http://1degreebio.org
http://www.equator-network.org/reporting-guidelines/improving-bioscience-research-repo
http://grants.nih.gov/grants/olaw/olaw.htm
http://www.mrc.ac.uk/Ourresearch/Ethicsresearchguidance/Useofanimals/index.htm
http://ClinicalTrials.gov
http://www.consort-statement.org
http://www.consort-statement.org/checklists/view/32-consort/66-title
http://www.equator-network.org/reporting-guidelines/reporting-recommendations-for-tun
http://datadryad.org
http://figshare.com
http://www.ncbi.nlm.nih.gov/gap
http://www.ebi.ac.uk/ega
http://biomodels.net/
http://biomodels.net/miriam/
http://jjj.biochem.sun.ac.za
https://osp.od.nih.gov/biosafety-biosecurity-and-emerging-biotechnology/
http://www.selectagents.gov/

Reporting Checklist For Life Sciences Articles (Rev. June 2017)

This checklist is used to ensure good reporting standards and to improve the reproducibility of published results. These guidelines are consistent with the Principles and Guidelines for Reporting Preclinical Research issued by the NIH in 2014. Please follow the journal's authorship guidelines in preparing your manuscript.

A- Figures
1. Data
The data shown in figures should satisfy the following conditions:

- the data were obtained and processed according to the field's best practice and are presented to reflect the results of the experiments in an accurate and unbiased manner.
- figure panels include only data points, measurements or observations that can be compared to each other in a scientifically meaningful way.
- graphs include clearly labeled error bars for independent experiments and sample sizes. Unless justified, error bars should not be shown for technical replicates.
- if n< 5, the individual data points from each experiment should be plotted and any statistical test employed should be justified
- Source Data should be included to report the data underlying graphs. Please follow the guidelines set out in the author ship guidelines on Data Presentation.

2. Captions

Each figure caption should contain the following information, for each panel where they are relevant:

- a specification of the experimental system investigated (eg cell line, species name).
- the assay(s) and method(s) used to carry out the reported observations and measurements
- an explicit mention of the biological and chemical entity(ies) that are being measured.
- an explicit mention of the biological and chemical entity(ies) that are altered/varied/perturbed in a controlled manner.

- the exact sample size (n) for each experimental group/condition, given as a number, not a range;
- a description of the sample collection allowing the reader to understand whether the samples represent technical or biological replicates (including how many animals, litters, cultures, etc.).
- a statement of how many times the experiment shown was independently replicated in the laboratory.
- definitions of statistical methods and measures:
  - common tests, such as t-test (please specify whether paired vs. unpaired), simple χ2 tests, Wilcoxon and Mann-Whitney tests, can be unambiguously identified by name only, but more complex techniques should be described in the methods section;
  - are tests one-sided or two-sided?
  - are there adjustments for multiple comparisons?
  - exact statistical test results, e.g., P values = x but not P values < x;
  - definition of 'center values' as median or average;
  - definition of error bars as s.d. or s.e.m.

Any descriptions too long for the figure legend should be included in the methods section and/or with the source data.

In the pink boxes below, please ensure that the answers to the following questions are reported in the manuscript itself. Every question should be answered. If the question is not relevant to your research, please write NA (non applicable). We encourage you to include a specific subsection in the methods section for statistics, reagents, animal models and human subjects.

B- Statistics and general methods

Please fill out these boxes (Do not worry if you cannot see all your text once you press return)

1.a. How was the sample size chosen to ensure adequate power to detect a pre-specified effect size?	No statistical tests were used to pre-determine an effect size or power. The choice of number of repetitions and effect sizes was done based on previous experience and published literature within the organoid field.
1.b. For animal studies, include a statement about sample size estimate even if no statistical methods were used.	The choice of number of repetitions and effect sizes was done based on previous experience and published literature within the organoid field.
2. Describe inclusion/exclusion criteria if samples or animals were excluded from the analysis. Were the criteria pre-established?	No samples were excluded from analysis.
3. Were any steps taken to minimize the effects of subjective bias when allocating animals/samples to treatment (e.g. randomization procedure)? If yes, please describe.	All samples were randomly allocated to either a control or a treamtent group. No selection based on organoid size, quality or otherwise was performed.
For animal studies, include a statement about randomization even if no randomization was used.	All samples were randomly allocated to either a control or a treamtent group. No selection based on organoid size, quality or otherwise was performed.
4.a. Were any steps taken to minimize the effects of subjective bias during group allocation or/and when assessing results (e.g. blinding of the investigator)? If yes please describe.	For all immunohistochemistry analysis the investigators were blinded. The investigators were not blinded for the live imaging analysis.
4.b. For animal studies, include a statement about blinding even if no blinding was done	For all immunohistochemistry analysis the investigators were blinded. The investigators were not blinded for the live imaging analysis.
5. For every figure, are statistical tests justified as appropriate?	All statistical analyses performed are mentioned in the figure legends. The use of statistical tests Is based on the number of groups analyzed and the justification is outlined in the methods section.
Do the data meet the assumptions of the tests (e.g., normal distribution)? Describe any methods used to assess it.	Data was pre-analyzed to confirm whether they are normally distributed and corresponding statistical analysis was performed.

Is there an estimate of variation within each group of data?	Variance between each group of data was calculated and is presented accordingly in the figures.
Is the variance similar between the groups that are being statistically compared?	Variance between each group of data was calculated and is presented accordingly in the figures.

C- Reagents

6. To show that antibodies were profiled for use in the system under study (assay and species), provide a citation, catalog number and/or clone number, supplementary information or reference to an antibody validation profile. e.g., Antibodypedia (see link list at top right), 1DegreeBio (see link list at top right).	Provided in Supplementary Table 6.
7. Identify the source of cell lines and report if they were recently authenticated (e.g., by STR profiling) and tested for mycoplasma contamination.	Mentioned in the Methods section "Stem Cell Culture"

* for all hyperlinks, please see the table at the top right of the document

D- Animal Models

8. Report species, strain, gender, age of animals and genetic modification status where applicable. Please detail housing and husbandry conditions and the source of animals.	Mentioned in the Methods section "Stem Cell Culture"
9. For experiments involving live vertebrates, include a statement of compliance with ethical regulations and identify the committee(s) approving the experiments.	Not applicable
10. We recommend consulting the ARRIVE guidelines (see link list at top right) (PLoS Biol. 8(6), e1000412, 2010) to ensure that other relevant aspects of animal studies are adequately reported. See author guidelines, under 'Reporting Guidelines'. See also: NIH (see link list at top right) and MRC (see link list at top right) recommendations. Please confirm compliance.	Not applicable

E- Human Subjects

11. Identify the committee(s) approving the study protocol.	Not applicable
12. Include a statement confirming that informed consent was obtained from all subjects and that the experiments conformed to the principles set out in the WMA Declaration of Helsinki and the Department of Health and Human Services Belmont Report.	Not applicable
13. For publication of patient photos, include a statement confirming that consent to publish was obtained.	Not applicable
14. Report any restrictions on the availability (and/or on the use) of human data or samples.	Not applicable
15. Report the clinical trial registration number (at ClinicalTrials.gov or equivalent), where applicable.	Not applicable
16. For phase II and III randomized controlled trials, please refer to the CONSORT flow diagram (see link list at top right) and submit the CONSORT checklist (see link list at top right) with your submission. See author guidelines, under 'Reporting Guidelines'. Please confirm you have submitted this list.	Not applicable
17. For tumor marker prognostic studies, we recommend that you follow the REMARK reporting guidelines (see link list at top right). See author guidelines, under 'Reporting Guidelines'. Please confirm you have followed these guidelines.	Not applicable

F- Data Accessibility

18: Provide a "Data Availability" section at the end of the Materials & Methods, listing the accession codes for data generated in this study and deposited in a public database (e.g. RNA-Seq data: Gene Expression Omnibus GSE39462, Proteomics data: PRIDE PXD000208 etc.) Please refer to our author guidelines for 'Data Deposition'.<br><br>Data deposition in a public repository is mandatory for:<br>a. Protein, DNA and RNA sequences<br>b. Macromolecular structures<br>c. Crystallographic data for small molecules<br>d. Functional genomics data<br>e. Proteomics and molecular interactions	Provided in the Data Avaliability Section
19. Deposition is strongly recommended for any datasets that are central and integral to the study; please consider the journal's data policy. If no structured public repository exists for a given data type, we encourage the provision of datasets in the manuscript as a Supplementary Document (see author guidelines under 'Expanded View' or in unstructured repositories such as Dryad (see link list at top right) or Figshare (see link list at top right).	Provided in the Data Avaliability Section
20. Access to human clinical and genomic datasets should be provided with as few restrictions as possible while respecting ethical obligations to the patients and relevant medical and legal issues. If practically possible and compatible with the individual consent agreement used in the study, such data should be deposited in one of the major public access-controlled repositories such as dbGAP (see link list at top right) or EGA (see link list at top right).	Not applicable.
21. Computational models that are central and integral to a study should be shared without restrictions and provided in a machine-readable form. The relevant accession numbers or links should be provided. When possible, standardized format (SBML, CellML) should be used instead of scripts (e.g. MATLAB). Authors are strongly encouraged to follow the MIRIAM guidelines (see link list at top right) and deposit their model in a public database such as Biomodels (see link list at top right) or JWS Online (see link list at top right). If computer source code is provided with the paper, it should be deposited in a public repository or included in supplementary information.	Provided as a Supplementary Software. Links provided to the Github ibraries in the Data Availability Section

G- Dual use research of concern

22. Could your study fall under dual use research restrictions? Please check biosecurity documents (see link list at top right) and list of select agents and toxins (APHIS/CDC) (see link list at top right). According to our biosecurity guidelines, provide a statement only if it could.	Not applicable.
